# Joint optimization of smart inverters and EV charging coordination for enhanced DG-EV hosting capacity under uncertain conditions for resilient distribution systems

**DOI:** 10.1371/journal.pone.0350725

**Published:** 2026-07-06

**Authors:** Faisal Aldawsari, Zenhom M. Zenhom, Ziad M. Ali, Shady H. E. Abdel Aleem

**Affiliations:** 1 Electrical Engineering Department, College of Engineering at Wadi Addawaser, Prince Sattam bin Abdulaziz University, Wadi Addawaser, Saudi Arabia; 2 Electric Power Engineering Department, Ahram Canadian University, Giza, Egypt; 3 Department of Electrical Engineering, Faculty of Engineering, Badya University, Giza, Egypt; 4 Basic Sciences Council, Academy of Scientific Research and Technology, Cairo, Egypt; Aalto University, FINLAND

## Abstract

The rapid growth of renewable-based distributed generation (DG) and electric vehicles (EVs) poses significant operational challenges for distribution systems (DSs), particularly under uncertainties in renewable output, load demand, and EV charging behavior. Distribution system operators must therefore evaluate and enhance both DG hosting capacity (DG-HC) and EV hosting capacity (EV-HC) while maintaining voltage security and reducing losses. This study presents a stochastic, multi-objective optimization framework that jointly coordinates smart inverter (SI)-based Volt/VAR control and EV charging scheduling to simultaneously maximize DG-HC and EV-HC and minimize active power losses and voltage deviation. The framework integrates active power management through EV charging coordination and reactive power support via optimally deployed SIs. The resulting multi-objective problem is solved using the Starfish Optimization Algorithm (SFOA) and benchmarked against three established metaheuristics. The methodology is validated on the IEEE 33-bus system and a real 59-bus distribution network in Cairo, Egypt. Results show that coordinated SI–EV control increases DG-HC and EV-HC by up to 74% and 89%, respectively, and achieves voltage deviation reductions of 55% in the IEEE 33-bus system and 11% in the Cairo DS. Comparative analysis confirms that SFOA provides superior convergence and solution quality relative to the competing techniques.

## 1 Introduction

### 1.1 Motivation

Global warming, primarily driven by greenhouse gas emissions such as CO₂, CH₄, and N₂O, poses a profound threat to sustainability by triggering extreme weather events, disrupting ecosystems, and accelerating biodiversity loss [1]. Mitigating these adverse impacts requires not only a significant reduction in emissions but also the adoption of alternative energy sources, large-scale electrification of the transportation sector, and coordinated global action to secure a sustainable future. Electricity production remains a dominant contributor to global greenhouse gas emissions, responsible for nearly 42% of worldwide emissions in 2015, largely due to the heavy dependence on fossil fuels, particularly coal, which has the highest carbon intensity among energy sources [2]. The transportation sector ranks second, accounting for roughly one-quarter of total global emissions [3].

Against this backdrop, governments and societies have increasingly promoted distributed renewable energy integration and electric mobility as key pillars for advancing sustainability and alleviating climate pressures. Consequently, recent years have witnessed a rapid expansion in the deployment of renewable energy sources (RESs) and electric vehicles (EVs) within distribution systems (DSs) [4]. However, the large-scale penetration of RESs and EVs introduces new operational complexities, including sudden power surges, uncertainties in demand and generation, and the urgent requirement for advanced load management strategies to ensure reliable and economically efficient grid performance [5].

Ensuring the sustainable and robust operation of modern distribution systems requires distribution system operators (DSOs) to rigorously quantify the upper limits of renewable energy source (RES) penetration, defined as distributed generation hosting capacity (DG-HC), as well as the permissible integration level of EVs, known as EV hosting capacity (EV-HC) [6].

### 1.2 Literature review

Achieving the ambitious objective of enhancing both DG-HC and EV-HC necessitates a coordinated blend of behavioral, policy-driven, and technical interventions. For power systems to remain resilient while advancing long-term sustainability, particularly amid the accelerating integration of RDGs and EVs, such measures must be applied in a synergistic and holistic manner [7].

Demand-side management (DSM) represents a vital technique that simultaneously benefits consumers, grid operators, and the environment by optimizing energy consumption, improving system reliability, and increasing the hosting capacity of both RESs and EVs [6]. In recent years, Demand Response (DR) programs have emerged as a cost-effective and efficient alternative to conventional infrastructure upgrades, such as distribution network reinforcement, capacitor bank installation, and cable replacement, providing a sustainable pathway for enhancing hosting capacity (HC) without incurring significant capital investments [8]. Among controllable loads, plug-in EVs hold significant potential for DSM, driven by their rapidly growing penetration in modern DSs. For example, global EV sales surged from fewer than 10,000 units in 2010 to approximately 774,000 units in 2016, surpassing a cumulative total of 2 million vehicles. Furthermore, in 2020, the Electric Vehicle Initiative (EVI) set an ambitious target of expanding the global EV fleet to 20 million units [9].

To harness this potential, numerous DSM strategies leveraging EVs have been developed. In [10], the authors assessed the HC of wind-based RESs using a stochastic bi-level optimization framework. The upper level is designed to maximize HC through the optimal operation of on-load tap changers (OLTCs), while the lower level minimizes power losses by coordinating the charging schedules of EVs. The findings demonstrate that effective EV charging management can increase HC by up to 15% compared with uncoordinated charging strategies. In a related study [11], a two-layer optimization framework was proposed to enhance the HC of wind-based RESs by jointly coordinating the EV charging process, OLTC operation, and reactive power control of wind turbine inverters. The findings highlighted that the integrated coordination of these elements delivers the most significant improvement in HC. Although such works laid the groundwork in this field, their scope remained constrained to wind-based RESs, overlooking the broader integration potential of diverse distributed generation technologies. In addition, authors in [12] develops a hybrid game-theoretic scheduling framework for multi-integrated energy microgrids, using TSSRO and SNCGAN-generated scenarios to manage economic interactions, profit allocation, and multi-energy uncertainties. Its focus lies in coordinated economic dispatch and robust market-oriented operation, solved through an ADMM-based single-level reformulation. By contrast, our work targets a different problem layer-technical HC enhancement in distribution networks, emphasizing voltage regulation, active/reactive coordination, and uncertainty-driven DG–EV accommodation rather than market games or profit-based scheduling. Additionally, authors in [13] develops a mixed-integer linear programming (MILP) framework that maximizes PV hosting capacity in unbalanced active distribution networks by jointly optimizing DGs dispatch, EV charging coordination, switched capacitor banks, energy storage, and demand-response actions. In contrast, our work departs from deterministic MILP-based coordination and adopts a stochastic, multi-objective optimization framework that explicitly captures uncertainties in renewable generation, load demand, and EV behavior, while jointly maximizing both DG-HC and EV-HC under smart-inverter Volt/VAR control. This enables the identification of uncertainty-driven HC limits. Additionally, in [14], the authors applied a stochastic approach to assess the voltage-constrained HC of PV-based RESs. The proposed method incorporates EV charging coordination to enhance PV-HC. Results indicate that permitting EV demand to reach 12% of the peak load at each PV installation can increase the PV-HC of the IEEE 123-bus DS by approximately 4%. In [15], the authors investigated the influence of EV charging coordination on the HC of different RES technologies under varying load profiles. The study revealed that although residential EV charging elevates the peak demand of the DS, its impact on DG-HC remains relatively modest. Furthermore, the extent to which charging station loads affect DG-HC is strongly dependent on the type of DG technology employed. For instance, when DGs are based on PV units, increasing the charging station load to its maximum level enhances DG-HC by 1.81%. Conversely, in the case of biomass- or wind-based DGs, such load growth does not substantially improve the DS’s DG-HC. Besides, [16] presented a stochastic multi-objective framework that jointly considers wind-based distributed generation and EV charging coordination to enhance HC while reducing power losses. Although validated on the IEEE 33-bus network with promising results, the absence of reactive power management constrained the achievable improvements in HC and overall technical performance. In addition, authors in [17] present a multi-objective scheduling framework for active distribution networks that integrates large-scale EV charging demands, quantified using Monte Carlo sampling, to minimize operating costs, reduce net-load variability, and maximize PV utilization. Using an enhanced NSGAII-NDAX algorithm with fuzzy decision-making for Pareto solution selection, the study demonstrates that coordinated EV charging can effectively flatten load profiles and improve PV consumption on an improved IEEE 33-node system. In contrast, this work introduces a stochastic, SI-enabled HC framework that jointly maximizes DG-HC and EV-HC under renewable, load, and EV uncertainties, addressing voltage improvement and capacity expansion limits that the scheduling-focused study does not consider.

Smart inverter (SI)-based Volt/VAR control has been widely investigated as a means of improving HC. Early studies [18–21] focused exclusively on enhancing DG-HC, while later works [22,23] explored its integration with complementary enhancement strategies. For example, authors in [21] employed a particle-swarm-based coordination of smart-inverter Volt/VAR control to enhance PV HC in an Ecuadorian distribution network, reporting an improvement of approximately 46%. Similarly, the authors in [18] examined how optimally tuning the Volt/VAR response of SIs mitigates overvoltage issues, thereby allowing greater PV penetration. In [22], Volt/VAR control was combined with a DSM program—specifically, time-of-use (TOU) pricing—to address technical challenges at the point of common coupling (PCC) under high PV penetration, such as overvoltage, increased power losses, and voltage fluctuations. Addressing these issues enabled further enhancement of PV-HC. To the best of the authors’ knowledge, [8,22,24] remain the only studies that explicitly integrate SI Volt/VAR control with DSM strategies for HC improvement. By contrast, [23] proposed a multi-objective optimization framework where SI Volt/VAR control was jointly applied to PV units and battery energy storage systems (BESSs). The objectives included maximizing PV-HC and minimizing voltage deviations, and the optimization was solved using the slime mold algorithm (SMA). Despite these valuable contributions, notable gaps persist. These studies neglected the inherent uncertainties in both generation and load demand, which compromise the robustness of their approaches and restrict the practical applicability of the results. In addition, authors in [25] developed an integrated multi-objective framework that jointly optimizes energy storage system (ESS) planning and heterogeneous EV scheduling to support wind–solar integration, using Monte Carlo simulation for EV uncertainty and an improved MOPSO solver. Its primary focus is on cost minimization, renewable integration rate, and power-deficit reduction in systems with coordinated ESS and EV operations. In contrast, our work targets a different technical objective, quantifying and enhancing DG and EV hosting capacities in distribution networks under uncertainty, by coordinating SI-based Volt/VAR control and EV charging to ensure voltage security and loss minimization rather than ESS planning or cost-driven renewable integration. The simultaneous assessment and enhancement of DG-HC and EV-HC is a highly complex task, as both are shaped by stochastic and interdependent factors such as renewable generation variability, load demand fluctuations, and EV charging behaviors. These uncertainties, together with operational constraints, including voltage regulation, network losses, and power quality, make joint optimization particularly challenging yet crucial for achieving reliable and sustainable distribution system performance. Although both DG-HC and EV-HC have been widely examined in the literature, they are predominantly studied in isolation, with little attention given to their mutual interactions. To date, only [4,7,8,26], have explicitly attempted to optimize DG-HC and EV-HC simultaneously, underscoring a significant gap in existing research. In 2020, the authors in [4] introduced a framework for evaluating the joint HC of PV and EVs. They introduced methods for assessing PV–EV HC, showing that EV charging coordination mainly benefits EV-HC, while PV curtailment primarily enhances PV-HC. This study was limited to one DG technology, PV only. Later, [26] expanded this work by including PV and wind RESs, reporting a 9% increase in DG-HC through regulated EV aggregator charging compared with uncontrolled EV charging scenarios. Both studies employed a common enhancement strategy—EV charging coordination combined with active power curtailment—to boost joint hosting capacity. More recently, the authors in [7] proposed a coordinated approach integrating demand response with transmission expansion, which enhanced the minimum DG hosting capacity and substantially increased EV hosting capacity during peak-demand periods. Additionally, the authors in [8] proposed an innovative framework for enhancing both DG-HC and EV-HC, employing a synergistic strategy that integrates a dynamic tariff-based demand-side management program with smart inverter control. The omission of generation and load uncertainties in previous studies constitutes a significant research gap, underscoring the need for future methodologies to explicitly incorporate these factors to achieve more robust and practically applicable results. However, some recent studies have considered multi-objective coordination models that explicitly incorporate carbon-emission mechanisms. For example, the work in [27] proposed a day-ahead dispatch strategy for park-level integrated energy systems that integrates a reward–penalty carbon trading mechanism, coordinated carbon-capture operation, and flexibility indicators, reformulated into an MILP to balance economic cost, carbon cost, and operational flexibility. In contrast, our study focuses on a different layer of the power system and a different objective: enhancing DG and EV HCs in distribution networks under renewable and EV uncertainty. Rather than optimizing carbon economics, our framework focuses on technical feasibility, voltage regulation, loss minimization, and coordinated SI Volt/VAR and EV charging control, providing a complementary contribution to increase renewable and EV accommodation capability at the distribution-system level. Table 1 highlights the key contributions of this work in contrast to existing studies in the literature.

**Table 1:** Overview of the key contributions of this study in comparison with selected works from the literature.

Assessment Parameters		[10], [16]	[11], [15]	[14]	[18]	[19–21]	[23]	[22]	[25]	[4]	[13]	[26]	[8]	This work
HC evaluation		Y	Y	Y	N	Y	Y	Y	N	Y	Y	Y	Y	Y
System uncertainties		Y	Y	Y	N	N	Y	N	Y	N	N	N	N	Y
DG technology variety		N	N	N	N	N	N	N	Y	N	N	Y	Y	Y
Active power support		Y	Y	Y	N	Y	Y	Y	Y	Y	Y	Y	Y	Y
Reactive power support		N	Y	N	Y	N	Y	Y	N	N	Y	N	Y	Y
EVs charging scheduling		Y	Y	Y	N	N	N	N	Y	Y	Y	Y	Y	Y
Optimization objectives	DG-HC maximization	Y	Y	Y	N	Y	Y	Y	N	Y	Y	Y	Y	Y
	EV-HC maximization	N	N	N	N	N	N	N	Y	Y	N	Y	Y	Y
	Loss reduction	Y	N	N	N	N	N	N	N	N	Y	Y	Y	Y
	Voltage profile enhancement	N	N	N	Y	N	Y	N	N	N	N	N	N	Y

### 1.3 Novelty and contributions

**Fig 1:**
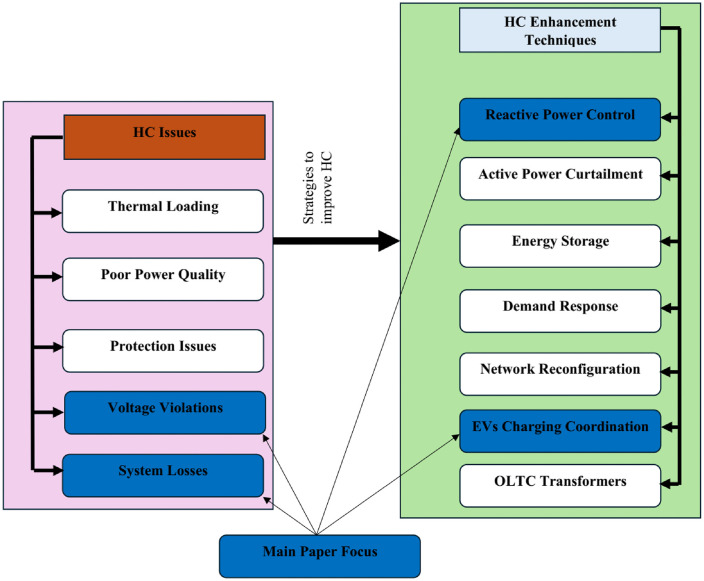
Hosting capacity challenges and the corresponding solutions proposed in prior studies.

To the best of the current knowledge, no prior work has simultaneously optimized DG-HC, EV-HC, system losses, and voltage deviation while explicitly addressing uncertainties in renewable generation, load demand, and EV behavior. This study proposes an integrated framework that combines optimal sizing of PV and wind units, coordinated management of aggregated EV charging, and advanced Volt/VAR control of Smart Inverters (SIs). Active power flexibility is harnessed through EV charging coordination, while reactive power support is provided via inverter control, enabling both independent and hybrid enhancement strategies. The hybrid approach demonstrates superior performance in maximizing DG-HC and EV-HC, improving voltage profiles, and reducing network losses across diverse planning and operational scenarios. Uncertainty in solar radiation, wind speed, load demand, and EV usage is modeled using probability density functions (PDFs) and captured through Monte Carlo (MC) simulations, with scenario reduction achieved via the Kantorovich distance method. Fig 1 presents the overall conceptual framework of the proposed methodology. The novel aspects and key contributions of this work are as follows:

- Evaluation of the effectiveness of integrating optimal SI Volt/VAR control with coordinated EV charging strategies to enhance the joint HC of DGs and EVs (DG-EV-HC).
- A robust stochastic, multi-objective, time-varying optimization framework that jointly coordinates the PV- and wind-based RESs, EV charging management, and SI Volt/VAR control to maximize the integrated DG-EV-HC.
- Uncertainty in critical factors such as load demand, solar irradiance, wind speed, and EV operational behavior is systematically modeled by generating scenarios with Monte Carlo simulations and refining them through reduction methods.
- The capability of the recently proposed Starfish Optimization Algorithm (SFOA) in solving the complex optimization framework is assessed, with its performance compared to several established heuristic methods.
- Extensive simulations are conducted on the IEEE 33-bus test feeder and a practical 59-bus distribution grid in Cairo, confirming the practicality and scalability of the developed methodology.

### 1.4 Paper organization

The structure of the paper is arranged as follows. Section 2 discusses in detail the approaches used for modeling uncertainties in load demand, solar irradiance, wind speed, and EV charging patterns. Section 3 introduces the essential concepts of the SFOA optimization method. Section 4 describes the formulation of the proposed framework. Section 5 reports on test networks, case investigations, simulation outcomes, performance analysis, and comprehensive discussions. Lastly, Section 6 concludes the key findings and provides recommendations for future research directions.

## 2 Modeling of uncertainty

Incorporating various forms of distributed generation, particularly RESs and plug-in EVs, into present-day DSs introduces notable operational complexities due to their fluctuating and unpredictable nature. To cope with these uncertainties, probabilistic modeling techniques have become an essential tool, offering a pathway toward enhancing both the reliability and economic efficiency of distribution network operation [28]. The next subsections introduce the stochastic methodologies used to manage the uncertainties arising from variations in load consumption, solar irradiance, wind conditions, and the operational patterns of plug-in EVs. PV, wind, load, and EV processes share the same hourly time index, reflecting realistic dependence driven by weather and time-of-day patterns. Within a given scenario, all processes evolve consistently without explicit covariance matrices; instead, dependence is captured implicitly through common driving profiles (irradiance, wind speed, base load, and EV charging behavior). This is consistent with standard distribution-system planning practice.

### 2.1 Solar radiation and PV power uncertainties

The output of PV-based RESs is highly dependent on the level of solar irradiance. Fluctuations in sunlight, mainly caused by cloud movement, create uncertainty in PV generation. Such randomness is commonly captured through probabilistic representation, with the beta distribution often employed for this purpose and expressed as [29]:


$$\begin{array}{c} f\left(I_{k}\right)=\frac{\Gamma (a_{k}+b_{k})}{\Gamma (a_{k})\Gamma (b_{k})}\;{\left(\frac{I_{k}}{I_{max,k}}\right)}^{a_{k}-1}{\left(1-\frac{I_{k}}{I_{max,k}}\right)}^{b_{k}-1}\;0\leq \frac{I_{k}}{I_{max,k}}\leq 1,\;a_{k}\geq 0,\;b_{k}\geq 0 \end{array}$$ (1)

where $f\left(I_{k}\right)$ represents the beta distribution at time *k*, $I_{k}$, $I_{max,k}$ are solar irradiance along with the highest solar irradiance at instant *k*. $a_{k}$, $b_{k}$ introduce the shape parameters of the beta distribution. These shape parameters of the beta distribution have originated from the statistical features, precisely, the mean ($\mu_{k}$) and standard deviation ($\sigma_{k}$), of the normalized solar radiation ($\frac{I_{k}}{I_{max,k}}$) as follows:


$$\begin{array}{c} a_{k}=\mu_{k}\left(\frac{\mu_{k}\left(1+\mu_{k}\right)}{\overset{2}{\underset{k}{\sigma }}}-1\right) \end{array}$$ (2)

$$\begin{array}{c} b_{k}=(1-\mu_{k})(\frac{\mu_{k}\left(1+\mu_{k}\right)}{\overset{2}{\underset{k}{\sigma }}}-1) \end{array}$$ (3)

Concerning PV-based RESs generated power, (4) demonstrates the output power $P_{PV}$ concerning a given solar radiation I, where $I_{std}$, $I_{c}$ represent the solar irradiance under standard test conditions, along with the specified operating irradiance level, correspondingly [30].


$$\begin{array}{c} P_{PV}=\left\{ \begin{array}{c} \overset{PV}{\underset{rat}{P}}\left(\frac{I^{2}}{I_{std}I_{c}}\right)\;0<I<I_{c}\\ \overset{PV}{\underset{rat}{P}}\left(\frac{I}{I_{std}}\right)\;I\geq I_{c} \end{array}\right. \end{array}$$ (4)

PV output is modeled using a lightweight piecewise surrogate: quadratic for irradiance below $I_{c}$ (W/m²) and linear above, scaled by an effective rated power that embeds temperature and design derating. Clipping is enforced by the PV-rated power bound. Since HC limits occur mainly during high irradiance, the low-irradiance curvature has a negligible effect on feeder-level HC results. The framework could accommodate more detailed PV models if desired.

### 2.2 Wind speed and wind power uncertainties

Wind speed is characterized by random fluctuations and unpredictability, primarily driven by varying weather conditions and site-specific geographical factors. To represent this stochastic behaviour, probabilistic techniques are commonly applied, among which the Weibull distribution is one of the most prevalent and effective models. The Weibull probability model, characterized by two defining parameters, $\lambda_{k}$ is the shape parameter and $\theta_{k}$ is the scale parameter, is widely used to describe wind speed behavior at a given time instant *k* [31].


$$\begin{array}{c} f\left(\omega_{k}\right)=\frac{\lambda_{k}}{\theta_{k}}\;{\left(\frac{\omega_{k}}{\theta_{k}}\right)}^{\lambda_{k}-1}\exp\left[{\left(-\frac{\omega_{k}}{\theta_{k}}\right)}^{\lambda_{k}}\right]\;0\leq \omega_{k}\leq \infty \end{array}$$ (5)

where $\omega_{k}$ is the wind speed at time *k*. The form parameter dictates the distributional pattern of wind speed fluctuations, whereas the scale factor specifies its representative intensity level. Accordingly, the cumulative representation of the Weibull model can be formulated as [31]:


$$\begin{array}{c} F\left(\omega_{k}\right)=\int_{0}^{\omega_{k}}f\left(\omega_{k}\right)d\omega_{k}=1-exp{\left(-\frac{\omega_{k}}{\theta_{k}}\right)}^{\lambda_{k}} \end{array}$$ (6)

Wind speed directly affects WT WT-generated power. Using (7) [30], the active power produced ($P_{WT}$) might be specified with respect to wind speed (*ω*). In this context, $\omega_{cut-in}$, $\omega_{rated}$ and $\omega_{cut-out}$ represent the WT’s cut-in, rated, and cut-out wind speeds, correspondingly, while $\overset{WT}{\underset{rat}{P}}$ represents the WT’s rated power capacity.


$$\begin{array}{c} P_{WT}=\left\{ \begin{array}{c} \;\\ 0\;\omega <\omega_{cut-in}\\ \overset{WT}{\underset{rat}{P}}\left(\frac{\;\omega -\omega_{cut-in}}{\omega_{rated}-\omega_{cut-in}}\right)\;\omega_{cut-in}\leq \omega \leq \omega_{rated}\\ \overset{WT}{\underset{rat}{P}}\;\omega_{rated}\leq \omega \leq \omega_{cut-out}\\ 0\;\omega >\omega_{cut-out} \end{array}\right. \end{array}$$ (7)

### 2.3 Load demand uncertainties

Incorporating multiple RESs into DSs amplifies the uncertainty associated with load demand forecasting, making consumption profiles more irregular and harder to anticipate. To characterize this randomness practically, the demand is represented through a truncated normal distribution, which restricts fluctuations within predefined upper and lower limits. This probabilistic treatment offers a credible depiction of demand uncertainty by constraining values to physically acceptable ranges while maintaining statistical consistency. The corresponding mathematical expression of the truncated normal model for load demand is given as [32]:


$$\begin{array}{c} \Omega \left(\mu^{D},\sigma^{D},\overset{D}{\underset{min}{\chi }},\overset{D}{\underset{max}{\chi }},\overset{D}{\underset{i,k}{\chi }}\right)=\left\{ \begin{array}{c} @r\text{0}\;\overset{D}{\underset{i,k}{\chi }}<\overset{D}{\underset{min,k}{\chi }}\;or\;\overset{D}{\underset{i,k}{\chi }}>\overset{D}{\underset{max,k}{\chi }}\;\\ \frac{\Phi (\overset{D}{\underset{k}{\mu }},\overset{D}{\underset{k}{\sigma }},\;\overset{D}{\underset{i,k}{\chi }})}{\Phi \left(\overset{D}{\underset{k}{\mu }},\overset{D}{\underset{k}{\sigma }},\;\overset{D}{\underset{max}{\chi }}\right)-\Phi (\overset{D}{\underset{k}{\mu }},\overset{D}{\underset{k}{\sigma }},\;\overset{D}{\underset{min}{\chi }})}\;\overset{D}{\underset{min,k}{\chi }}<\overset{D}{\underset{i,k}{\chi }}<\overset{D}{\underset{max,k}{\chi }}\; \end{array}\right.\; \end{array}$$ (8)

where “*Φ*” denotes the cumulative distribution function (CDF) of a normal distribution, $\overset{D}{\underset{k}{\mu }}$ denotes the mean of the load consumption at time *k*, $\overset{D}{\underset{k}{\sigma }}$ denotes the standard deviation of the load consumption at time *k*, $\overset{D}{\underset{i,k}{\chi }}\;$means load factor (normalized load demand) for bus *i* at time *k*. The boundaries of the load factor are presented in the following equation:


$$\begin{array}{c} \overset{D}{\underset{i,k}{\chi }}\in [\overset{D}{\underset{min,k}{\chi }},\;\overset{D}{\underset{max,k}{\chi }}]\in [\;\overset{D}{\underset{k}{\mu }}-\overset{D}{\underset{k}{\sigma }},\overset{D}{\underset{k}{\mu }}+\overset{D}{\underset{k}{\sigma }}] \end{array}$$ (9)

Based on historical demand records, the expected value, variability, and admissible bounds are extracted, and these parameters are then employed to generate the nominal load profile using a truncated normal probability distribution, as formulated below:

repeat:


$$\begin{array}{c} x=rand\left(\;\right) \end{array}$$

$\overset{D}{\underset{i,k}{\chi }}={𝛷}^{-1}(\overset{D}{\underset{k}{\mu }},{\left(\overset{D}{\underset{k}{\sigma }}\right)}^{2},x)$


$$\begin{array}{c} until\;(\overset{D}{\underset{min,k}{\chi }}<\overset{D}{\underset{i,k}{\chi }}<\overset{D}{\underset{max,k}{\chi }}) \end{array}$$ (10)

### 2.4 Electric vehicles’ load uncertainties

The electricity consumption of EV users is largely determined by their driving schedules as well as charging practices, all of which stem from personal behavioural tendencies and therefore differ widely across individuals. This variability introduces uncertainty, which can be characterized using essential factors such as the state of charge (*SOC*), arrival time, and departure time. As described below, these factors are modelled as stochastic variables and represented through a truncated normal distribution [33].


$$\begin{array}{c} SO\overset{in}{\underset{i}{C}}=g_{TND}(X,\mu_{SOC},\overset{2}{\underset{SOC}{\sigma }},\;(SO\overset{in}{\underset{min}{C}},\;SO\overset{in}{\underset{max}{C}})) \end{array}$$ (11)

$$\begin{array}{c} \overset{ar}{\underset{i}{\tau }}=g_{TND}(X,\mu_{ar},\overset{2}{\underset{ar}{\sigma }},\;\left(\overset{min}{\underset{ar}{\tau }},\;\overset{max}{\underset{ar}{\tau }}\right)) \end{array}$$ (12)

$$\begin{array}{c} \overset{dep}{\underset{i}{\tau }}=g_{TND}(X,\mu_{dep},\overset{2}{\underset{dep}{\sigma }},\;\left(\overset{min}{\underset{dep}{\tau }},\;\overset{max}{\underset{dep}{\tau }}\right)) \end{array}$$ (13)

where $g_{TND}$ denotes the truncated normal distribution function. Moreover, $SO\overset{in}{\underset{i}{C}},\;\overset{ar}{\underset{i}{\tau }},\;and\;\overset{dep}{\underset{i}{\tau }}$ denotes the initial value of *SOC*, the arrival and departure time of EV at customer *i*. Individual EVs are not simulated; instead, aggregate hourly EV charging-demand trajectories are generated from probabilistic arrival, departure, and SOC distributions and included in the 1000 Monte Carlo samples. After scenario reduction, the optimization uses these aggregate EV profiles directly. This feeder-level modeling approach is suitable for hosting-capacity assessment and eliminates the need for per-vehicle SOC dynamics, which fall outside the study’s scope.

The discussed stochastic schemes employ a probabilistic framework to handle load demand, EVs behavior, and the output power of multiple DG units by fitting historical hourly data to appropriate probability distributions for parameter estimation. Using these estimated parameters, random variables are generated according to their respective distributions across multiple scenarios to capture uncertainty. Scenario generation is performed via Monte Carlo simulation; however, due to the high computational burden associated with large-scale scenario sets, a scenario reduction strategy based on the Kantorovich Distance Matrix (KDM) is applied. This technique prunes redundant and highly similar scenarios while preserving a diverse, statistically representative subset, reducing the original 1000 scenarios to a manageable 10 (in this study) without significant loss of information.

The KDM-based reduction process relies on measuring the distance between scenarios and iteratively eliminating those that contribute least to the dataset’s overall diversity. In this context, each scenario represents a trajectory of uncertain parameters (e.g., load, EV demand, and renewable generation) over the considered time horizon. The reduction procedure follows these steps:

**Stage 1**: Produce the number of scenarios $\xi_{s}$(1,2,….,$N_{s}$), then estimate the distance among all scenarios $D(\xi_{s},\xi_{s^{\prime }})$=$\left|\xi_{s}-\xi_{s^{\prime }}\right|,\;s,s^{\prime }=1,2,\ldots ..,N_{s}$ and set an initial probability to each scenario $\pi_{s}=\frac{1}{N_{s}}$. The initial probability for each scenario is calculated in this step simply by assuming that all generated scenarios are equally likely before any reduction is applied.
**Stage 2**: The algorithm identifies the two scenarios that are most similar to each other based on their distance. This can be formulated as: $D\left(s,s^{\prime }\right)$=$\min D\left(\xi_{s},\xi_{s^{\prime }}\right),\;s,s^{\prime }\in \xi_{s}$, $s\neq s^{\prime }.$
**Stage 3**: The algorithm chooses *s* as the one with the smaller distance, calculated as $s=\pi_{s}\min D\left(\xi_{s},\xi_{s^{\prime }}\right)$, then remove the weaker scenario among the collection of scenarios, the updated set of scenarios becomes $S^{\prime }=S\backslash s$, the probability of the deleted scenario *s* is added to the closest remaining scenario $s^{\prime }$ as $\overset{\prime }{\underset{s}{\pi }}=\overset{\prime }{\underset{s}{\pi }}+\pi_{s}$.
**Stage 4**: Once the required number of reduced scenarios has been reached, repeat Steps 2 and 3.

**Fig 2:**
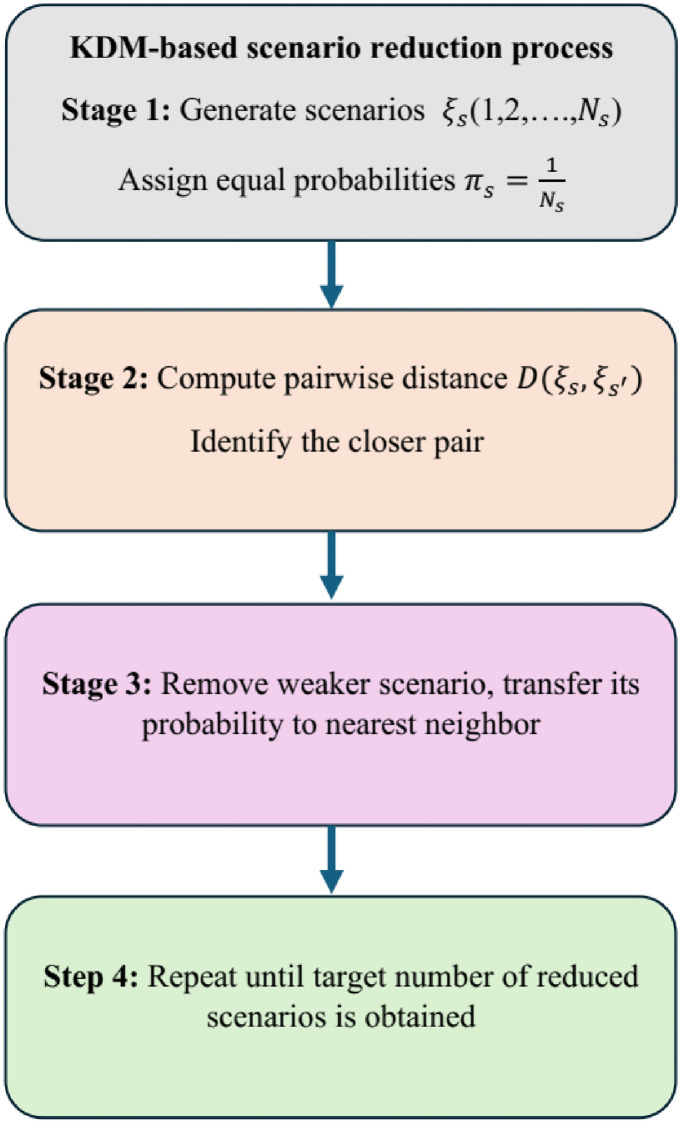
Main steps of the KDM-based scenario reduction procedure applied to Monte Carlo–generated dataset.

Through this iterative clustering and probability redistribution process, the KDM method ensures that the reduced scenario set maintains both statistical representativeness and computational tractability, making it suitable for large-scale stochastic optimization problems. Fig 2 illustrates the main steps in the KDM-based reduction procedure as explained in [34].

## 3 Starfish Optimization Algorithm (SFOA)

### 3.1 Biological inspiration

Starfish, or sea stars, are marine echinoderms distinguished by their star-shaped bodies, typically possessing five radiating arms connected to a central disk. These arms not only facilitate locomotion but also host sensory organs that aid in navigation and food detection. Starfish exhibit three primary behaviors: (i) exploration, (ii) preying, and (iii) regeneration, which directly inspire the design of the SFOA. When foraging, starfish collaboratively locate prey, wrap their arms around it, and extend their stomachs externally for digestion. Their remarkable regenerative ability allows them to reproduce or recover lost limbs, ensuring survival and adaptability in dynamic environments. These natural mechanisms of distributed sensing, cooperative hunting, and self-regeneration are conceptually mirrored in the SFOA to achieve an effective balance between global exploration and local exploitation during optimization [35].

### 3.2 Mathematical framework

The SFOA, like most population-based metaheuristics, is structured around two key phases: the exploration stage, which encourages a global search across the solution domain, and the exploitation stage, which fine-tunes the candidate solutions near promising regions [35].

#### 3.2.1 Initialization.

A population of *N*_*p*_ starfish is initially distributed randomly within the feasible boundaries of each decision variable. The position matrix of all candidates can be defined as:


$$\begin{array}{c} \chi =\left[\begin{array}{cccc} \chi_{11} & \chi_{12} & \ldots & \chi_{1D}\\ \chi_{21} & \chi_{22} & \ldots & \chi_{2D}\\ \vdots & \vdots & \vdots & \vdots \\ \chi_{N_{p}1} & \chi_{N_{p}2} & \ldots & \chi_{N_{p}D} \end{array}\right] \end{array}$$ (14)

where χ is the $N_{p}\times D$ sized matrix to record starfish positions, *D* is the dimension of the decision variables. During the initialization stage, the location of every starfish, formulated in (15), is determined as follows:


$$\begin{array}{c} \chi_{mn=LB_{n}+r(UB_{n}-LB_{n})} \end{array}$$ (15)

Here, $\chi_{mn}$ represents the position of the *m*th starfish along the *n*th dimension, *r* is a random variable uniformly distributed in the range (0, 1), while $UB_{n}$ and $LB_{n}$ denote the maximum and minimum permissible limits of the decision variable in the *n*th dimension, respectively.

#### 3.2.2 Exploration phase.

SFOA applies a hybrid search pattern that merges 5-dimensional and 1-dimensional movement strategies. When the problem dimension (*D* ≥ 5), the starfish explores in five randomly selected dimensions, representing its five arms; otherwise, it performs a one-dimensional search. This adaptive approach improves computational efficiency and avoids premature convergence observed in classical vector-based searches. Regarding *D* ≥ 5, update of the position, in this stage, is governed by:


$$\begin{array}{c} \overset{i}{\underset{m,p}{Y}}=\left\{ \begin{array}{c} \overset{i}{\underset{m,p}{\chi }}+\lambda \left(\overset{i}{\underset{best,p}{\chi }}-\overset{i}{\underset{m,p}{\chi }}\right)cos\theta \;r\leq 0.5\\ \overset{i}{\underset{m,p}{\chi }}-\lambda \left(\overset{i}{\underset{best,p}{\chi }}-\overset{i}{\underset{m,p}{\chi }}\right)sin\theta \;r>0.5 \end{array}\right. \end{array}$$ (16)

Here, $\overset{i}{\underset{m,p}{Y}}\;$represents the updated position of the *m*th starfish. In addition, λ is a random scaling factor controlling the movement amplitude, and θ gradually decreases with iterations to shift from exploration toward exploitation. Regarding *D* < 5, the position update in this stage is governed by:


$$\begin{array}{c} \overset{i}{\underset{m,p}{Y}}=E_{t}\overset{i}{\underset{m,p}{\chi }}+\rho \left(\overset{i}{\underset{k_{1},p}{\chi }}-\overset{i}{\underset{m,p}{\chi }}\right)+\psi \left(\overset{i}{\underset{k_{2},p}{\chi }}-\overset{i}{\underset{m,p}{\chi }}\right) \end{array}$$ (17)

where, $\overset{i}{\underset{k_{1},p}{\chi }}$ and $\overset{i}{\underset{k_{2},p}{\chi }}$ represent the *p*-dimensional positions from two arbitrarily chosen starfish, correspondingly, ρ and ψ denote two random numbers between (- 1, 1), *p* is the arbitrarily chosen number in the *D* dimensions.

#### 3.2.3 Exploitation phase.

This phase consists of preying and regeneration sub-processes that are employed towards reaching a global solution. Regarding the preying sub-process, candidate starfish refine their positions using a two-directional cooperative search. Each starfish updates its position by simultaneously referencing two peers, steering toward more promising regions while preserving population diversity. This parallel mechanism strengthens local search and avoids stagnation. The position update in this sub-process is governed by:


$$\begin{array}{c} \overset{i}{\underset{m}{Y}}=\overset{i}{\underset{m}{\chi }}+r_{1}d_{m1}+r_{2}d_{m2} \end{array}$$ (18)

where, $r_{1}$ and $r_{2}$ represent arbitrary numbers ranging from 0 to 1, and $d_{m1}$ and $d_{m2}$ are randomly selected from the decision variables dimension *D*. In accordance with the original SFOA formulation explained in [35], the regeneration mechanism is applied only to the worst-ranked starfish (*i* = *N*_*p*_) in each iteration. Instead of full random reinitialization, regeneration is performed using an exponential decay operator that gradually attenuates the position vector as iterations progress, encouraging global exploration while maintaining stability. The updated position is computed as:


$$\begin{array}{c} \overset{i}{\underset{m}{Y}}=exp(-i\times \frac{N_{p}}{T_{max}})\overset{i}{\underset{m}{\chi }} \end{array}$$ (19)

where, *i* is the current iteration, while $T_{max}$ represents the highest number of iterations, and $N_{p}$ represents the population size. Boundary constraints are enforced immediately after regeneration using the original SFOA boundary-repair rule. This ensures consistency between the narrative description, mathematical model, and implementation, enabling reproducibility. The SFOA was executed with population size *N*_*p*_ = 50 and maximum iterations *T*_*max*_ = 300.

The exploration and exploitation phases were performed as described, with the preying sub-process refining candidate positions and regeneration applied to the worst-ranked individual. Hard constraints, including bus voltage limits, feeder ampacity, total active power loss, inverter capacity limits, and inverter characteristic voltages ordering, are enforced using a penalty-based approach. Candidate solutions violating any of these constraints are assigned zero values for the hosting capacities terms (DG-HC and EV-HC), and the objective function is penalized accordingly.

**Fig 3:**
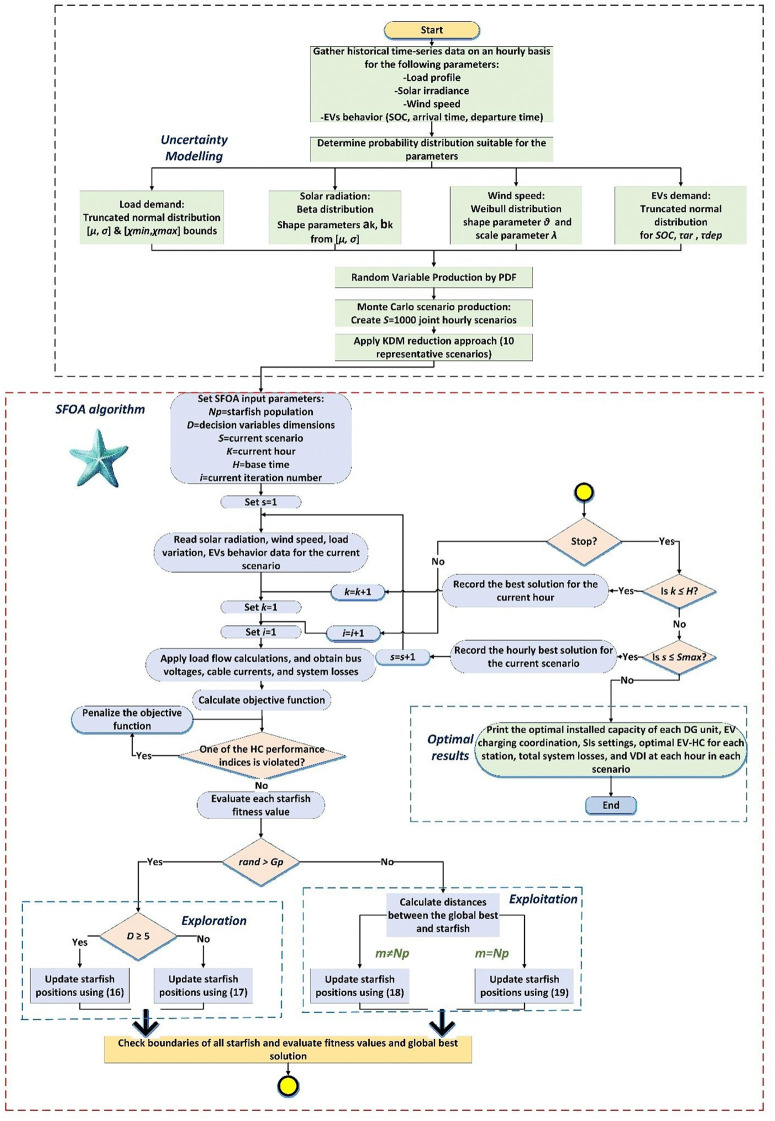
Flowchart explaining the proposed optimization framework based on SFOA.

To validate optimizer performance, 10 independent runs were conducted for hour 13 of scenario 5 (cases 2, 3, and 4) and compared with three other optimizers (see Section 5.3). Based on this assessment, the SFOA was run once per hour per scenario for all other analyses. The stopping criterion was *T*_*max*_ iterations, with no early termination. Fig 3 illustrates the workflow of the proposed stochastic multi-objective optimization framework solved using the SFOA, as follows:

- The upper part of the flowchart depicts the uncertainty modeling process, where historical data of load demand, solar radiation, wind speed, and EV behavior (state of charge, arrival, and departure times) are statistically analyzed to derive suitable probability distributions.
- Monte Carlo simulation generates a large set of random scenarios, which are reduced using the KDM technique to obtain representative cases for computational efficiency.
- The lower part represents the SFOA-based optimization stage, where each reduced scenario is evaluated through power flow analysis to determine bus voltages, system losses, and voltage deviations.
- The algorithm iteratively updates candidate solutions through exploration and exploitation mechanisms until convergence.
- The final outputs include the optimal capacities of distributed generation units, EVs charging stations demand, smart inverter settings, and overall system performance indices for each hour and scenario.

## 4 Problem formulation

This section presents the detailed mathematical framework of the proposed optimization model, outlining the objective functions, decision variables, and system constraints employed to achieve an optimal coordination and control scheme that enhances the combined DG-EV HC within permissible operational boundaries.

### 4.1 Multi-objective function

This study employs a stochastic, time-dependent, multi-objective optimization framework to simultaneously assess the optimal combined hosting capacity (CHC) of distributed generation and electric vehicles under inherent uncertainties. Within this framework, the optimal sizes of PV and WT units, as well as the EV charging stations at predefined locations, are optimized to maximize the CHC while minimizing power losses and voltage deviations. Furthermore, a hybrid active–reactive power management strategy is presented, introducing a synergistic coordination scheme that jointly enhances DG and EV hosting capacities. The proposed scheme integrates EV charging coordination with SIs Volt/VAR control to achieve superior CHC enhancement while keeping the technical performance of the DS. The decision variables in this optimization include the optimal installed capacities of PV- and WT-based DG units, the charging profiles of EV stations, and the control settings of SIs. The multi-objective optimization problem is mathematically formulated as follows.


$$\begin{array}{c} DG-H\overset{k}{\underset{s}{C}}=\frac{\sum_{j=1}^{N}{P_{WT_{s,j}}}^{k}+\;\sum_{j=1}^{M}{P_{PV_{s,j}}}^{k}}{\sum_{j=1}^{N}\overset{rated}{\underset{WT,j}{P}}+\;\sum_{j=1}^{M}\overset{rated}{\underset{PV,j}{P}}} \end{array}$$ (20)

where $DG-H\overset{k}{\underset{s}{C}}$ represents the determined hourly DG-HC at hour *k* of scenario *s*, ${P_{WT_{s,j}}}^{k}$, ${P_{PV_{s,j}}}^{k}$ denote the output power of the *j*th wind-based DG, along with PV-based DG at hour *k* of scenario *s*, respectively. To enforce DG-HC term to be fully normalized through the multi- objective function, the time-varying output of DG units is divided over the summation of units ratings. In addition, *M* and *N* signify the total number of PV units and WT units, respectively.

Regarding EV-HC, Eq (21) reveals that the hourly value of EV-HC at scenario *s* can be assessed by dividing the summation of all time-varying charging stations’ consumption, $\overset{k}{\underset{EV_{s,r}}{P}}$, at this hour, over the summation of all stations’ ratings.


$$\begin{array}{c} EV-H\overset{k}{\underset{s}{C}}=\frac{\sum_{r=1}^{C}\overset{k}{\underset{EV_{s,r}}{E}}}{\sum_{r=1}^{C}\overset{rated}{\underset{EV,r}{E}}} \end{array}$$ (21)

$$\begin{array}{c} \overset{k}{\underset{EV_{s,r}}{E}}=\overset{k}{\underset{EV_{s,r}}{P}}\mathrm{\backslash Updelta}t \end{array}$$ (22)

Moreover, the normalized active power loss index assessed at hour *k* of scenario *s* symbolized as $PL\overset{k}{\underset{s}{I}}$. At a given hour *k* under scenario *s*, this index is obtained by dividing the total active power loss at that condition by the corresponding base-case total active power loss $\overset{BC}{\underset{loss}{P}}$.


$$\begin{array}{c} PL\overset{k}{\underset{s}{I}}=\frac{\overset{k}{\underset{loss_{s}}{P}}}{\overset{BC}{\underset{loss}{P}}} \end{array}$$ (23)

Furthermore, the deviation index of bus voltages from nominal value at hour *k* of scenario *s* can be signified as $D\overset{k}{\underset{s}{I}}$. It is determined by summing the absolute deviations of each bus 𝑖 voltage magnitude (in per unit) from the nominal value of 1 p.u., as expressed below:


$$\begin{array}{c} D\overset{k}{\underset{s}{I}}=\sum_{i=1}^{N_{B}}\left|1-\overset{k}{\underset{s,i}{V}}\right|,\;\forall \;s,\;\forall \;k\; \end{array}$$ (24)

The formulated time-dependent, scenario-based multi-objective function is expressed as follows:


$$\begin{array}{c} min\;of=\min\{w_{1}\left(1-DG-H\overset{k}{\underset{s}{C}}\right)+w_{2}\left(1-EV-H\overset{k}{\underset{s}{C}}\right)++w_{3}(D\overset{k}{\underset{s}{I}}/DI_{BC})+w_{4}(PL\overset{k}{\underset{s}{I}})\},\;\forall \;s,\;\forall \;k \end{array}$$ (25)

subject to:


$$\begin{array}{c} V_{LL}<\left|\overset{k}{\underset{s,i}{V}}\right|<V_{UL},\;\forall \;i,\;\forall \;s,\;\forall \;k \end{array}$$ (26)

$$\begin{array}{c} \left|\overset{K}{\underset{s,L}{I}}\right|<I_{UL},\;\forall \;L,\;\forall \;s,\;\forall \;K \end{array}$$ (27)

$$\begin{array}{c} \overset{k}{\underset{loss_{s}}{P}}<\overset{BC}{\underset{loss}{P}},\;\forall \;s,\;\forall \;K \end{array}$$ (28)

$$\begin{array}{c} 0\leq P_{WT}\leq \overset{UL}{\underset{WT}{P}} \end{array}$$ (29)

$$\begin{array}{c} 0\leq P_{PV}\leq \overset{UL}{\underset{PV}{P}} \end{array}$$ (30)

$$\begin{array}{c} \overset{k}{\underset{EV_{s,r}}{E}}\leq \overset{rated}{\underset{EV,r}{E}},\forall \;r,\;\forall \;s,\;\forall \;k \end{array}$$ (31)

Eq (26) imposes upper and lower voltage limits at each bus to ensure voltage profiles remain within permissible bounds across all scenarios and throughout the day.

Eq (27) constrains feeder currents to stay within the thermal ampacity, *I*_*UL*_, of the conductors at every hour, while Eq (28) limits the total active power losses to values not exceeding those of the base case.

Eqs (29) and (30) restrict the output of each DG unit to remain below its rated capacity. Then, the EV charging demand in any scenario and at any hour cannot exceed the physical rated capacity of the EV station.

### 4.2 Smart inverter modeling

Conventional PV inverters primarily convert direct current (DC) from PV modules into alternating current (AC) for grid connection. However, with the increasing penetration of distributed PV systems, modern smart inverters (SIs) have evolved to provide additional grid-support functionalities beyond simple power conversion. A key concern in PV integration is overvoltage, which can compromise voltage stability and power quality. To mitigate such issues, SIs employ several control modes, including Volt/Var, Volt/Watt, Frequency/Watt, and fixed power factor control. Among these, Volt/VAR control is the most prevalent due to its high effectiveness in maintaining voltage regulation across DSs [36].

The Volt/VAR function allows the SI to autonomously adjust its reactive power output in response to voltage variations at the point of common coupling (PCC). The control curve is defined by four characteristic voltage points: $V_{1}$, $V_{2}$, $V_{3}$ and $V_{4}$. When the PCC voltage is below $V_{1}$, the SI injects maximum reactive power to raise the voltage. Between $V_{1}$ and $V_{2}$, reactive power injection decreases linearly with slope $m_{1}$. A dead-band region between $V_{2}$ and $V_{3}$ represents the nominal operating range where no reactive power action is needed. As the voltage increases between $V_{3}$ and $V_{4}$, the SI begins to absorb reactive power following the slope $m_{2}$, reaching its maximum absorption at or beyond $V_{4}$. In this work, the slopes ($m_{1}$, $m_{2}$) and dead-band limits ($V_{2}$, $V_{3}$) are optimized to derive the most effective Volt/VAR control curve [23]. To ensure electrical feasibility, this work adopts an explicit, capability-consistent formulation of the four-segment Volt/VAR characteristic, with constraints guaranteeing monotonicity, correct ordering of curve points, and compliance with inverter apparent-power limits. Each inverter j uses a fixed low-voltage saturation point $V_{1}=0.91\text{\textbackslash{},pu}$ and high-voltage saturation point $V_{4}=1.04\text{\textbackslash{},pu}$. Two decision variables define the slopes in the lower and upper regions:


$$\begin{array}{c} m_{1}\leq 0\;,\;m_{2}\leq 0\; \end{array}$$ (32)

The intermediate breakpoints $V_{2j}$ and $V_{3j}$ are not free variables; they are deterministically computed from the slopes:


$$\begin{array}{c} V_{2_{j}}=\frac{-1}{m_{1_{j}}}+V_{1} \end{array}$$ (33)

$$\begin{array}{c} V_{3_{j}}=\frac{1}{m_{2_{j}}}+V_{4} \end{array}$$ (34)

These expressions guarantee that the deadband width adjusts consistently with the selected slopes. Also, ordering and monotonicity are enforced through the explicit constraints implemented via penalty terms in the optimization to prevent infeasible curve shapes.


$$\begin{array}{c} V_{1}<V_{2_{j}}<V_{3_{j}}<V_{4} \end{array}$$ (35)

The per-unit reactive power as a function of the predefined voltage at the PCC $q_{j}(v)$ is defined through a continuous piecewise-affine Volt/VAR characteristic:


$$\begin{array}{c} q_{j}\left(v\right)=\left\{ \begin{array}{c} 1\;v\leq V_{1}\\ m_{1_{j}}\left(v-V_{1}\right)+1\;V_{1}<v<V_{2_{j}}\;\\ 0\;V_{2_{j}}<v<V_{3_{j}}\\ m_{2_{j}}\left(v-V_{4}\right)-1\;V_{3_{j}}<v<\;V_{4}\;\\ -1\;v\geq V_{4} \end{array}\right. \end{array}$$ (36)

The actual value of reactive-power output is then:


$$\begin{array}{c} {Q_{j}}^{inv}\left(v\right)=q_{j}\left(v\right)\overset{rated}{\underset{j}{Q}} \end{array}$$ (37)

To ensure electrical feasibility, inverter reactive-power operation is coupled to the available apparent-power capacity:


$$\begin{array}{c} (\overset{DG}{\underset{j}{P}}{)}^{2}+({Q_{j}}^{inv}{)}^{2}\leq (\overset{DG}{\underset{rated,j}{S}}{)}^{2} \end{array}$$ (38)

## 5 Case study description and results discussion

Numerous simulations were carried out in MATLAB R2021a on a personal computer equipped with an Intel Core i7 processor (3.0 GHz) and 8 GB of RAM to assess the effectiveness of the proposed optimization framework. The study involved two test systems, with data given in the S1 File, in addition to case studies, followed by an extensive numerical evaluation and in-depth discussion of the obtained results. Furthermore, the employed optimization algorithm (SFOA) was systematically benchmarked against other optimization methods to validate its performance and robustness. The following subsections present detailed descriptions of the test systems, simulation scenarios, numerical analyses, and comparative evaluations.

### 5.1 Test systems

**Fig 4:**
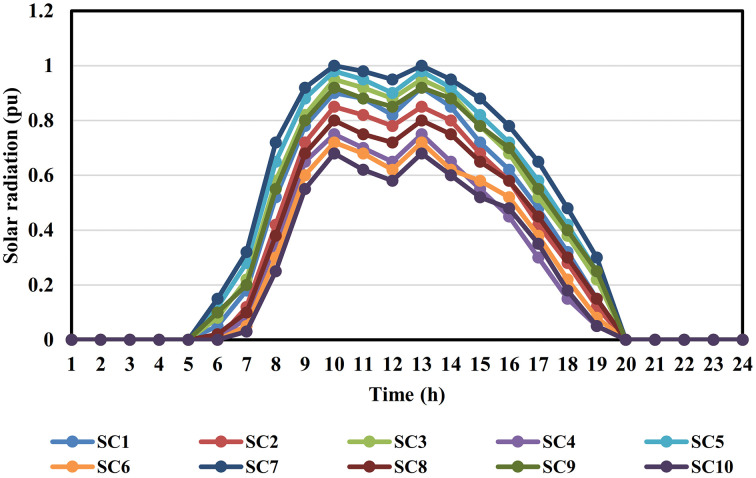
Representative reduced scenarios for solar irradiance.

**Fig 5:**
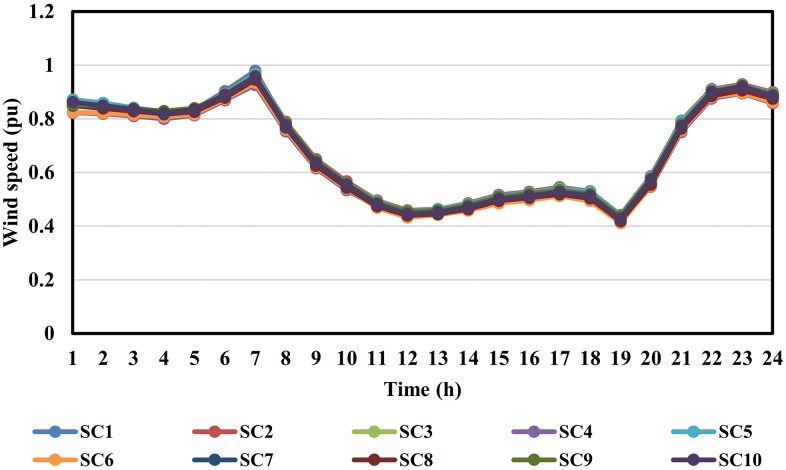
Representative reduced scenarios for wind speed.

**Fig 6:**
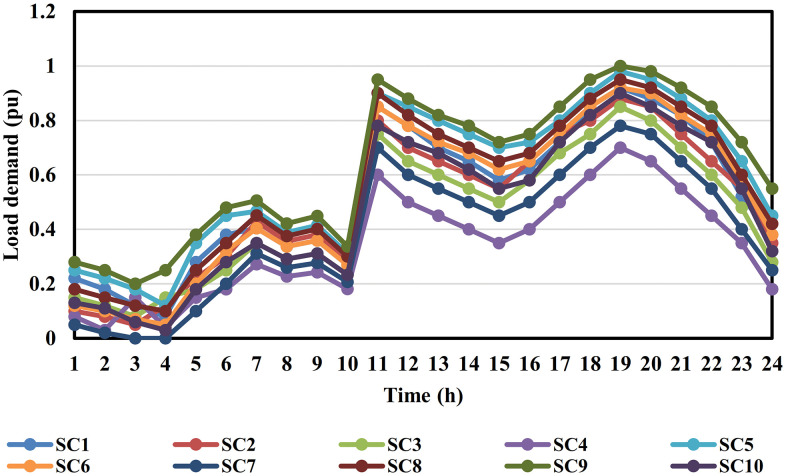
Representative reduced scenarios for load demand.

**Fig 7:**
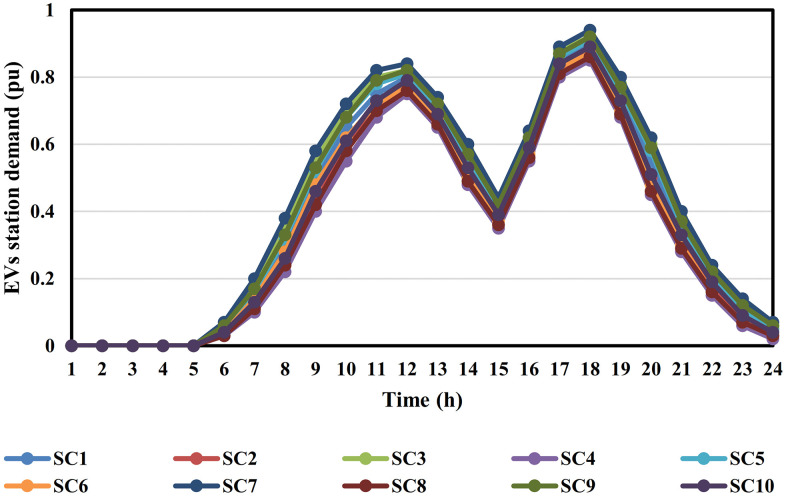
Representative reduced scenarios for EV charging demand.

**Fig 8:**
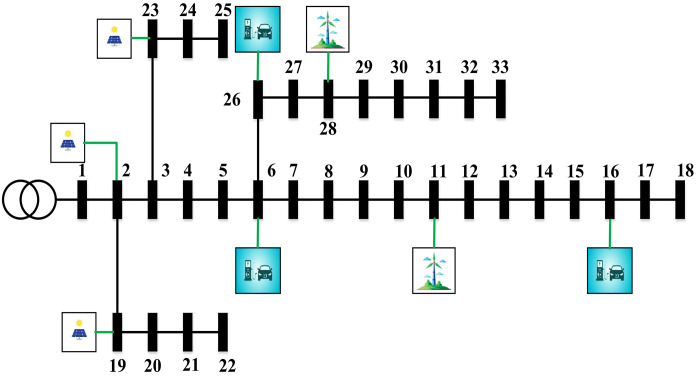
Single-line diagram of the candidate DS IEEE 33-bus.

The effectiveness of the proposed stochastic optimization framework was tested using the IEEE 33-bus benchmark DS, and its scalability was further validated on a real-world 59-bus Egyptian distribution system (EDS) located in Cairo. Figs 4–7 present the forecasted profiles of load demand, solar irradiance, wind speed, and EVs charging demand considered in the analysis. To incorporate stochastic behaviour, 1,000 random scenarios were initially produced via Monte Carlo simulation, after which a scenario reduction algorithm was employed to condense them into 10 representative scenarios that preserve the main probabilistic characteristics of the original dataset. The IEEE 33-bus DS [37], is utilized as a benchmark to evaluate the effectiveness of the proposed methodology. The network operates on a 12.66 kV base voltage and 10 MVA base apparent power. It experiences a maximum load of 3.715 MW and 2.30 MVAR. Under the base-case scenario—excluding DG units and EVs—the total active power loss is approximately 263 kW. Predetermined locations for wind-based DG units are identified at buses 11 and 28, while PV-based DGs are considered for buses 2, 19, and 23. The predetermined positions for EV charging stations are determined to be buses 6, 16, and 26. The single-line diagram of the IEEE 33-bus DS, depicting the candidate placement of renewable sources and charging stations, is presented in Fig 8.

**Fig 9:**
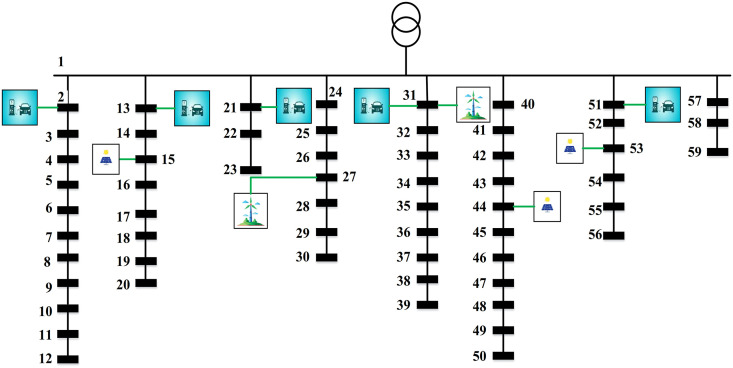
Single-line diagram of the candidate DS Cairo 59 bus.

To further demonstrate the scalability and robustness of the proposed optimization framework, a real-world 59-bus DS located in Cairo, Egypt [38], is also utilized for validation. The system is modeled with a 22 kV base voltage and a 100 MVA base apparent power, supplying a total peak demand of 50.348 MW and 21.448 MVAR. In the base case configuration, excluding DG units and EVs, the total active power loss is approximately 391 kW. The predetermined deployment sites for the wind-based units are buses 27 and 31, whereas the PV units are to be installed at buses 15, 44, and 53. Furthermore, the EV charging stations are predetermined at buses 2, 13, 21, 31, and 51, selected to ensure balanced system loading and enhanced accessibility within the distribution network. The single-line representation of the Cairo 59-bus DS, indicating the candidate allocation of renewable generators and charging stations, is illustrated in Fig 9.

The designated locations of the DG units and EV charging stations were established after conducting multiple integration trials that allowed their placement to vary freely across all load buses, from bus 2 to the terminal node. The final configuration demonstrated superior technical performance and higher hosting capacity compared with alternative placements. These outcomes confirm that the adopted allocation effectively enhances network operation while maintaining practical feasibility.

### 5.2 Results and discussions

This research presents an innovative stochastic and multi-objective optimization framework with time-dependent characteristics, developed to enhance the combined HC of both DG units and EVs. The framework concurrently seeks to reduce system active power losses and voltage deviation indices. The simultaneous improvement in DG and EV hosting capacities is accomplished through a synergistic management strategy that coordinates both active and reactive powers. Specifically, the management of active power is handled by regulating EV charging behavior, while reactive power optimization is achieved through the strategic operation of SI units. By promoting the large-scale integration of renewable energy sources and EVs, this work contributes to advancing power system operation sustainability and supports global clean energy transition objectives. The proposed methodology is examined under four different cases to comprehensively assess its effectiveness.

- **Case 1:** Evaluation of the optimal DG-HC without considering EV aggregators—only DG units are included, and the influence of EVs is neglected. All PV inverters are assumed to operate as conventional (non-smart) inverters.
- **Case 2:** Assessment of the optimal DG-HC considering the presence of EV aggregators with uncoordinated charging behavior, while SIs functionalities are still disregarded (PV inverters remain conventional).
- **Case 3:** Evaluation of the optimal combined DG-HC and EV-HC, incorporating coordinated EV charging management, but still neglecting SI control.
- **Case 4:** Evaluation of the optimal combined DG-HC and EV-HC, enhanced by both coordinated EV charging and the optimal Volt/VAR control of SIs.

The present study explores the power of properly selecting the multi-objective weighting factors to enhance the combined DG-HC and EV-HC while concurrently reducing DS power losses and voltage deviations. In alignment with the conclusions in [16,39] the simulation outcomes indicate that higher levels of DG penetration or intensified EV charging demand typically result in elevated network losses. Such findings emphasize the importance of incorporating a power-loss-related objective into the optimization process to ensure that any HC improvement remains technically feasible and energy-efficient. To assess the robustness of the multi-objective formulation and justify the adopted weighting coefficients, an extensive sensitivity analysis was conducted across eight distinct weight combinations (Table 2) at hour 13 under scenario 5. These combinations were designed to represent diverse optimization priorities, including hosting-biased, DG-dominated, EV-dominated, and linearly increasing/decreasing patterns. The results demonstrate that combinations emphasizing only DG/EV hosting (e.g., 0.5–0.5–0–0 and 0.4–0.4–0.1–0.1) consistently violate the power-loss constraint, indicating that overemphasizing capacity expansion leads to operational infeasibility. Similarly, DG- and EV-priority cases yield high power losses or voltage deviations. While the linearly increasing weights (0.1–0.2–0.3–0.4) satisfy all constraints, they significantly reduce both DG and EV hosting capacity due to over-penalization of the reliability objective. In contrast, the proposed weights (0.3, 0.3, 0.1, 0.3) achieve the best balance between technical feasibility and hosting-capacity enhancement while maintaining voltage profiles within limits and reducing power losses to 203.696 kW. This configuration appropriately emphasizes DG and EV hosting while ensuring sufficient attention to voltage performance and reliability.

**Table 2:** Sensitivity analysis of weighting-coefficient combinations and their impact on the optimization results.

Combination number	Weighting factors				OF	DG installed capacity (MW)	EV charging demand (MWh)	Vmin (pu)	Vmax (pu)	Ploss (kW)	Constraints violation
	$w_{1}$	$w_{2}$	$w_{3}$	$w_{4}$							
Hosting-biased	0.5	0.5	0	0	0.4396	15.53	5.23	0.9519	1.0037	265.1813	Ploss violation
Hosting-biased, with little attention to other objectives	0.4	0.4	0.1	0.1	0.4779	15.83	5.12	0.9639	1.0049	265.1569	Ploss violation
Linearly decreasing weights	0.4	0.3	0.2	0.1	0.4789	16.624	4.8685	0.9758	1.0083	265.4637	Ploss violation
Linearly increasing weights	0.1	0.2	0.3	0.4	0.4490	7.2698	2.0271	0.9866	1.0028	129.8056	All are acceptable
DG priority	0.6	0.2	0.1	0.1	0.5855	18.89	4.4920	0.9797	1.0062	264.8514	Ploss violation
EV priority	0.2	0.6	0.1	0.1	0.3459	12.088	5.42	0.9541	1.0027	264.7684	Ploss violation
Base	0.35	0.35	0.15	0.15	0.4930	15.47	5.08	0.9684	1.0063	265.0483	Ploss violation
**Proposed**	0.3	0.3	0.1	0.3	0.5669	11.997	4.49	0.9757	1.0028	203.696	All are acceptable

The simulation parameters utilized throughout the analysis are presented in Table 3. Similar to [40]. The Korean standard voltage range [0.91–1.04] p.u. is adopted in this work to maintain realistic operational constraints and ensure accurate HC assessment under varying penetration levels.

**Table 3:** Parameters used in the studied systems.

Parameter	Value (unit)
Maximum number of iterations	300
Number of search agents	50
$w_{1},w_{2},\;w_{3}and\;,\;w_{4}\;$	0.3, 0.3, 0.1 and 0.3
$V_{LL}$and $V_{UL}$	0.91 and 1.04
$I_{c}$ and $I_{std}$	150 and 1000 (W/m^2^)
$\omega_{cut-in}$	4 (m/s)
$\omega_{cut-out}$	25 (m/s)
$\omega_{rated}$	15 (m/s)
$\overset{UL}{\underset{WT}{P}}$ and $\overset{UL}{\underset{PV}{P}}$ (IEEE 33 bus)	12.5 (MW)
$\overset{UL}{\underset{WT}{P}}$ and $\overset{UL}{\underset{PV}{P}}$ (Cairo 59 bus)	35 (MW)
$\overset{rated}{\underset{EV}{E}}$ (IEEE 33 bus)	2 (MW)
$\overset{rated}{\underset{EV}{E}}$ (Cairo 59 bus)	22 (MW)
$\overset{max}{\underset{SI}{Q}}$ (IEEE 33 bus)	5 (MVAR)
$\overset{max}{\underset{SI}{Q}}$ (Cairo 59 bus)	17 (MVAR)

To clearly quantify the influence of EVs charging coordination when integrated with SIs Volt/Var control, the results of the representative scenario (scenario 5) for the four examined cases are presented in Table 4, covering both the IEEE 33-bus and Cairo 59-bus DSs.

**Table 4:** Comparative results of the four proposed case studies under scenario 5 for both DSs.

Test system	Case study	Average power loss (kW)	Average installed capacity (MW)	Average EVs charging energy (MWh)	Minimum recorded voltage (pu)	Maximum recorded voltage (pu)	Average DI (%)
IEEE 33-bus system	Case 1	91.2	3.8	–	0.971	1.0246	19.6
	Case 2	119.5	5.5	1.18	0.9108	1.0075	50.3
	Case 3	149.3	6.99	3.2	0.9493	1.0094	46.8
	Case 4	173.3	8.65	4.59	0.9719	1.0052	29.8
Cairo 59-bus system	Case 1	175.9	17.51	–	0.9871	1.0033	12.41
	Case 2	205.17	34.63	23.6	0.9871	1.0018	11.65
	Case 3	251.4	41.7	83.4	0.9871	1.0013	12.6
	Case 4	248.6	49.55	89.1	0.9896	1.0014	11.3

In case 1, where EVs were not considered, the IEEE 33-bus system achieved an average DG installed capacity of 3.8 MW, accompanied by 91.2 kW of total active power losses and a 19.6% voltage deviation index. In contrast, the Cairo 59-bus network—characterized by higher loading and a larger number of buses—exhibited an average DG installed capacity of 17.51 MW, total losses of 175.9 kW, and 12.41% VDI. Notably, in the IEEE system, the maximum bus voltage throughout the day approached the upper permissible limit, yet the well-defined operational constraints within the optimization model successfully prevented any violations. It should be emphasized that incorporating EV behavior is essential for accurate estimation of DG-HC. As observed in case 2, where uncoordinated EV charging was introduced, the mean DG installed capacity increased considerably, by approximately 44.7% in the IEEE 33-bus system and 97.7% in the Cairo 59-bus system, relative to case 1. However, this uncoordinated integration deteriorated the voltage profile, as reflected by a higher voltage deviation index value in the IEEE 33-bus system. Although cases 3 and 4 yielded higher DG-HC and EV-HC levels, these improvements were accompanied by increased active power losses. Nevertheless, the proposed optimization framework effectively mitigated this rise, maintaining total losses below the base-case level.

The advantages of coordinated EV charging become evident in case 3, where mean values of DG installed capacity and EVs charging level improved by 27% and 171%, respectively, in the IEEE 33-bus system, and by 20.4% and 253.3% in the Cairo 59-bus system compared to case 2. Moreover, EV charging coordination significantly enhanced the voltage performance in the IEEE network, reducing the voltage deviation index from 50.3% to 46.8%.

The effectiveness of the proposed synergistic enhancement approach, which integrates EV charging coordination with SI Volt/Var control, is further validated in case 4. This approach achieved substantial gains—57.3% in DG installed capacity and 288.9% in EVs charging level for the IEEE 33-bus system, and 43.1% and 277.5%, respectively, for the Cairo 59-bus network compared to case 2. Both systems exhibited voltage magnitudes closely centered on the nominal 1.0 p.u., with the lowest voltage deviation index values among all cases. These findings underscore the capability of the proposed combined strategy to simultaneously maximize DG and EV hosting capacities, enhance voltage profile, and reduce overall system losses.

The superiority of the proposed stochastic framework over traditional deterministic techniques in evaluating the combined DG–EV-HC is clearly demonstrated in Figs 10–23. Specifically, Figs 10–17 illustrate the hourly DG-HC obtained for each representative scenario across the candidate case studies for the IEEE 33-bus and Cairo 59-bus systems, respectively. Similarly, Figs 18–23 depict the hourly EV-HC under the same scenarios and case studies.

**Fig 10:**
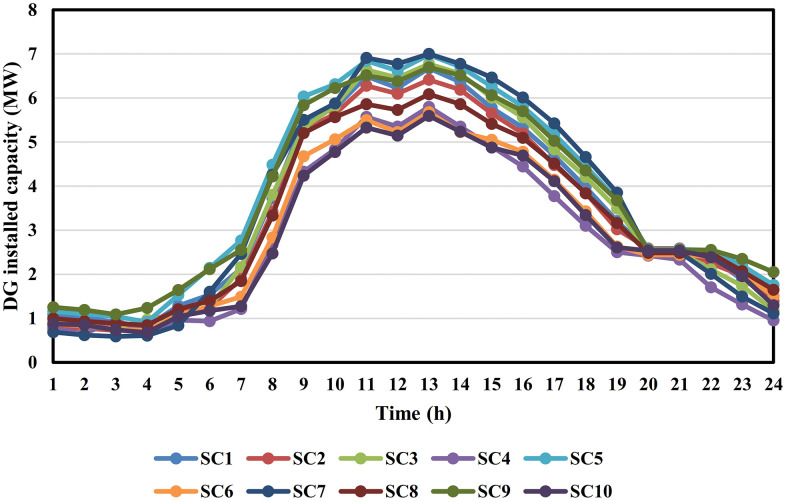
Comparative results of the optimal DG installed capacity derived from the stochastic framework across the four IEEE 33-bus case studies: Case 1.

**Fig 11:**
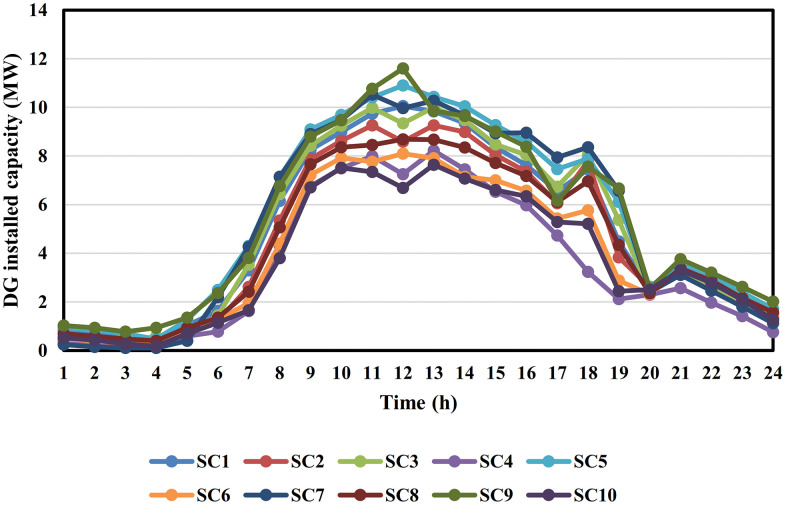
Comparative results of the optimal DG installed capacity derived from the stochastic framework across the four IEEE 33-bus case studies: Case 2.

**Fig 12:**
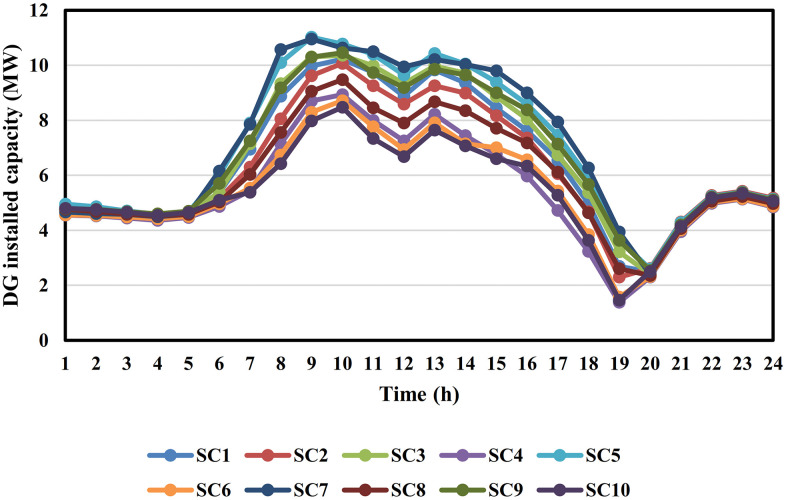
Comparative results of the optimal DG installed capacity derived from the stochastic framework across the four IEEE 33-bus case studies: Case 3.

**Fig 13:**
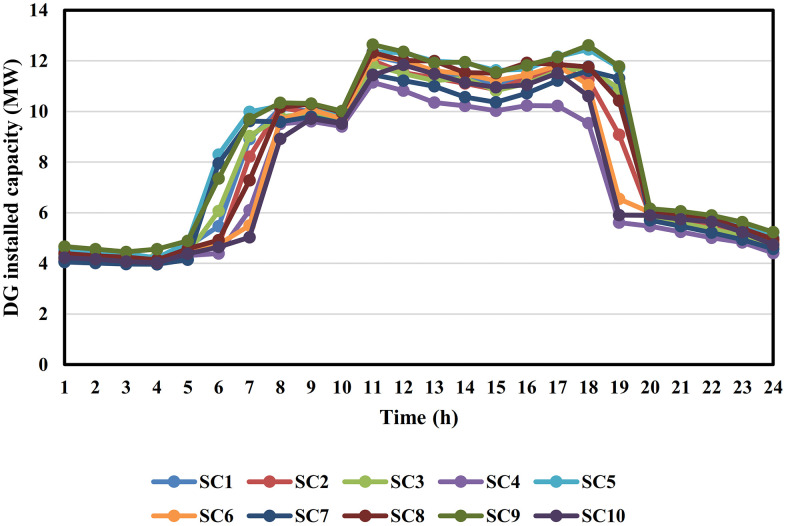
Comparative results of the optimal DG installed capacity derived from the stochastic framework across the four IEEE 33-bus case studies: Case 4.

**Fig 14:**
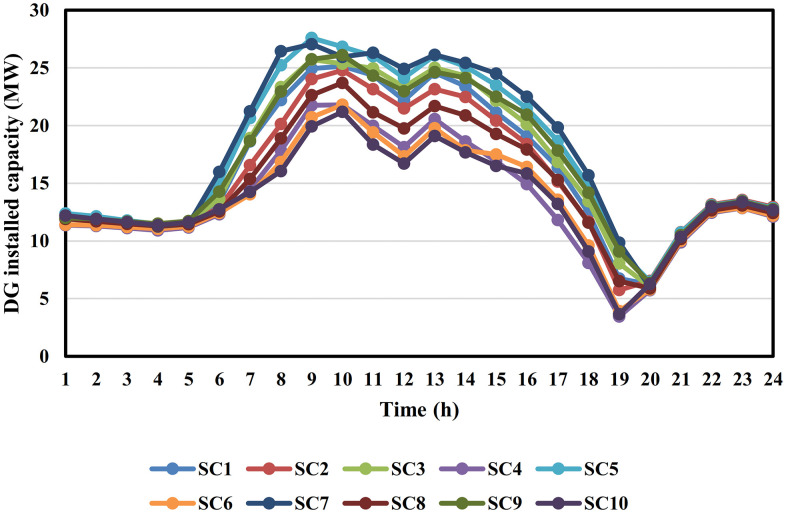
Comparative results of the optimal DG installed capacity derived from the stochastic framework across the four Cairo 59-bus case studies: Case 1.

**Fig 15:**
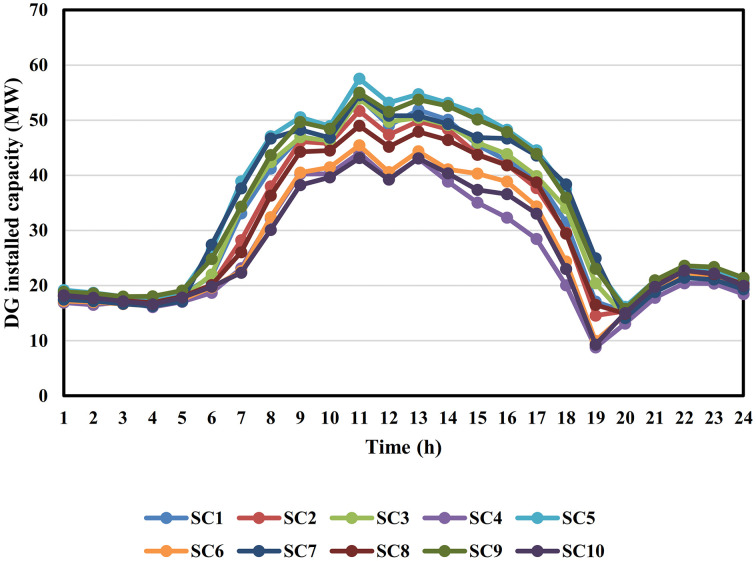
Comparative results of the optimal DG installed capacity derived from the stochastic framework across the four Cairo 59-bus case studies: Case 2.

**Fig 16:**
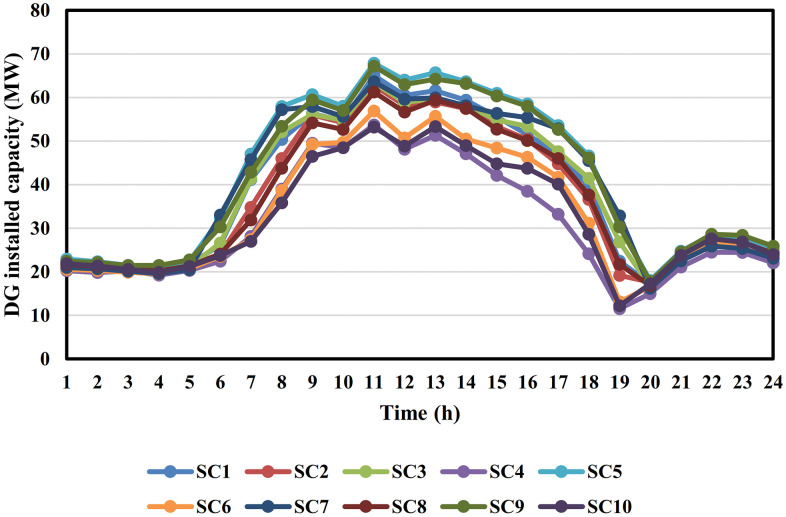
Comparative results of the optimal DG installed capacity derived from the stochastic framework across the four Cairo 59-bus case studies: Case 3.

**Fig 17:**
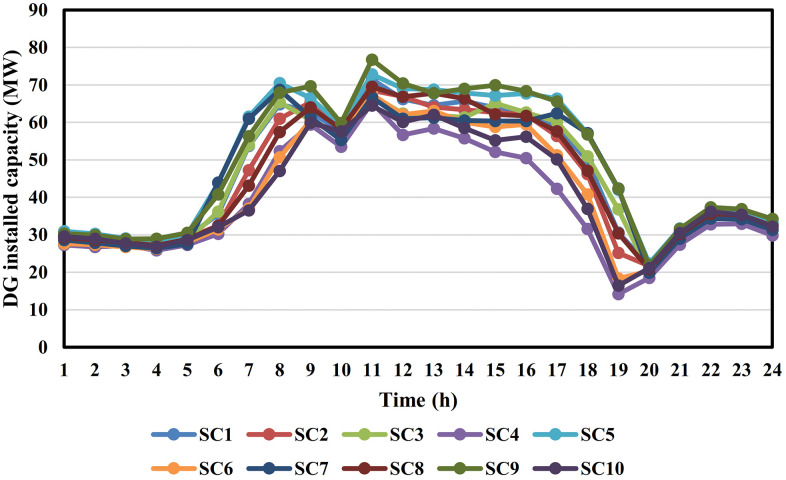
Comparative results of the optimal DG installed capacity derived from the stochastic framework across the four Cairo 59-bus case studies: Case 4.

**Fig 18:**
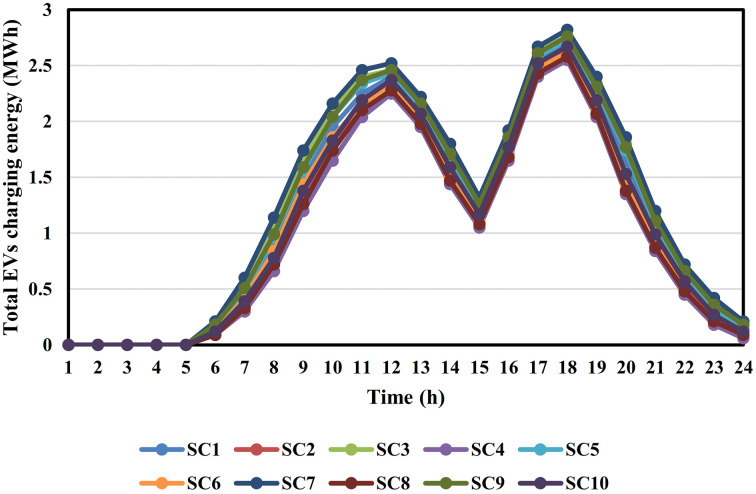
Comparative results of the optimal EV stations consumption energy derived from the stochastic framework across the three IEEE 33-bus case studies: Case 2.

**Fig 19:**
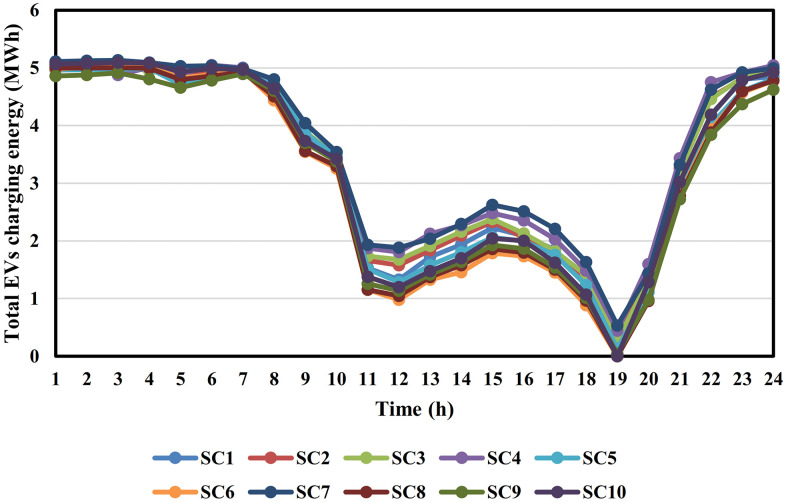
Comparative results of the optimal EV stations consumption energy derived from the stochastic framework across the three IEEE 33-bus case studies: Case 3.

**Fig 20:**
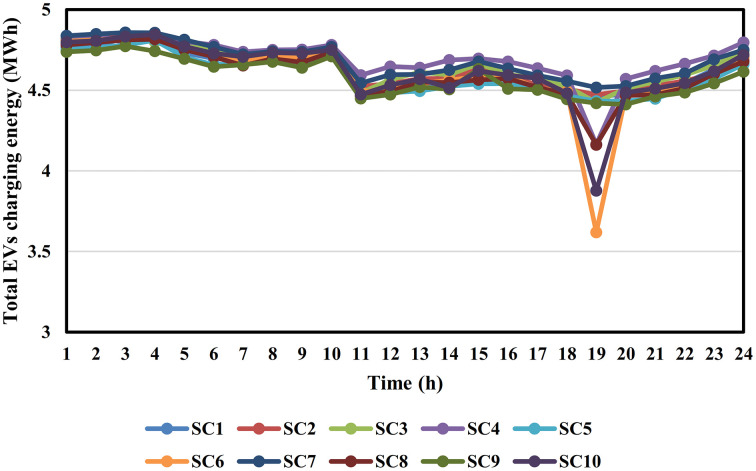
Comparative results of the optimal EV stations consumption energy derived from the stochastic framework across the three IEEE 33-bus case studies: Case 4.

**Fig 21:**
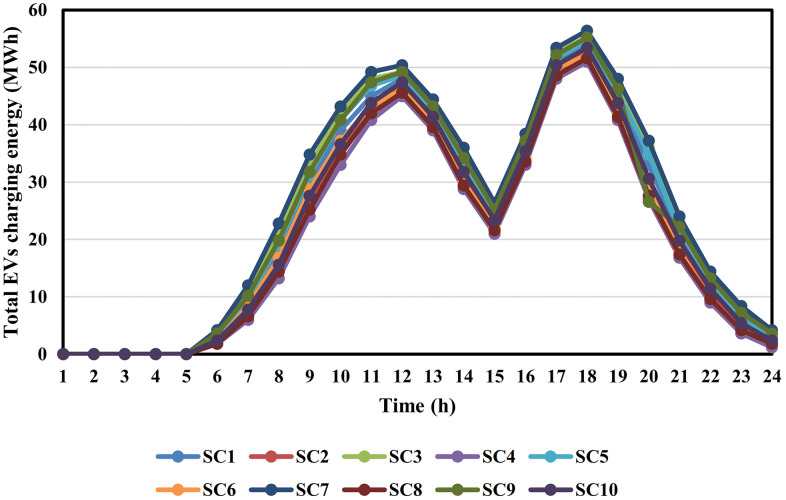
Comparative results of the optimal EVs stations consumption energy derived from the stochastic framework across the three Cairo 59-bus case studies: Case 2.

**Fig 22:**
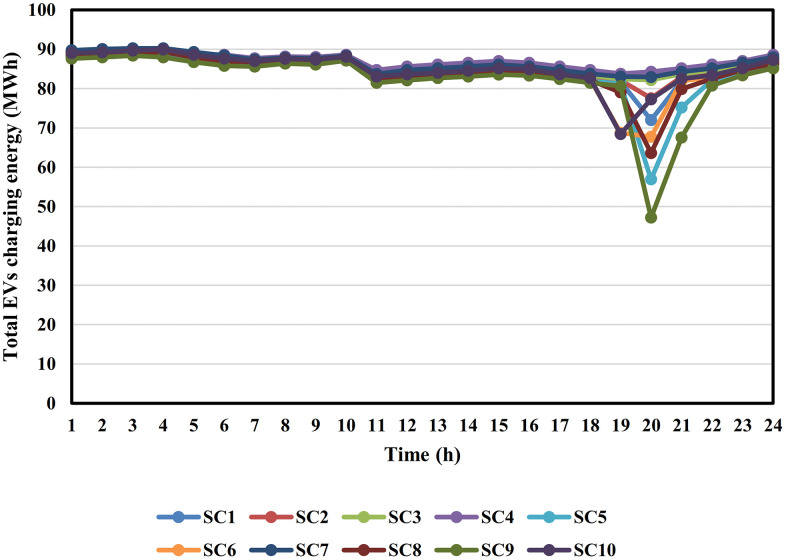
Comparative results of the optimal EVs stations consumption energy derived from the stochastic framework across the three Cairo 59-bus case studies: Case 3.

**Fig 23:**
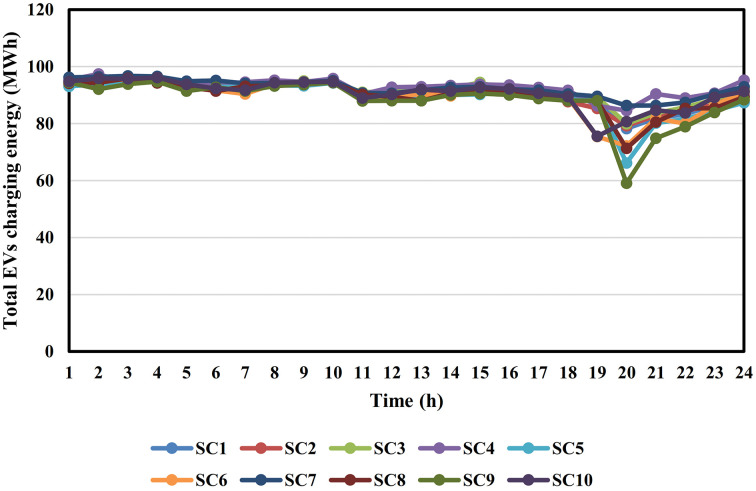
Comparative results of the optimal EVs stations consumption energy derived from the stochastic framework across the three Cairo 59-bus case studies: Case 4.

These results confirm that the stochastic approach offers a more comprehensive and realistic assessment of HC than deterministic methods. While deterministic analysis produces a single, highly conservative value based on extreme conditions, the stochastic approach captures the full spectrum of probable operating scenarios, presenting both DG-HC and EV-HC as a dynamic range rather than a fixed quantity. This enhances both the accuracy and adaptability of system planning, supporting greater renewable energy integration while maintaining grid reliability and stability.

Under the coordination framework of case 4, the stochastic results for the Cairo 59-bus system (Figs 18 and 23) reveal that the maximum DG installed capacity reached approximately 76.7 MW at hour 11 in scenario 9, while the peak EVs stations consumed energy attained 97.3 MWh at hour 2 in scenario 4 Conversely, the minimum DG installed capacity was recorded at 14.2 MW during hour 19 in scenario 4, and the lowest EVs stations consumed energy approached 59MWh at hour 20 in scenario 9. Similarly, for the IEEE 33-bus system under the same synergistic approach (Figs 13 and 20), the highest DG installed capacity was found to be approximately 12.64 MW at hour 11 in scenario 9, while the maximum EVs stations consumed energy reached 4.85MWh at hour 3 in scenario 7. The minimum DG installed capacity occurred at 3.96 MW during hour 4 in scenario 7, and the minimum energy consumption by EV stations was 3.6 MWh at hour 19 in scenario 6.

**Fig 24:**
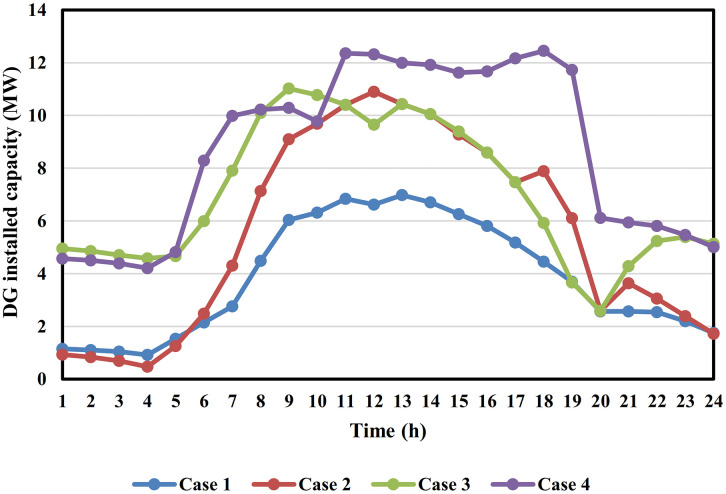
Hourly variation of DG installed capacity through scenario 5 considering case studies: IEEE 33-bus DS.

**Fig 25:**
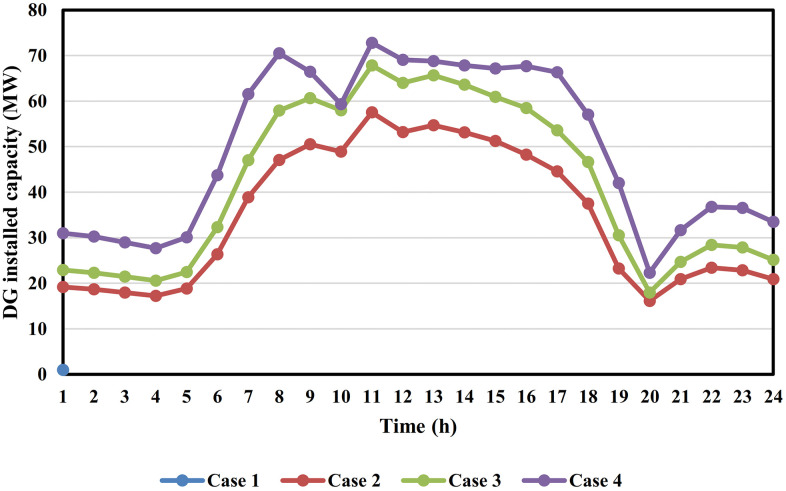
Hourly variation of DG installed capacity through scenario 5 considering case studies: Cairo 59-bus DS.

**Fig 26:**
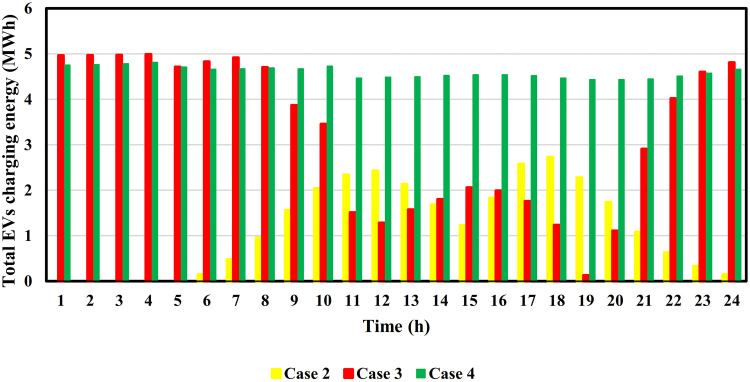
Hourly variation of EVs stations consumption energy through scenario 5 considering case studies: IEEE 33-bus DS.

**Fig 27:**
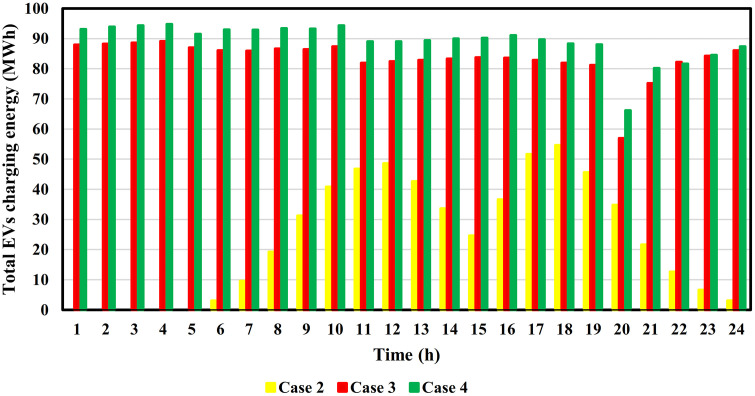
Hourly variation of EVs stations consumption energy through scenario 5 considering case studies: Cairo 59-bus DS.

The effectiveness of the proposed synergistic enhancement framework in improving the CHC is obviously illustrated in Figs 24–27. For the IEEE 33-bus DS under scenario 5, the average DG installed capacity exhibits a consistent improvement across the analyzed cases. Specifically, the average DG installed capacity rises from 5.5 MW in Case 2 to 6.99 MW in Case 3, where EV charging coordination alone was implemented. When the synergistic strategy, integrating EV charging coordination with SI Volt/VAR control, is applied in case 4, the DG installed capacity further increases to approximately 8.65 MW. Similarly, the total EVs charging consumption energy demonstrates a notable upward trend. The introduction of EV charging coordination elevates total EVs charging consumption energy from 1.18 MWh in Case 2 to 3.2 MWh in Case 3. The incorporation of the synergistic Volt/VAR-based coordination mechanism results in a more substantial improvement, with total EVs charging consumption energy reaching nearly 4.6 MWh, highlighting the significant role of coordinated control in maximizing system flexibility and capacity utilization. A comparable enhancement pattern is observed in the Cairo 59-bus DS. The average DG installed capacity increases from 34.6 MW in Case 2 to 41.7 MW in Case 3 after the application of EV charging coordination and further escalates to approximately 49.6 MW under the synergistic approach of Case 4. Concurrently, the total EVs charging consumption energy improves from 23.6 MWh in Case 2 to 83.4 MWh in Case 3, and ultimately reaches 89.1 MWh under the combined approach, confirming the superiority of the proposed synergistic method in enhancing both DG and EV hosting capabilities.

**Fig 28:**
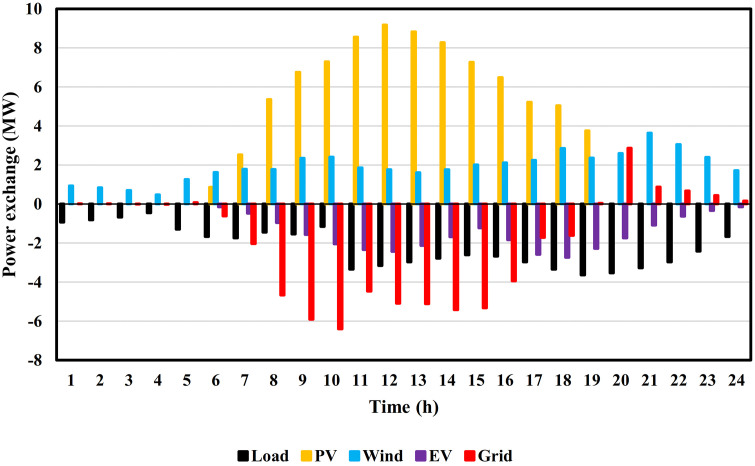
Optimal active power dispatch under scenario 5 in the candidate case studies for IEEE 33-bus DS: Case 2.

**Fig 29:**
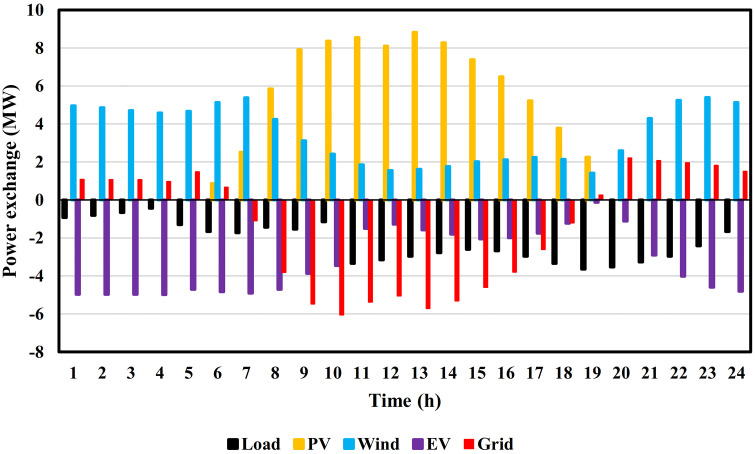
Optimal active power dispatch under scenario 5 in the candidate case studies for IEEE 33-bus DS: Case 3.

**Fig 30:**
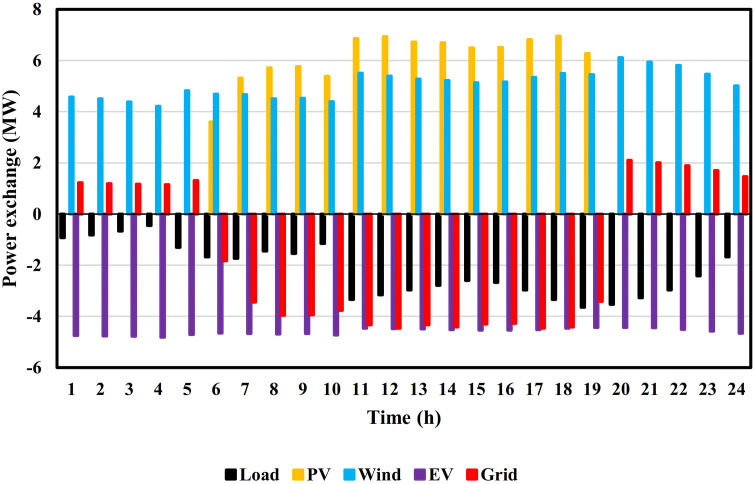
Optimal active power dispatch under scenario 5 in the candidate case studies for IEEE 33-bus DS: Case 4.

**Fig 31:**
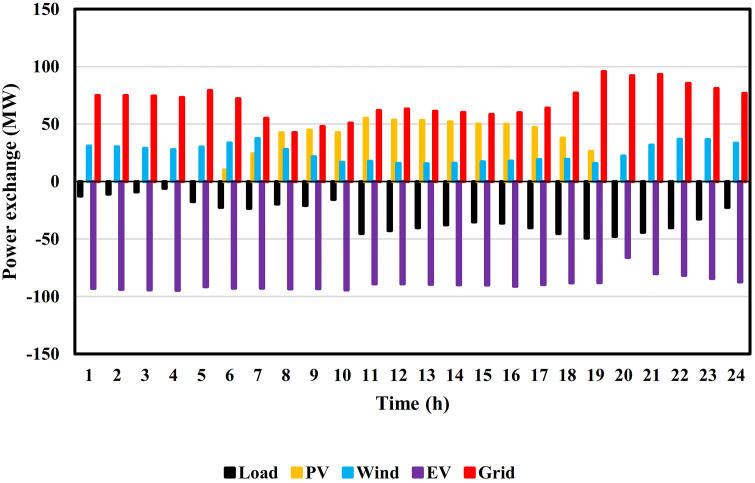
Optimal active power dispatch under scenario 5 in the candidate case studies for Cairo 59-bus DS: Case 2.

**Fig 32:**
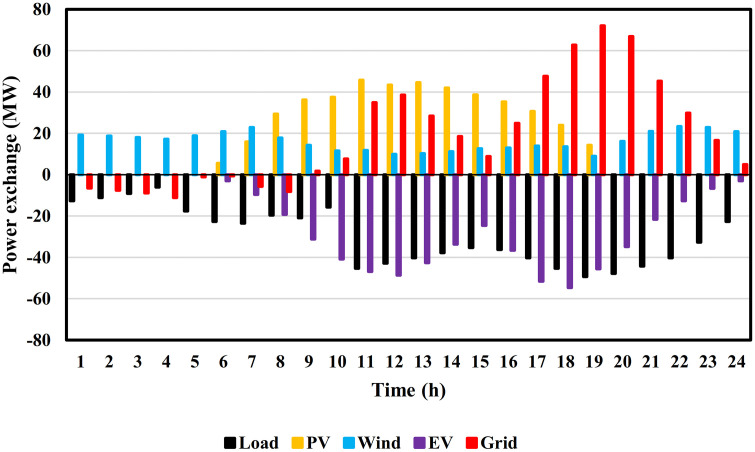
Optimal active power dispatch under scenario 5 in the candidate case studies for Cairo 59-bus DS: Case 3.

**Fig 33:**
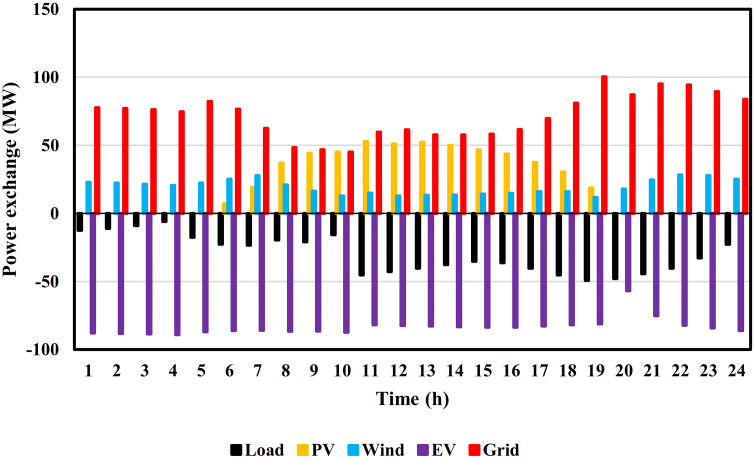
Optimal active power dispatch under scenario 5 in the candidate case studies for Cairo 59-bus DS: Case 4.

The optimal power dispatch profiles obtained for both DSs under scenario 5 are presented in Figs 28–33. The results clearly indicate a significant reduction in grid-supplied power, primarily from fossil-fuel–based generation, following the application of the proposed EV charging coordination strategy in Case 3. This reduction becomes even more pronounced in Case 4, where EV charging coordination is synergistically integrated with SI Volt/VAR control, demonstrating the combined strategy’s effectiveness in minimizing dependency on conventional energy sources.

Furthermore, during periods of high renewable generation and low system demand, surplus power from PV and wind units is exported back to the main grid, contributing to the supply of other distribution networks. This reverse power flow reflects the system’s enhanced capability to accommodate renewable variability and achieve bidirectional energy interaction. The amount of power injected into the grid notably increases in Case 3 and expands in Case 4, confirming the superior performance of the proposed coordinated control scheme in maximizing renewable utilization and improving the grid efficiency.

**Fig 34:**
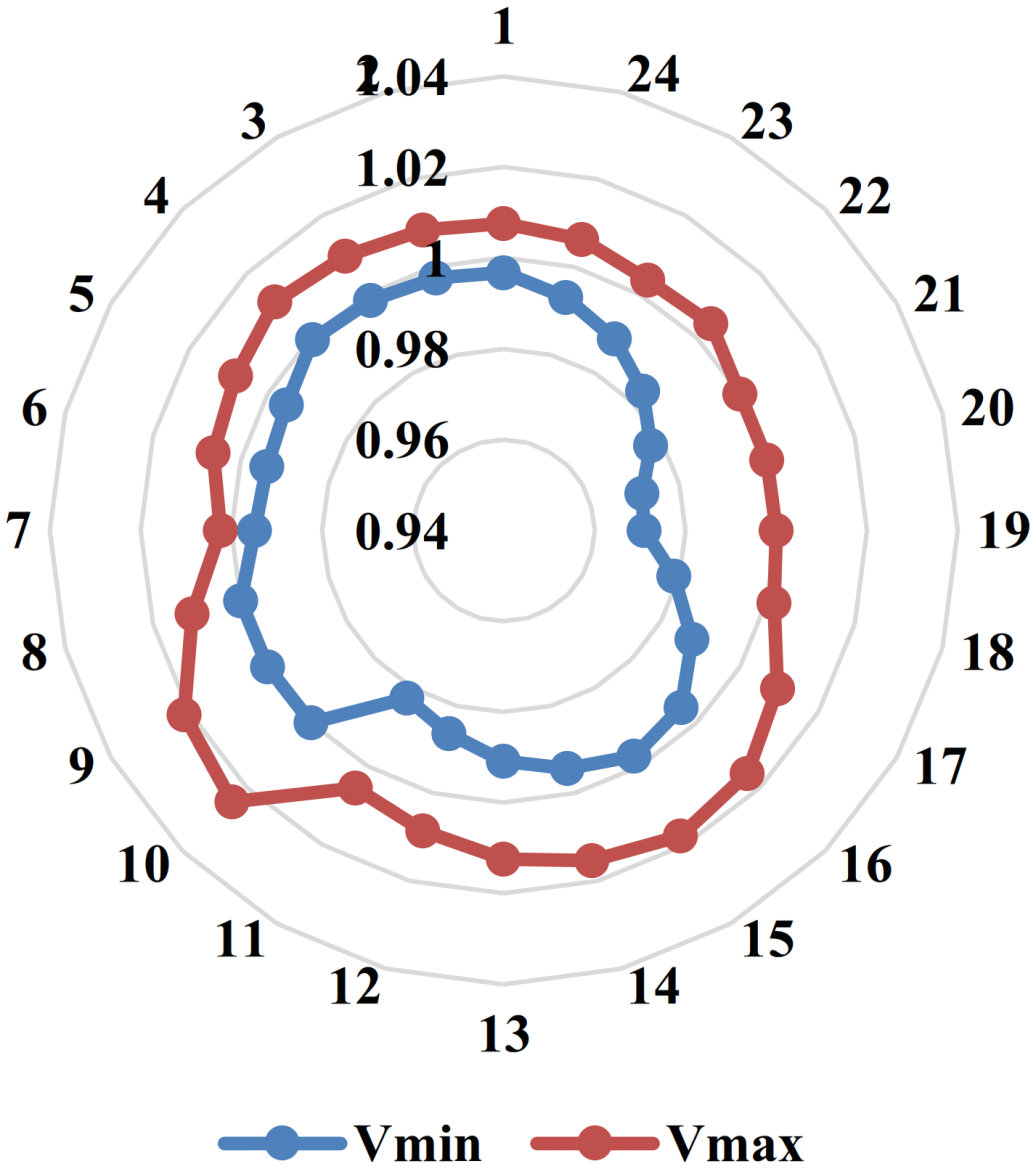
The maximum and minimum recorded bus voltage under scenario 5 through the whole day in IEEE 33-bus DS: Case 1.

**Fig 35:**
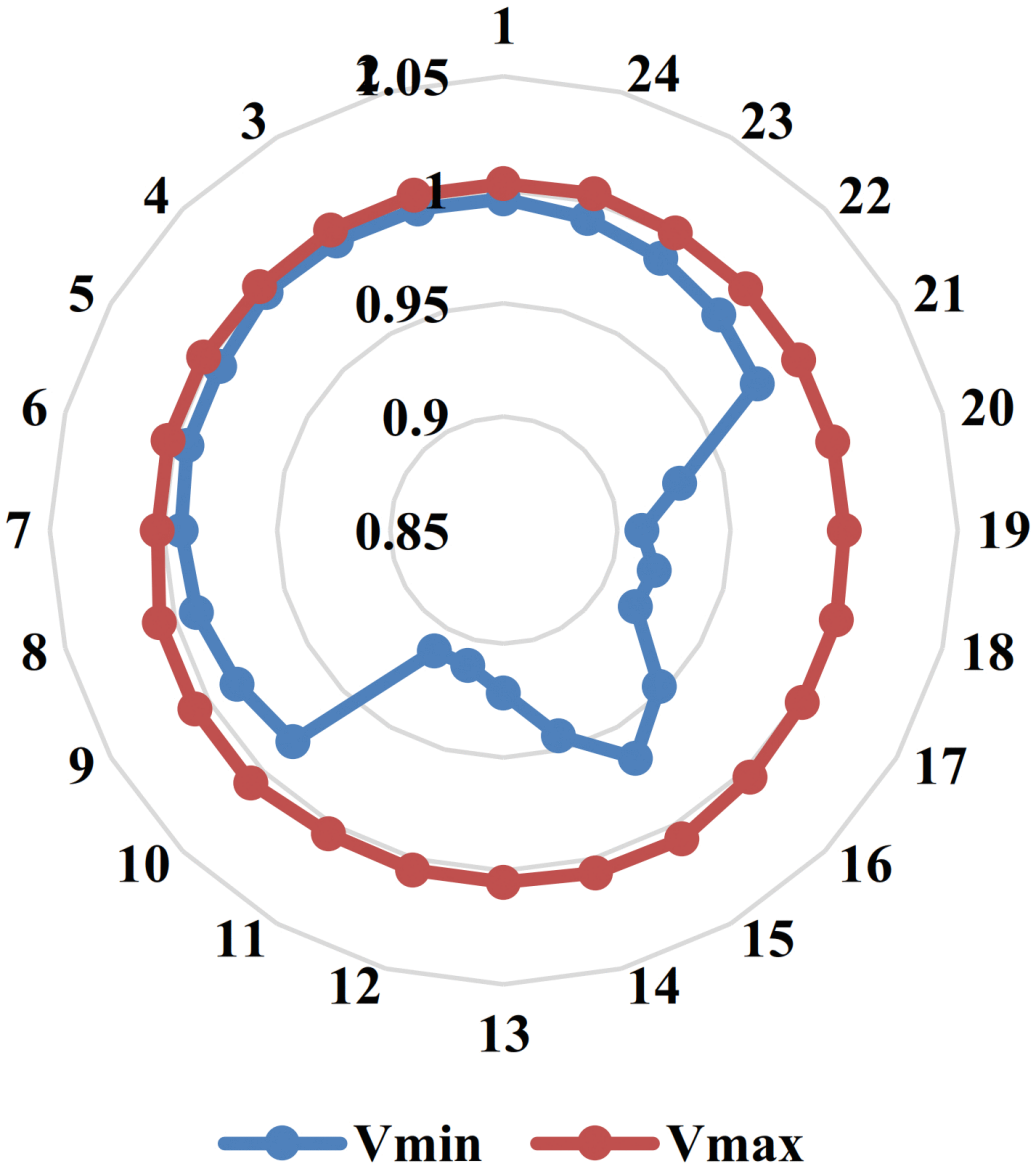
The maximum and minimum recorded bus voltage under scenario 5 through the whole day in IEEE 33-bus DS: Case 2.

**Fig 36:**
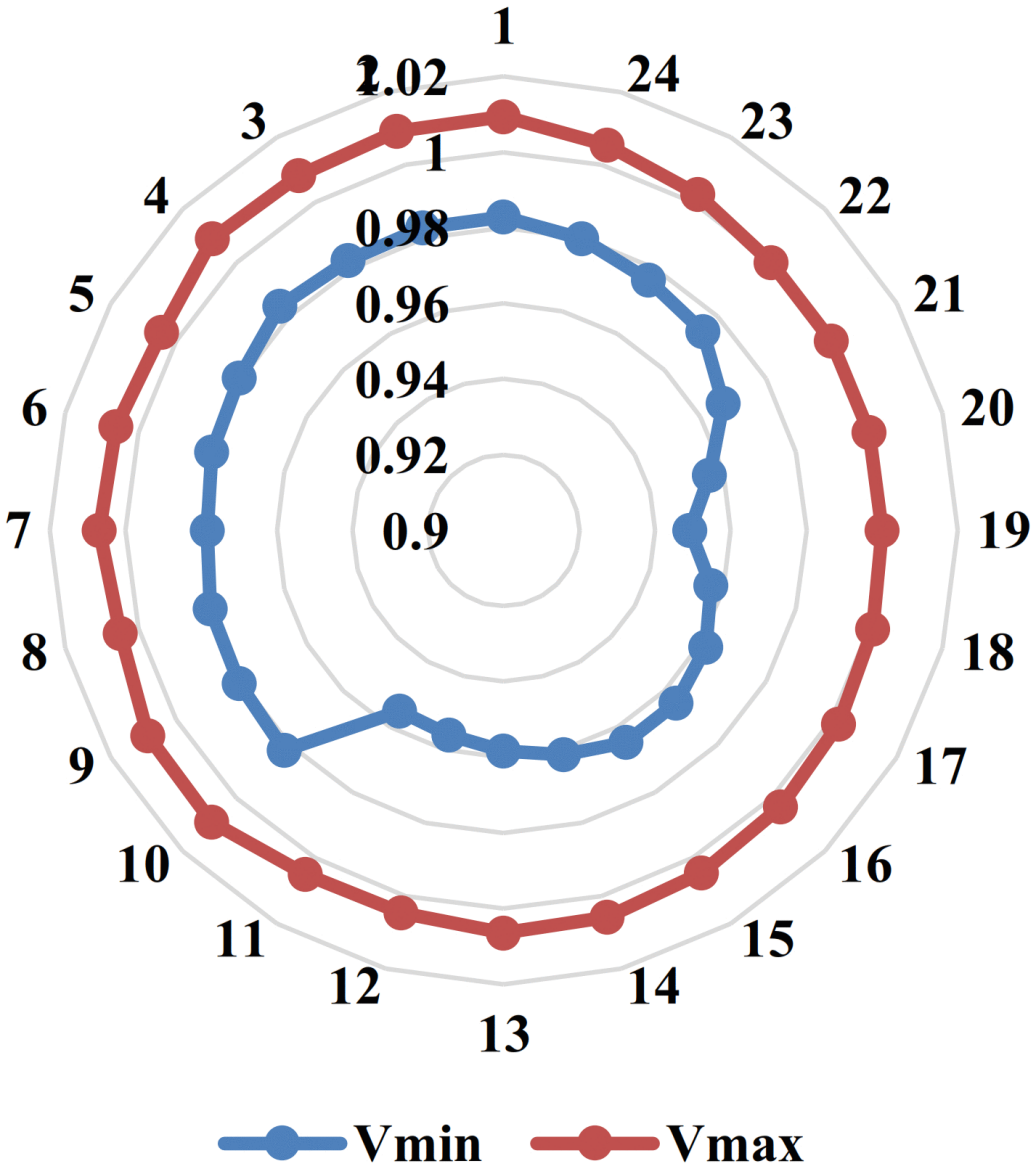
The maximum and minimum recorded bus voltage under scenario 5 through the whole day in IEEE 33-bus DS: Case 3.

**Fig 37:**
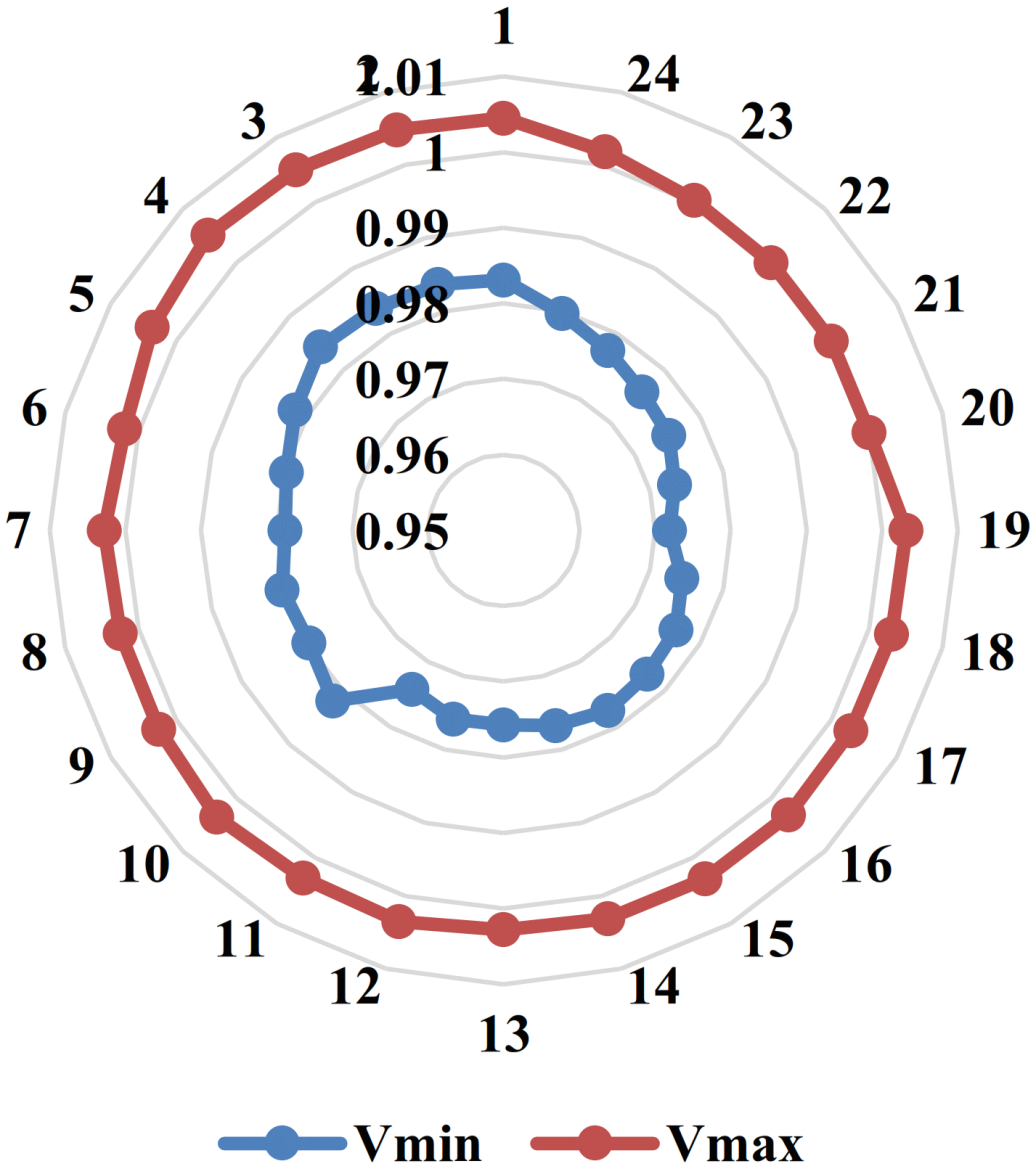
The maximum and minimum recorded bus voltage under scenario 5 through the whole day in IEEE 33-bus DS: Case 4.

**Fig 38:**
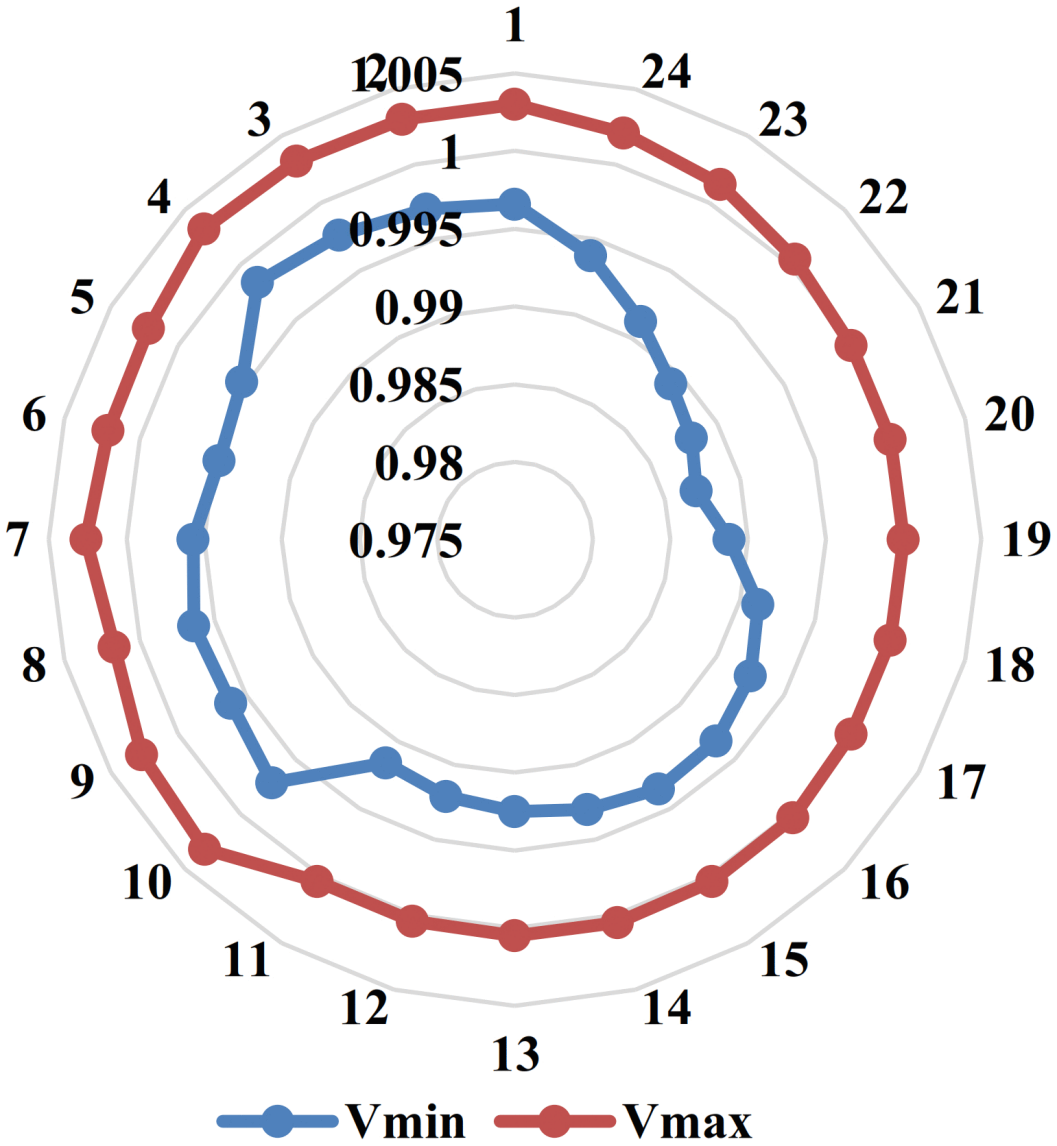
The maximum and minimum recorded bus voltage under scenario 5 through the whole day in Cairo 59-bus DS: Case 1.

**Fig 39:**
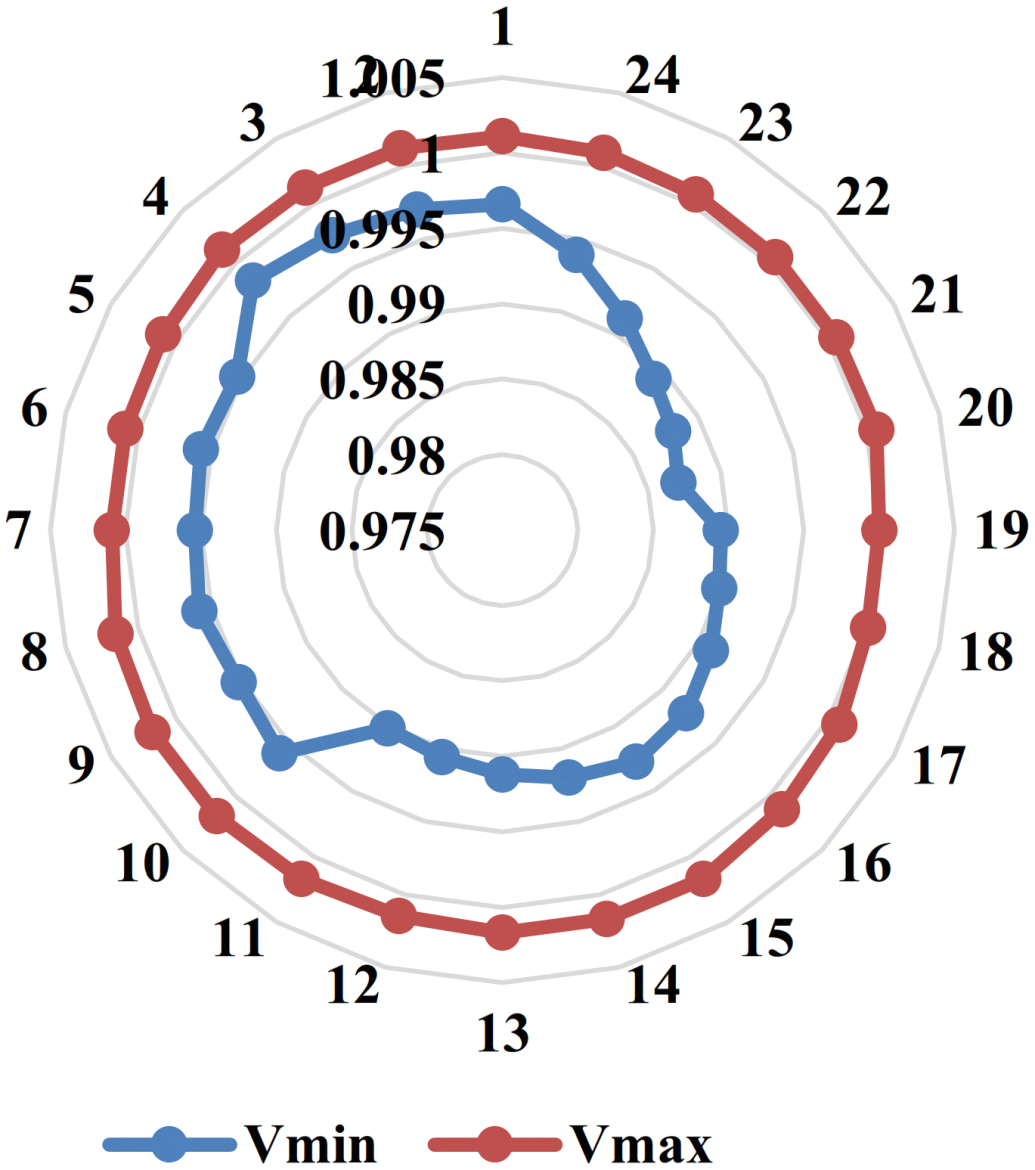
The maximum and minimum recorded bus voltage under scenario 5 through the whole day in Cairo 59-bus DS: Case 2.

**Fig 40:**
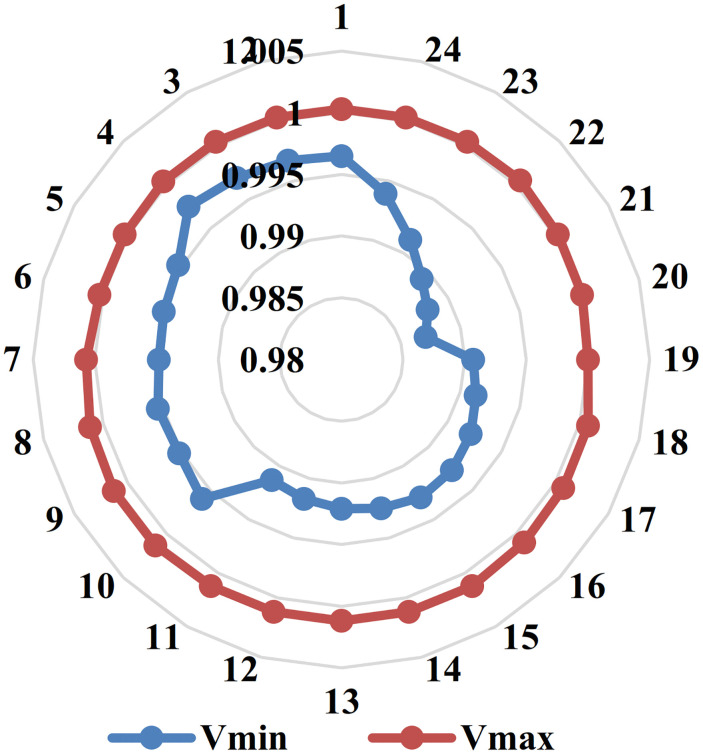
The maximum and minimum recorded bus voltage under scenario 5 through the whole day in Cairo 59-bus DS: Case 3.

**Fig 41:**
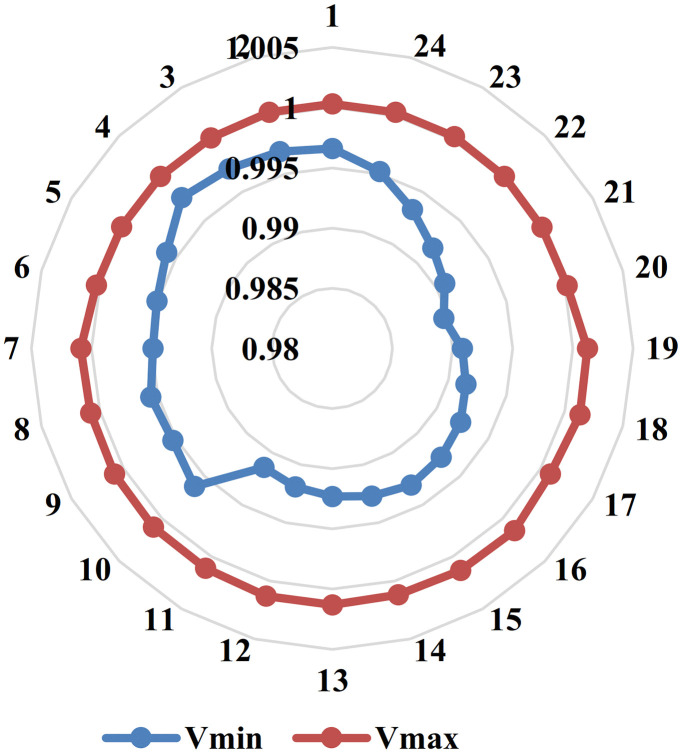
The maximum and minimum recorded bus voltage under scenario 5 through the whole day in Cairo 59-bus DS: Case 4.

The effectiveness of the proposed optimization framework in maintaining voltage across all buses within the allowable range throughout the 24-hour operational horizon is clearly demonstrated in Figs 34–41. Despite the high penetration of renewable energy sources and EV demand, which typically induce voltage fluctuations and potential limit violations, the system voltages remain consistently within the allowable operating range. Furthermore, the proposed synergistic enhancement technique significantly improves the voltage profile and reduces the voltage deviation index, thereby strengthening multi-objective optimization performance and maximizing voltage-constrained CHC.

**Fig 42:**
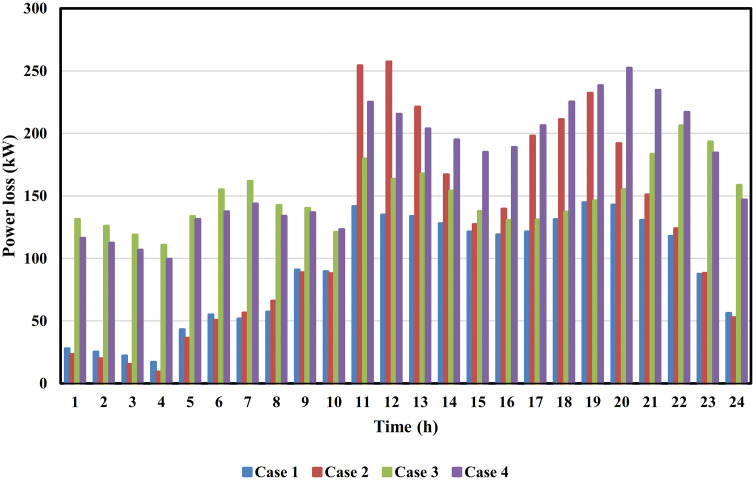
Total active power loss recorded at each hour under scenario 5 for the four case studies: IEEE 33-bus DS.

**Fig 43:**
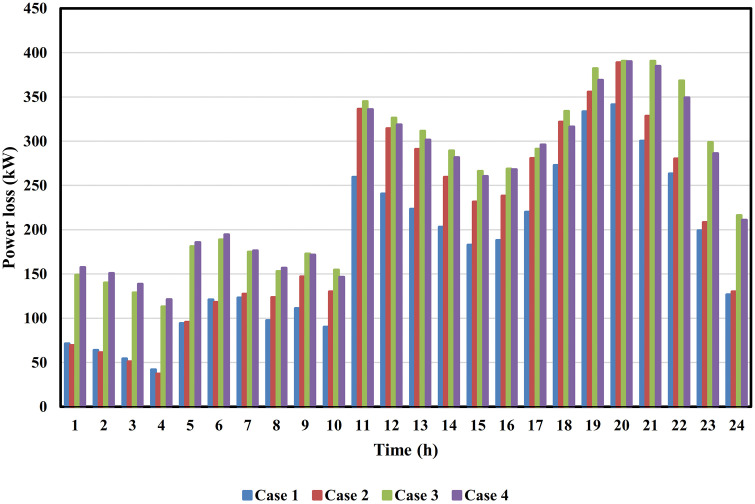
Total active power loss recorded at each hour under scenario 5 for the four case studies: Cairo 59-bus DS.

The effectiveness of the proposed coordination framework in reducing power losses while maintaining them consistently below the base-case levels is clearly illustrated in Figs 42 and 43. The results obtained highlight the significance of employing the proposed stochastic coordination approach to achieve an accurate and realistic estimation of system losses under uncertain operating conditions. As presented, the total power losses remain lower than those of the base case (the original case), even under cases characterized by high DG penetration and intensive EV charging demand, particularly in Cases 3 and 4. This superior performance stems from the careful selection of multi-objective weighting factors, the coordinated management of EV charging, and the integration of SI Volt/VAR control, all of which reinforce the robustness and effectiveness of the proposed optimization model in maintaining efficient and reliable system operation.

**Fig 44:**
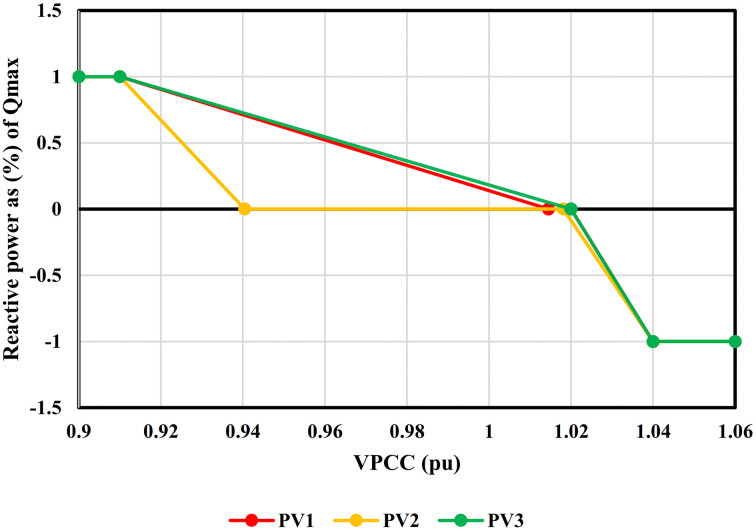
SIs results for IEEE 33-bus DS through Case 4: optimal voltage/VAR control settings at h = 13.

**Fig 45:**
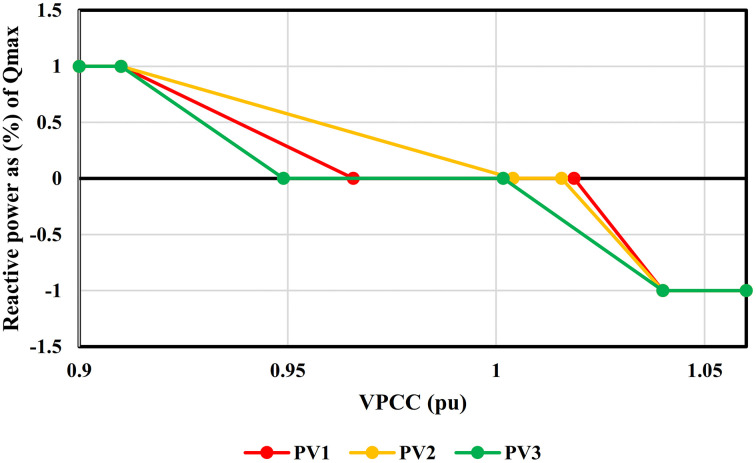
SIs results for Cairo 59-bus DS through Case 4: optimal voltage/VAR control settings at h = 10.

**Fig 46:**
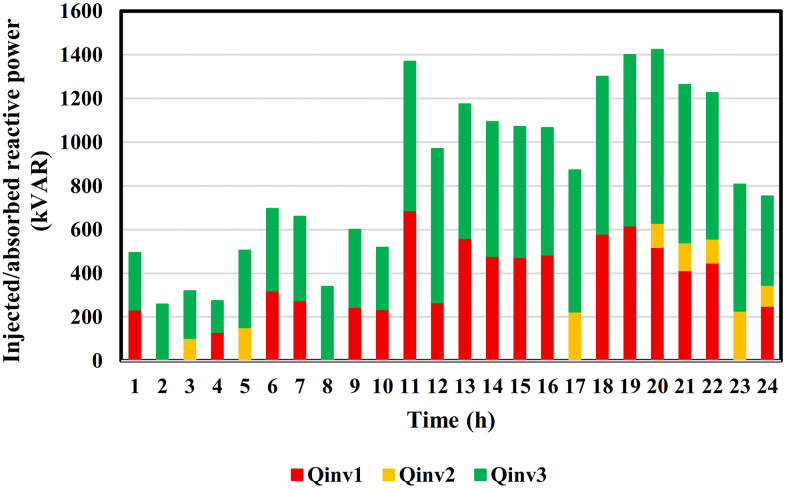
SIs results for IEEE 33-bus DS through Case 4: reactive power profile.

**Fig 47:**
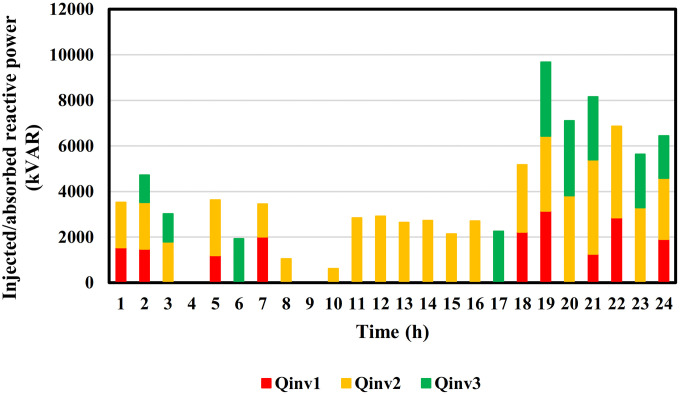
SIs results for Cairo 59-bus DS through Case 4: reactive power profile.

The primary function of SIs is to regulate bus voltages by dynamically injecting or absorbing reactive power in response to system requirements. In this study, the operating parameters of the SIs are strategically configured based on the recorded bus voltage profiles derived from Case 3. Furthermore, Figs 44–47 illustrates the optimized Volt/VAR control characteristics and the corresponding reactive power dispatch of the SIs under scenario 5, demonstrating their crucial contribution to enhancing the voltage profile and improving overall system performance.

### 5.3 Practical communication considerations and system robustness

In practical implementations, coordination among DG units, SIs, and EV charging stations relies on a communication infrastructure that may be subject to latency, data loss, or partial communication failures. These factors can affect the timely exchange of control signals and measurements, potentially influencing the effectiveness of coordinated control strategies.

Communication delays, typically ranging from milliseconds to a few seconds depending on the employed protocol, may introduce outdated information into the optimization process. However, the proposed stochastic framework inherently mitigates this effect by relying on scenario-based planning rather than real-time high-frequency control. Since optimization is performed on an hourly basis using probabilistic representations of system uncertainties, the impact of short-term delays is expected to be limited.

Also, data loss or packet drop events may also occur in practical communication networks. In such cases, modern control systems typically employ data buffering, interpolation, or last-value-hold strategies to maintain operational continuity. The adopted methodology remains robust under such conditions, as the scenario generation and reduction processes capture a wide range of possible system states, thereby providing inherent tolerance to missing or imperfect data. In the case of partial or temporary communication failures, decentralized or local control strategies embedded within SIs and EV chargers can ensure safe operation. For example, local voltage control (e.g., Volt/VAR or Volt/Watt functions) can be activated autonomously when communication with the central controller is unavailable. This fallback mechanism ensures that system constraints are not violated even under degraded communication conditions.

Overall, while communication imperfections are inevitable in real-world deployments, the proposed framework is designed to be resilient due to its reliance on stochastic modeling, reduced dependency on real-time communication, and compatibility with local control mechanisms. Nevertheless, incorporating detailed communication network modeling and cyber-physical co-simulation represents an important direction for future research.

### 5.4 Assessment of the SFOA algorithm

The SFOA algorithm exhibited superior performance in solving the optimization problem under investigation, as evidenced by the comparative outcomes summarized in Tables 5 and 6. Its efficiency was rigorously evaluated against three widely recognized metaheuristic algorithms: Zebra Optimization Algorithm (ZOA) [41]; Grey wolf optimizer (GWO) [42]; and Runge Kutta optimizer (RUN) [43]. The comparison was conducted over 10 independent runs, using 50 search agents and 300 maximum iterations. The tables shown illustrate the convergence characteristics of the four algorithms, highlighting the best and worst solutions obtained for the IEEE 33-bus DS at hour 13 of scenario 5 and the Cairo 59-bus DS at hour 10 of scenario 5 (SC5). The evaluation encompasses Cases 2, 3, and 4, where the influence of EV integration is explicitly analyzed, while Case 1 (DG-only integration) is excluded from this convergence assessment.

**Table 5:** Comparison between the best and worst objective function values of the candidate optimizers in solving Cases 2, 3, and 4 in the IEEE 33-bus test system at hour 13, SC5.

Case	Technique	Best and worst fitness values						$N_{best}$	α(%)
		Best			Worst				
		OF	DG capacity (MW)	EV charging demand (MWh)	OF	DG capacity (MW)	EV charging demand (MWh)		
Case 2	RUN	0.5826	10.43	2.13	0.5826	10.43	2.13	5	50
	ZOA	0.6166	9.12	2.13	0.6237	8.65	2.13	6	60
	GWO	0.5826	10.432	2.13	0.5867	10.103	2.13	8	80
	SFOA	0.5826	10.432	2.13	0.5878	10.061	2.13	8	80
Case 3	RUN	0.5447	10.43	1.58	0.5461	10.432	1.52	5	50
	ZOA	0.6029	7.04	1.05	0.6293	6.26	0.94	7	70
	GWO	0.5451	10.43	1.56	0.5505	10.17	1.04	7	70
	SFOA	0.5446	10.43	1.5763	0.5457	10.43	1.466	8	80
Case 4	RUN	0.5847	11.66	4.41	0.6093	10.616	2.752	4	40
	ZOA	0.5839	12.14	4.33	0.6172	9.19	2.57	6	60
	GWO	0.5696	11.91	4.26	0.5862	11.71	4.26	5	50
	SFOA	0.5669	11.99	4.49	0.5798	10.992	4.84	7	70

**Table 6:** Comparison between the best and worst objective function values of the candidate optimizers in solving Cases 2, 3, and 4 in the Cairo 59-bus test system at hour 10, SC5.

Case	Technique	Best and worst fitness values						$N_{best}$	α(%)
		Best			Worst				
		OF	DG capacity (MW)	EV charging demand (MWh)	OF	DG capacity (MW)	EV charging demand (MWh)		
Case 2	RUN	0.3970	49.78	40.8	0.3978	49.88	40.8	6	60
	ZOA	0.4002	50.17	40.8	0.4108	43.3	40.8	8	80
	GWO	0.3969	49.24	40.8	0.3974	50.48	40.8	7	70
	SFOA	0.3968	48.95	40.8	0.3970	48.11	40.8	9	90
Case 3	RUN	0.3465	59.50	90.70	0.3593	63.26	81.72	4	40
	ZOA	0.3719	51.18	83.50	0.4281	47.40	59.80	4	40
	GWO	0.3451	58.67	88.40	0.3495	56.48	82.69	5	50
	SFOA	0.3443	57.95	87.36	0.3465	59.46	90.28	7	70
Case 4	RUN	0.3830	64.57	92.05	0.4100	52.26	94.27	5	50
	ZOA	0.3869	57.14	90.97	0.4541	59.86	71.92	5	50
	GWO	0.3807	57.95	89.92	0.3822	58.83	95.11	6	60
	SFOA	0.3784	59.14	94.17	0.3835	60.7	96.08	7	70

To further quantify the consistency of the optimization process, a robustness index, denoted as α and defined in (39), is employed. This parameter represents the ratio between the number of times the global best solution was achieved ($N_{best}$) and the total number of runs ($N_{tot}$).


$$\begin{array}{c} \alpha =\frac{N_{best}}{N_{tot}} \end{array}$$ (39)

For IEEE 33-bus system, the ZOA algorithm demonstrated the weakest convergence especially in case 2 and 3 compared to other optimizers. For example, in case 2, which has the lowest decision variables, it converged to 0.6166 which is almost 6% higher than the objective function value that reached by all three other optimizers. This inferior convergence directly impacted the optimal capacities of DG and EVs charging demand, which were nearly 3.39 MW and 0.53 MWh lower, respectively, than the corresponding best values obtained by SFOA in case 3. In addition, it converged to the worst objective function value for all cases in Cairo 59-bus system.

**Fig 48:**
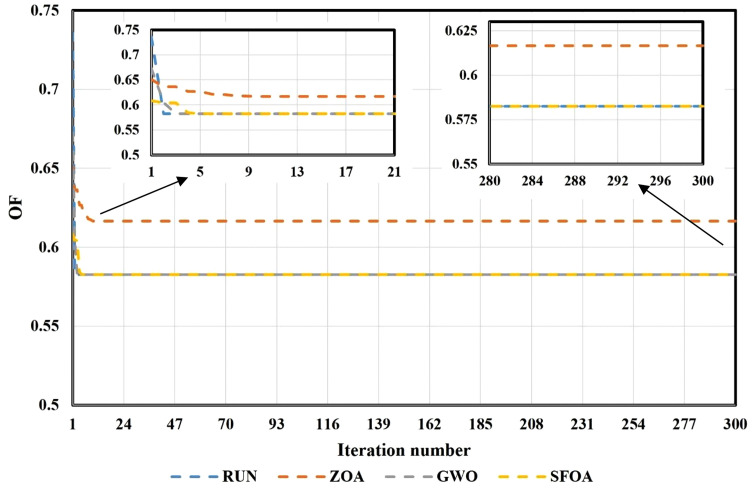
Convergence curves of RUN, ZOA, GWO, and SFOA for the IEEE 33 bus system at hour 13 of scenario 5: Case 2.

**Fig 49:**
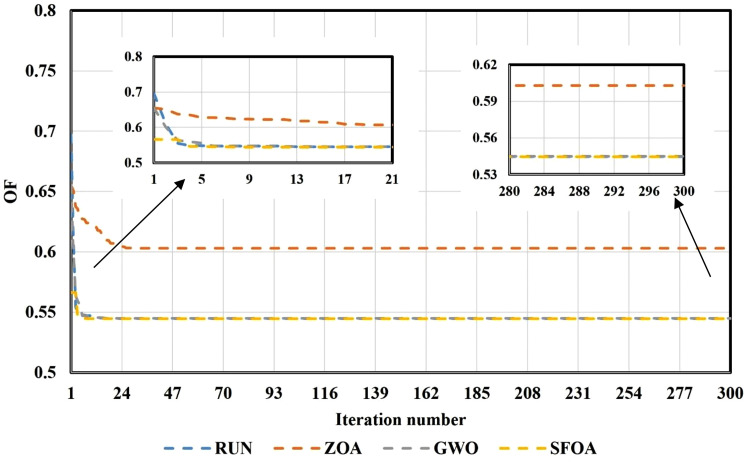
Convergence curves of RUN, ZOA, GWO, and SFOA for the IEEE 33 bus system at hour 13 of scenario 5: Case 3.

**Fig 50:**
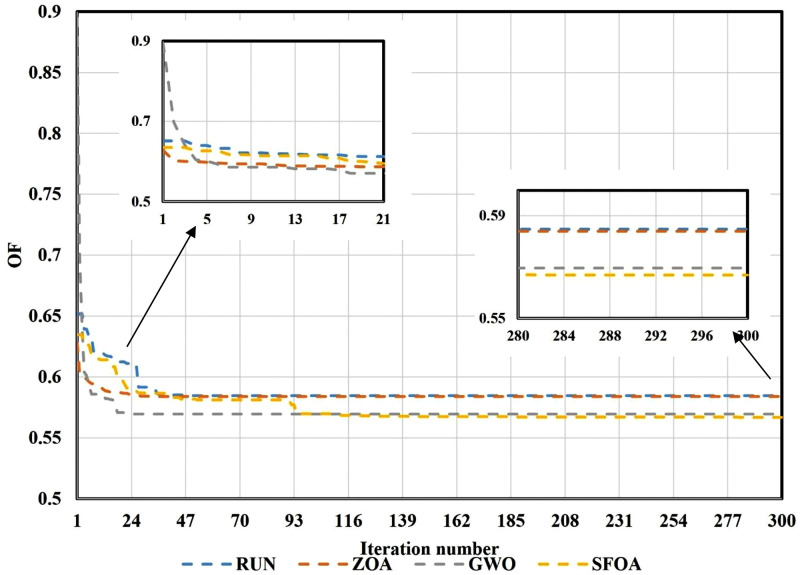
Convergence curves of RUN, ZOA, GWO, and SFOA for the IEEE 33 bus system at hour 13 of scenario 5: Case 4.

**Fig 51:**
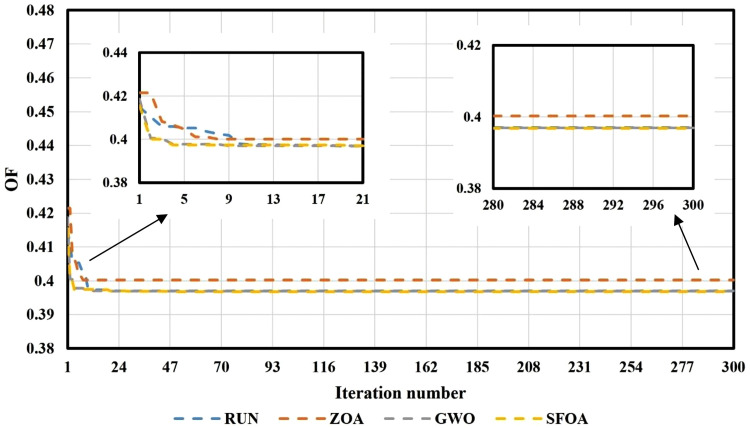
Convergence curves of RUN, ZOA, GWO, and SFOA for Cairo 59 bus system at hour 10 of scenario 5: Case 2.

**Fig 52:**
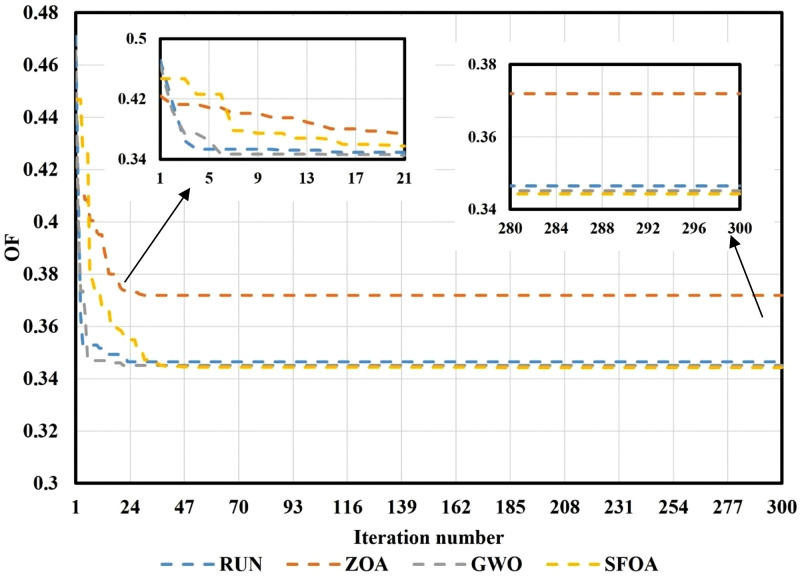
Convergence curves of RUN, ZOA, GWO, and SFOA for Cairo 59 bus system at hour 10 of scenario 5: Case 3.

**Fig 53:**
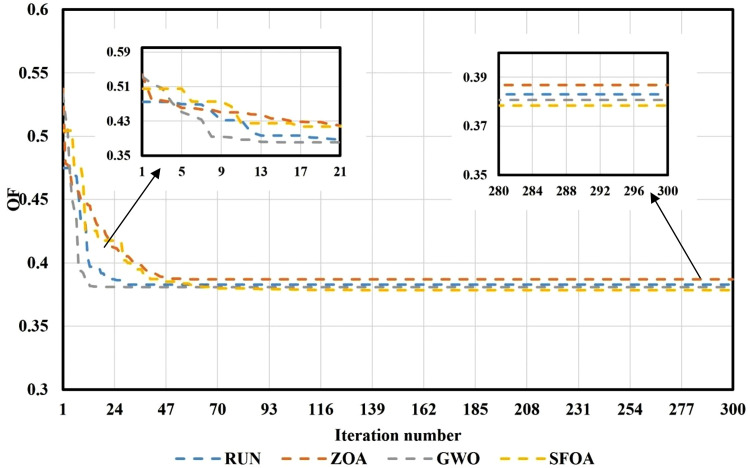
Convergence curves of RUN, ZOA, GWO, and SFOA for Cairo 59 bus system at hour 10 of scenario 5: Case 4.

In contrast, the other optimization techniques achieved acceptable convergence levels; however, SFOA consistently converged to the global best solutions with greater stability and fewer fluctuations across runs. To conclude, the SFOA exhibited the most robust and efficient convergence behavior among all tested cases, achieving the lowest objective function values in every case and maintaining the highest frequency of optimal runs. The comparative results illustrated in Figs 48–53 further confirm the superior convergence speed, reliability, and solution quality of SFOA relative to RUN, ZOA, and GWO algorithms.

In addition to empirical performance assessment, the computational complexity of SFOA is analyzed. Let $N_{p}$ denote the population size, $T_{max}$ the number of iterations, and D the dimensionality of the decision vector. The dominant computational cost arises from objective-function evaluation, which includes one Newton-Raphson load-flow computation and checking all voltage, power loss, inverter, and HC constraints. For a network with $N_{b}$ buses, $N_{l}$ lines, $N_{DG}$ DG units, and $N_{EV}$ EV charging stations, the evaluation cost is:


$$\begin{array}{c} C_{\text{evaluate}}=O(N_{b}+N_{l}+N_{DG}+N_{EV}) \end{array}$$ (40)

Consequently, the overall complexity of SFOA is:


$$\begin{array}{c} O(N_{p}\;T\;(D+C_{\text{evaluate}}))=O(N_{p}\;T\;(D+N_{b}+N_{l}+N_{DG}+N_{EV})) \end{array}$$ (41)

This complexity is comparable to the ZOA, GWO, RUN, but SFOA benefits from fewer hyperparameters and reduced per-iteration overhead, supporting the efficiency observed in the convergence comparisons. Each hour and scenario is solved independently, resulting in 240 parallelizable optimization runs across the 24-hour, 10-scenario study.

To clarify scalability, the SOA runtime increases moderately with system size and EV penetration level. As illustrated in Table 7, the average execution time for the IEEE 33-bus system ranges from approximately 427–547 seconds, while for the larger Cairo 59-bus distribution system, it ranges from 1,710–1,895 seconds under identical algorithm settings. This increase is expected due to the higher dimensionality and additional power flow evaluations required in the larger network. Nevertheless, the overall computational burden remains practical for offline planning studies.

**Table 7:** Computational time of SOA for different case studies and two system sizes, SC5.

System	Case	Time (s)
IEEE 33-bus (*h* = 13)	Case 2	427
IEEE 33-bus (*h* = 13)	Case 3	432
IEEE 33-bus (*h* = 13)	Case 4	547
Cairo 59-bus (*h* = 10)	Case 2	1,710
Cairo 59-bus (*h* = 10)	Case 3	1,728
Cairo 59-bus (*h* = 10)	Case 4	1,895

### 5.5 Identification of the best compromise solution using TOPSIS

To facilitate the interpretation of the multi-objective optimization results presented in Table 2, a multi-criteria decision-making approach based on the Technique for Order Preference by Similarity to Ideal Solution (TOPSIS) is employed to identify the most balanced solution among the considered weighting-coefficient combinations. The evaluation criteria include: (i) DG installed capacity and (ii) EV charging demand, treated as benefit criteria, and (iii) voltage deviation, (iv) power losses, and (v) a penalty term representing constraint violations, treated as cost criteria. All criteria are normalized using vector normalization, and equal weights are assigned because no predefined preference information is available for the considered criteria, ensuring a consistent basis for comparing the alternatives. The inclusion of the penalty criterion allows infeasible solutions to be explicitly penalized without excluding them from the analysis, while its normalization ensures it does not dominate the other criteria.

Following the standard TOPSIS procedure, the positive ideal solution (PIS) and negative ideal solution (NIS) are determined, and the Euclidean distances of each alternative from these reference points are computed to obtain the corresponding closeness coefficients [44].

The TOPSIS results indicate that the proposed weighting combination achieves the highest closeness coefficient (≈ 0.69), thereby representing the best compromise solution among all candidates. This outcome confirms that the selected weighting set provides a well-balanced trade-off between maximizing DG and EV hosting capabilities while maintaining acceptable voltage profiles, minimizing power losses, and ensuring constraint satisfaction.

**Fig 54:**
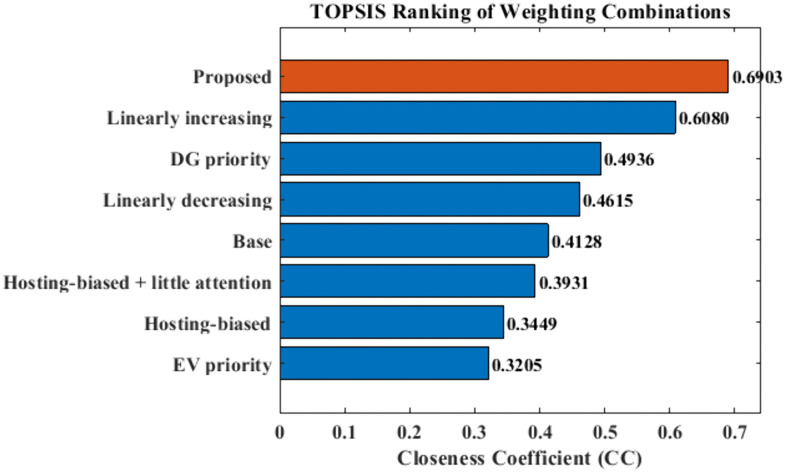
TOPSIS ranking of weighting-coefficient combinations, highlighting the proposed solution as the best compromise.

It is also observed that the ranking remains consistent across small variations in criterion weights, indicating the robustness of the identified solution. For clarity, the TOPSIS ranking of all weighting combinations is illustrated in Fig 54, where the proposed solution is highlighted as the most preferable alternative.

### 5.6 Impact of the number of representative scenarios

**Fig 55:**
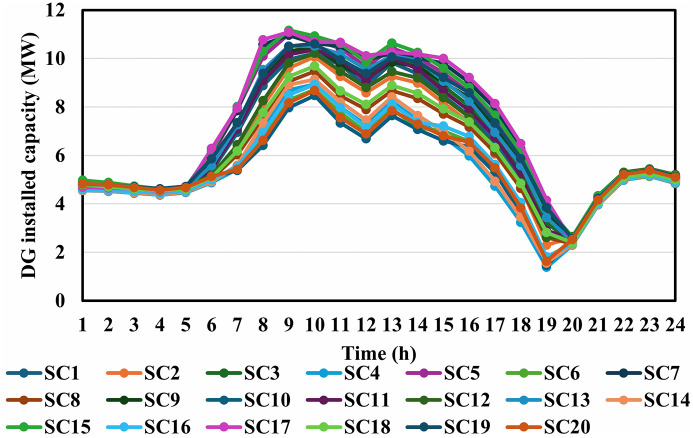
Comparative results derived from the stochastic framework across Case 3 of the IEEE 33-bus system under 20 representative scenarios: hourly recorded DG installed capacity.

**Fig 56:**
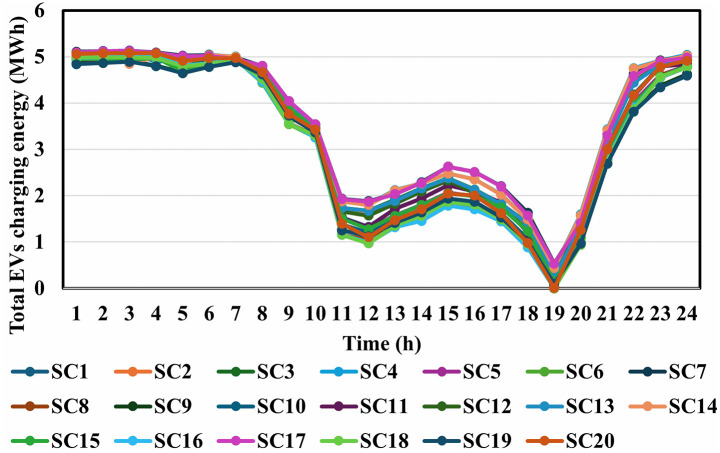
Comparative results derived from the stochastic framework across Case 3 of the IEEE 33-bus system under 20 representative scenarios: hourly recorded EV stations consumption energy.

**Fig 57:**
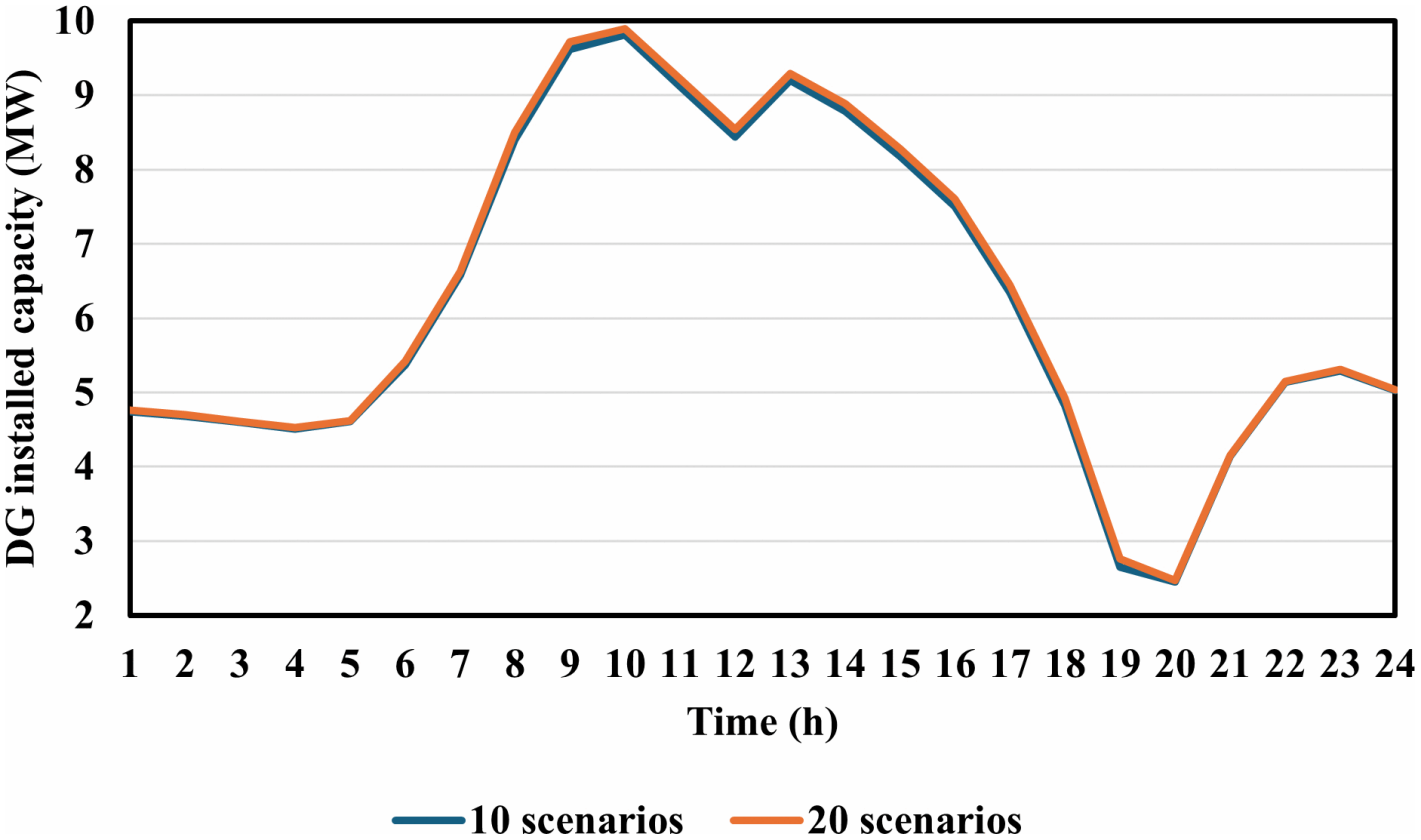
Average value of hourly-basis results for 10 and 20 representative scenarios: DG installed capacity.

**Fig 58:**
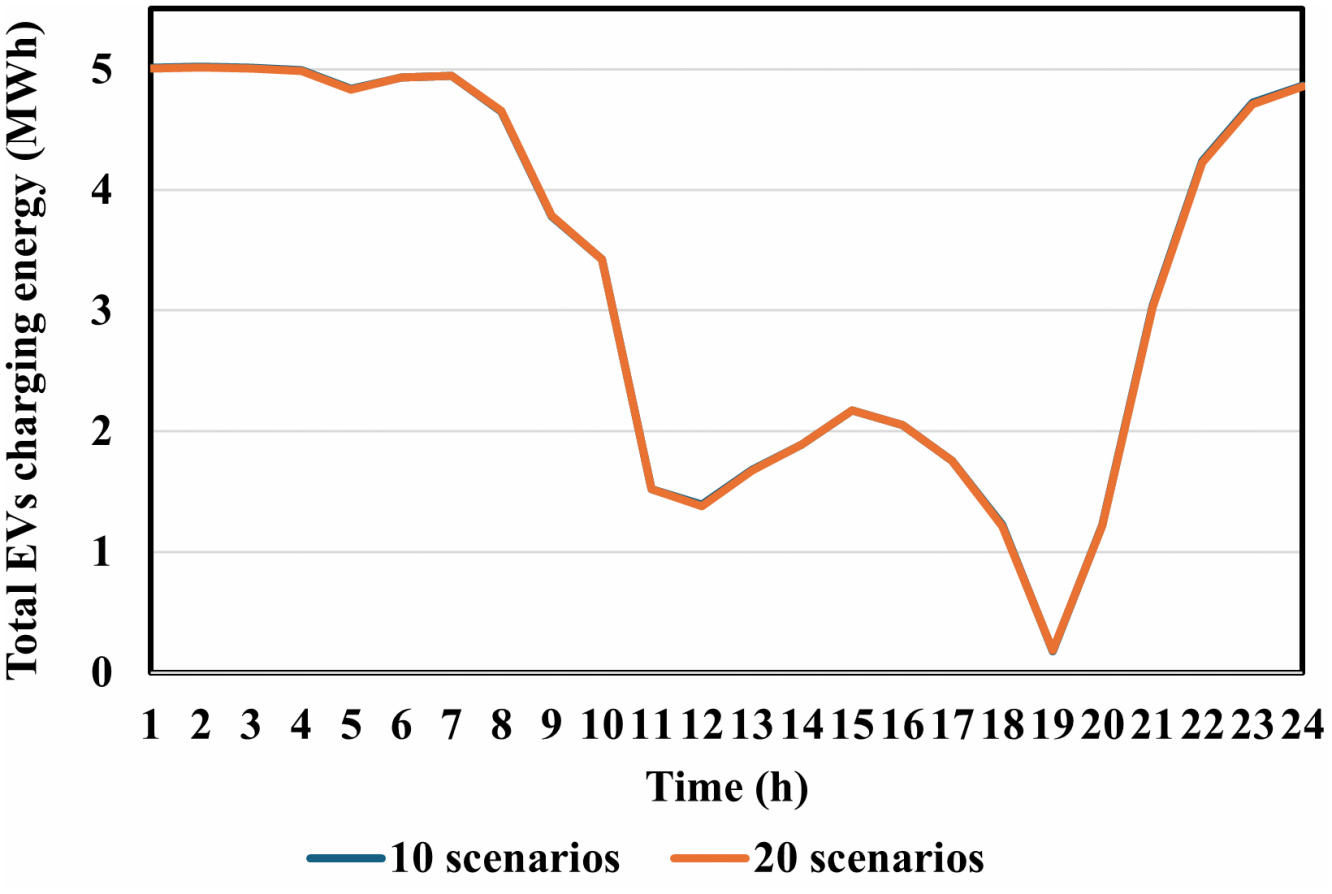
Average value of hourly-basis results for 10 and 20 representative scenarios: EV stations consumption energy.

To evaluate the adequacy of the selected number of representative scenarios, an additional sensitivity analysis was conducted by increasing the number of reduced scenarios obtained via the KDM approach from 10 to 20. The objective is to examine whether a higher number of retained scenarios leads to noticeable improvements in the optimization outcomes. Figs 55 and 56 present the hourly results obtained for Case 3 of the IEEE 33-bus system using 20 representative scenarios, including (a) DG installed capacity and (b) EV charging demand. When compared with the corresponding results from 10 scenarios (Figs 12 and 20), the overall temporal patterns and magnitudes remain highly consistent, indicating that the system’s stochastic behavior is already well captured with 10 scenarios. To further quantify this observation, Figs 57 and 58 illustrate the average hourly values of DG installed capacity and EV charging demand for both 10 and 20 scenarios. It is clear that the average profiles are nearly identical across all time intervals, with only negligible deviations.

In terms of variability, the aggregated standard deviation across the 24-hour horizon shows only marginal differences between the two cases. Specifically, the aggregated standard deviation of DG installed capacity is 14.6166 for 10 scenarios and 14.6749 for 20 scenarios, while for EV charging demand, it is 4.1558 and 4.1768, respectively. This slight increase reflects a more detailed representation of stochastic variability when using a larger number of scenarios; however, the difference remains negligible and does not lead to meaningful changes in system performance or decision-making outcomes.

On the other hand, increasing the number of scenarios substantially increases computational effort, with execution time approximately doubling from 10 to 20 scenarios. Therefore, 10 representative scenarios are considered sufficient, as they achieve an effective balance between computational efficiency and solution accuracy while preserving the essential stochastic characteristics of the system.

## 6 Conclusions

### 6.1 Problem addressed

The accelerating effects of climate change, largely driven by greenhouse gas emissions, have positioned the energy and transportation sectors at the forefront of global sustainability efforts. To advance sustainability goals, many countries are promoting the large-scale integration of RESs and EVs within DSs. While these integrations support decarbonization objectives, they also introduce considerable technical and operational challenges, including voltage deviations, reverse power flows, increased network losses, and thermal overloads. To ensure secure and resilient operation, DSOs must accurately determine the maximum permissible integration levels for distributed generation and EVs, referred to as DG-HC and EV-HC, respectively.

Among various enhancement strategies, optimal EV charging coordination has emerged as an effective and economically sustainable solution compared with costly network reinforcement. However, the stochastic nature of RES generation, load demand, and EV charging behavior introduces significant uncertainty, complicating system planning and operation. Furthermore, the SIs of PV units can dynamically regulate bus voltages through reactive power control, mitigating voltage violations under high RES or EV penetration. Coordinated utilization of these inverters, therefore, plays a crucial role in improving both DG-HC and EV-HC while ensuring the stable and sustainable operation of modern DSs.

### 6.2 Relevance of the problem

Despite extensive efforts to enhance distribution system hosting capacity, most existing studies have limitations that limit their applicability to real-world operations. A major gap lies in the isolated treatment of DG-HC and EV-HC, which overlooks their coupled impacts on network voltage, losses, and power quality under dynamic operating conditions. Moreover, prior approaches often rely on deterministic or simplified assumptions, disregarding the stochastic nature of renewable generation, load variability, and EV charging behavior. Such studies can result in over- or underestimation of available hosting capacities and compromise the reliability of system planning decisions. Besides, while the combined use of smart inverter Volt/VAR control and demand-side management strategies has demonstrated promise, the lack of probabilistic modeling and scenario-based evaluation limits the robustness and generalizability of these findings, particularly in networks with high renewable penetration and fast-growing EV adoption.

### 6.3 Proposed solution

To overcome the identified limitations, this research presents:

- Advanced stochastic multi-objective optimization framework developed from the DSO’s standpoint. The framework simultaneously seeks to maximize the DG-HC and EV-HC while minimizing active power losses and voltage deviations across the network. This coordinated improvement in DG–EV-HC is achieved through a synergistic active and reactive power management scheme.
- EV charging coordination that governs active power regulation, whereas SI optimization ensures effective reactive power control.

The resulting optimization problem is efficiently solved using SFOA, a recent bio-inspired technique known for its balance between exploration and exploitation. Comprehensive comparisons with three well-established metaheuristic algorithms confirm the superior convergence speed and solution quality of the proposed method. Further, the robustness and practical effectiveness of the framework are validated on both the IEEE 33-bus standard DS and a real 59-bus DS in Cairo, Egypt, demonstrating its scalability and applicability.

### 6.4 Key findings and their implications

The obtained results demonstrate that implementing coordinated EV charging strategies within the Cairo 59-bus DS yields a substantial improvement in system performance, increasing the average DG installed capacity from 34.6 MW to 41.7 MW and EV total charging demand from 23.6 MWh to 83.4 MWh, relative to uncoordinated scenarios. For the IEEE 33-bus DS, DG installed capacity increases from 5.5 MW to nearly 7 MW, and EV charging demand rises from 1.18 MWh to 3.2 MWh. When EV charging coordination is integrated with SI Volt/VAR control under the proposed synergistic framework, the improvements become even more pronounced: in the Cairo 59-bus DS, DG capacity reaches 49.6 MW and EV demand 89.1 MWh, while in the IEEE 33-bus DS, DG and EV capacities increase to 8.7 MW and 4.59 MWh, respectively. This coordinated operation also leads to a marked improvement in voltage profile, with the mean voltage deviation index reduced from 50.3% to 29.8% in the IEEE 33-bus network, underscoring the robustness and technical efficacy of the proposed approach across diverse system configurations.

Sensitivity analysis conducted on the IEEE 33-bus system at hour 13 under SC5 reveals that the DG hosting capacity varies between 7.27 and 18.89 MW, and the EV charging demand ranges from 2.03 to 5.42 MWh across different weighting priorities. The weighting set (0.3, 0.3, 0.1, 0.3) was selected as within the feasible range, avoids constraint violations, and achieves the best balance between HC enhancement, voltage regulation, and reliability, highlighting the robustness of the proposed multi-objective formulation.

Although the proposed framework demonstrates substantial improvements under idealized DSO coordination, practical deployment may face limitations such as communication delays, partial observability, and limited controllability of distributed resources. These factors could reduce the realized DG and EV hosting capacities, affect voltage regulation, or increase power losses.

Future work will explore hierarchical or decentralized control strategies and robust mechanisms to mitigate latency or faults, as well as the integration of additional enhancement mechanisms, including battery energy storage systems (BESSs), V2G activation, and active power curtailment, to further strengthen system hosting capacity under realistic operational conditions. Additionally, future work may extend the proposed stochastic optimization framework to support additional grid services beyond planning applications, particularly through the coordinated control of DG and EV charging systems. In this context, the framework can be adapted to support services such as frequency control via active power management and system restoration under black-start conditions by incorporating dynamic modeling and multi-timescale control strategies.

## Supporting information

S1 File MATLAB code includes data of the two systems used in the manuscript. Data of the IEEE 33 and Cairo 59 bus systems.
(DOCX)

S2 File Zenodo link files. (ZIP)
