# Supplementary material for: Joint optimization of smart inverters and EV charging coordination for enhanced DG-EV hosting capacity under uncertain conditions for resilient distribution systems
Source: PLoS One. 2026 Jul 6;21(7):e0350725. doi: 10.1371/journal.pone.0350725 (PMC13336192; doi:10.1371/journal.pone.0350725)
Supplement: S1 File — Data of the IEEE 33 and Cairo 59 bus systems. (DOCX) [file pone.0350725.s001.docx]

MATLAB code includes data of the two systems used in the manuscript.

IEEE 33 bus system

% Bus No Vm Theta Pg Qg P1 Q1 Qmax Qmin Type

busdata= [ 1 1.000 0 0 0 0 0 0 0 1;

2 1.000 0 0 0 100 60 0 0 3;

3 1.000 0 0 0 90 40 0 0 3;

4 1.000 0 0 0 120 80 0 0 3;

5 1.000 0 0 0 60 30 0 0 3;

6 1.000 0 0 0 60 20 0 0 3;

7 1.000 0 0 0 200 100 0 0 3;

8 1.000 0 0 0 200 100 0 0 3;

9 1.000 0 0 0 60 20 0 0 3;

10 1.000 0 0 0 60 20 0 0 3;

11 1.000 0 0 0 45 30 0 0 3;

12 1.000 0 0 0 60 35 0 0 3;

13 1.000 0 0 0 60 35 0 0 3;

14 1.000 0 0 0 120 80 0 0 3;

15 1 0 0 0 60 10 0 0 3;

16 1 0 0 0 60 20 0 0 3;

17 1 0 0 0 60 20 0 0 3;

18 1 0 0 0 90 40 0 0 3;

19 1 0 0 0 90 40 0 0 3;

20 1 0 0 0 90 40 0 0 3;

21 1 0 0 0 90 40 0 0 3;

22 1 0 0 0 90 40 0 0 3;

23 1 0 0 0 90 50 0 0 3;

24 1 0 0 0 420 200 0 0 3;

25 1 0 0 0 420 200 0 0 3;

26 1 0 0 0 60 25 0 0 3;

27 1 0 0 0 60 25 0 0 3;

28 1 0 0 0 60 20 0 0 3;

29 1 0 0 0 120 70 0 0 3;

30 1 0 0 0 200 600 0 0 3;

31 1 0 0 0 150 70 0 0 3;

32 1 0 0 0 210 100 0 0 3;

33 1 0 0 0 60 40 0 0 3];

% Susceptance

Shuntdata=[0 0 0 0 0 0 0 0 0 0 0 0 0 0 0 0 0 0 0 0 0 0 0 0 0 0 0 0 0 0 0 0 0]' ;

linedata=[ 1 1 2 0.0922 0.0470 0 1;

2 2 3 0.4930 0.2511 0 1;

3 3 4 0.3660 0.1864 0 1;

4 4 5 0.3811 0.1941 0 1;

5 5 6 0.8190 0.7070 0 1;

6 6 7 0.1872 0.6188 0 1;

7 7 8 0.7114 0.2351 0 1;

8 8 9 1.0300 0.7400 0 1;

9 9 10 1.0440 0.7400 0 1;

10 10 11 0.1966 0.0650 0 1;

11 11 12 0.3744 0.1238 0 1;

12 12 13 1.4680 1.1550 0 1;

13 13 14 0.5416 0.7129 0 1;

14 14 15 0.5910 0.5260 0 1;

15 15 16 0.7463 0.5450 0 1;

16 16 17 1.2890 1.7210 0 1;

17 17 18 0.7320 0.5740 0 1;

18 2 19 0.1640 0.1565 0 1;

19 19 20 1.5042 1.3554 0 1;

20 20 21 0.4095 0.4784 0 1;

21 21 22 0.7089 0.9373 0 1;

22 3 23 0.4512 0.3083 0 1;

23 23 24 0.8980 0.7091 0 1;

24 24 25 0.8960 0.7011 0 1;

25 6 26 0.2030 0.1034 0 1;

26 26 27 0.2842 0.1447 0 1;

27 27 28 1.0590 0.9337 0 1;

28 28 29 0.8042 0.7006 0 1;

29 29 30 0.5075 0.2585 0 1;

30 30 31 0.9744 0.9630 0 1;

31 31 32 0.3105 0.3619 0 1;

32 32 33 0.3410 0.5302 0 1];

Cairo 59 bus system

busdata=[ 1 1 0 0 0 0 0 0 0 1

2 1 0 0 0 1470.6 626.49 0 0 3

3 1 0 0 0 367.66 156.62 0 0 3

4 1 0 0 0 735.32 313.24 0 0 3

5 1 0 0 0 735.32 313.24 0 0 3

6 1 0 0 0 49.02 20.88 0 0 3

7 1 0 0 0 2944.8 1254.5 0 0 3

8 1 0 0 0 735.32 313.24 0 0 3

9 1 0 0 0 147.06 62.65 0 0 3

10 1 0 0 0 1176.5 501.19 0 0 3

11 1 0 0 0 0 0 0 0 3

12 1 0 0 0 735.32 313.24 0 0 3

13 1 0 0 0 1470.6 626.49 0 0 3

14 1 0 0 0 735.32 313.24 0 0 3

15 1 0 0 0 273.12 116.35 0 0 3

16 1 0 0 0 735.32 313.24 0 0 3

17 1 0 0 0 735.32 313.24 0 0 3

18 1 0 0 0 735.32 313.24 0 0 3

19 1 0 0 0 210.09 89.5 0 0 3

20 1 0 0 0 735.32 313.24 0 0 3

21 1 0 0 0 735.32 313.24 0 0 3

22 1 0 0 0 1838.3 783.11 0 0 3

23 1 0 0 0 735.32 313.24 0 0 3

24 1 0 0 0 1470.6 626.49 0 0 3

25 1 0 0 0 423.68 180.49 0 0 3

26 1 0 0 0 735.32 313.24 0 0 3

27 1 0 0 0 696.8 296.84 0 0 3

28 1 0 0 0 735.32 313.24 0 0 3

29 1 0 0 0 2213 942.72 0 0 3

30 1 0 0 0 735.32 313.24 0 0 3

31 1 0 0 0 1470.6 626.49 0 0 3

32 1 0 0 0 735.32 313.24 0 0 3

33 1 0 0 0 735.32 313.24 0 0 3

34 1 0 0 0 735.32 313.24 0 0 3

35 1 0 0 0 367.66 156.62 0 0 3

36 1 0 0 0 367.66 156.62 0 0 3

37 1 0 0 0 283.62 120.82 0 0 3

38 1 0 0 0 1103 469.87 0 0 3

39 1 0 0 0 168.07 71.6 0 0 3

40 1 0 0 0 406.18 173.03 0 0 3

41 1 0 0 0 735.32 313.24 0 0 3

42 1 0 0 0 735.32 313.24 0 0 3

43 1 0 0 0 2209.5 941.23 0 0 3

44 1 0 0 0 735.32 313.24 0 0 3

45 1 0 0 0 735.32 313.24 0 0 3

46 1 0 0 0 735.32 313.24 0 0 3

47 1 0 0 0 735.32 313.24 0 0 3

48 1 0 0 0 735.32 313.24 0 0 3

49 1 0 0 0 735.32 313.24 0 0 3

50 1 0 0 0 2209.5 941.23 0 0 3

51 1 0 0 0 0 0 0 0 3

52 1 0 0 0 1470.6 626.49 0 0 3

53 1 0 0 0 1103 469.87 0 0 3

54 1 0 0 0 735.32 313.24 0 0 3

55 1 0 0 0 1470.6 626.49 0 0 3

56 1 0 0 0 1470.6 626.49 0 0 3

57 1 0 0 0 1470.6 626.49 0 0 3

58 1 0 0 0 735.32 313.24 0 0 3

59 1 0 0 0 171.57 73.09 0 0 3];

% Susceptance

Shuntdata=[0 0 0 0 0 0 0 0 0 0 0 0 0 0 0 0 0 0 0 0 0 0 0 0 0 0 0 0 0 0 0 0 0 0 0 0 0 0 0 0 0 0 0 0 0 0 0 0 0 0 0 0 0 0 0 0 0 0 0]' ;

linedata=[ 1 1 2 0.0728 0.0435 0 1

2 2 3 0.1229 0.0735 0 1

3 3 4 0.0809 0.0483 0 1

4 4 5 0.0323 0.0193 0 1

5 5 6 0.0323 0.0193 0 1

6 6 7 0.0485 0.029 0 1

7 7 8 0.0841 0.0502 0 1

8 8 9 0.1618 0.0967 0 1

9 9 10 0.0388 0.0232 0 1

10 10 11 0.0453 0.027 0 1

11 11 12 0.0533 0.0319 0 1

12 1 13 0.0364 0.0217 0 1

13 13 14 0.0113 0.0067 0 1

14 14 15 0.0404 0.0241 0 1

15 15 16 0.0533 0.0319 0 1

16 16 17 0.0275 0.0164 0 1

17 17 18 0.0517 0.0309 0 1

18 18 19 0.1537 0.0918 0 1

19 19 20 0.0355 0.0212 0 1

20 1 21 0.0129 0.0077 0 1

21 21 22 0.2119 0.1266 0 1

22 22 23 0.0728 0.0435 0 1

23 1 24 0.1019 0.0609 0 1

24 24 25 0.1779 0.1063 0 1

25 25 26 0.0064 0.0038 0 1

26 26 27 0.0453 0.027 0 1

27 27 28 0.021 0.0125 0 1

28 28 29 0.0622 0.0372 0 1

29 29 30 0.0105 0.0062 0 1

30 1 31 0.0283 0.0169 0 1

31 31 32 0.2427 0.145 0 1

32 32 33 0.0339 0.0203 0 1

33 33 34 0.0614 0.0367 0 1

34 34 35 0.0242 0.0145 0 1

35 35 36 0.021 0.0125 0 1

36 36 37 0.0242 0.0145 0 1

37 37 38 0.0922 0.0551 0 1

38 38 39 0.0032 0.0019 0 1

39 1 40 0.0533 0.0319 0 1

40 40 41 0.0695 0.0415 0 1

41 41 42 0.0097 0.0058 0 1

42 42 43 0.0485 0.029 0 1

43 43 44 0.3316 0.1982 0 1

44 44 45 0.0113 0.0067 0 1

45 45 46 0.0614 0.0367 0 1

46 46 47 0.0436 0.0261 0 1

47 47 48 0.0436 0.0261 0 1

48 48 49 0.1164 0.0696 0 1

49 49 50 0.0355 0.0212 0 1

50 1 51 0.0339 0.0203 0 1

51 51 52 0.0097 0.0058 0 1

52 52 53 0.0129 0.0077 0 1

53 53 54 0.0161 0.0096 0 1

54 54 55 0.0647 0.0386 0 1

55 55 56 0.2103 0.1257 0 1

56 1 57 0.0752 0.0449 0 1

57 57 58 0.1456 0.087 0 1

58 58 59 0.0242 0.0145 0 1];
