# Supplementary material for: Joint optimization of smart inverters and EV charging coordination for enhanced DG-EV hosting capacity under uncertain conditions for resilient distribution systems
Source: PLoS One. 2026 Jul 6;21(7):e0350725. doi: 10.1371/journal.pone.0350725 (PMC13336192; doi:10.1371/journal.pone.0350725)
Supplement: Attachment — Submitted filename: reviewers comments report V4-Shady.docx [file pone.0350725.s003.docx]

**Response to the Reviewer Comments**

**Dear Editor and Reviewers**

*The manuscript has been revised based on the precise comments of the respected editor and reviewer. The authors hope the revised version of the manuscript fulfills all expectations of the reviewer and editor. The responses provided by the authors to your comments are as follows:*

**Editor comment**

**We note that Figures 3 and 7 in your submission may contain copyrighted images.**

**Authors’ response:** We thank the Editor for pointing out the potential copyright issues in Figures 3 and 7. To fully comply with PLOS ONE’s copyright and licensing requirements, we have removed both Figure 3 and Figure 7 from the revised manuscript. As these figures were not essential to the technical content or results, their removal does not affect the methodology, analyses, or conclusions of the study.

**Authors’ action:** Figures 3 and 7 have been removed from the main manuscript as well as from the supplementary files. In addition, all remaining figures in the revised manuscript are original, high-resolution, and fully compliant with PLOS ONE’s licensing policy.

**Reviewer #1**

**Reviewer #1, Concern #1:** **The abstract does not follow a research-article arc (problem → proposed method → quantitative outcomes) and is verbose; the Introduction is short, reads like a report, and relies on long bullet lists that dilute technical focus.**

**Authors’ response:** We thank the reviewer for the feedback regarding the abstract and the Introduction. The abstract has been revised to follow a clear research-article arc, emphasizing the problem, the proposed method, and key quantitative outcomes in a concise manner. The updated abstract is as follows:

“

*The rapid growth of renewable-based distributed generation (DG) and electric vehicles (EVs) poses significant operational challenges for distribution systems (DSs), particularly under uncertainties in renewable output, load demand, and EV charging behavior. Distribution system operators must therefore evaluate and enhance both DG hosting capacity (DG-HC) and EV hosting capacity (EV-HC) while maintaining voltage security and reducing losses. This study presents a stochastic, multi-objective optimization framework that jointly coordinates smart inverter (SI)-based Volt/VAR control and EV charging scheduling to simultaneously maximize DG-HC and EV-HC and minimize active power losses and voltage deviation. The framework integrates active power management through EV charging coordination and reactive power support via optimally deployed SIs. The resulting multi-objective problem is solved using the Starfish Optimization Algorithm (SFOA) and benchmarked against three established metaheuristics. The methodology is validated on the IEEE 33-bus system and a real 59-bus distribution network in Cairo, Egypt. Results show that coordinated SI–EV control increases DG-HC and EV-HC by up to 74% and 89%, respectively, and achieves voltage deviation reductions of 55% in the IEEE 33-bus system and 11% in the Cairo DS. Comparative analysis confirms that SFOA provides superior convergence and solution quality relative to the competing techniques.*

”

In addition, the Introduction section has been substantially expanded and now includes 27 updated references. Its writing style has been enhanced and revised to improve technical clarity and readability. These changes provide a stronger and more focused context for the study and ensure up-to-date coverage of multi-objective HC assessment, coordinated SI–EV scheduling, and uncertainty-aware optimization.

**Authors’ action:** The abstract has been fully revised to follow a clear research-article structure, emphasizing the core problem, the proposed coordination framework, and key quantitative outcomes. The Introduction has been substantially expanded and rewritten to improve technical clarity, remove verbose bullet lists, and provide a focused discussion of the relevant literature. A total of 27 updated references have been included to ensure comprehensive coverage of multi-objective hosting capacity assessment, coordinated SI–EV scheduling, and uncertainty-aware optimization, thereby strengthening the context and contribution of the study.

**Reviewer #1, Concern #2:** **Prior work coverage is shallow and uneven: recent (2023–2025) state-of-the-art on multi-objective hosting capacity, coordinated SI+EV scheduling, chance-/robust-constrained formulations, and standard Pareto baselines is underrepresented; self-citation and regional clustering are noticeable.**

**Authors’ response:** We sincerely appreciate the reviewer’s observation regarding the depth and balance of the prior work coverage. In response, we have undertaken a substantial revision of the literature review to ensure a comprehensive, up-to-date, and methodologically diverse discussion of recent advancements. Specifically, we expanded the literature subsection by incorporating eight additional peer-reviewed studies published between 2023 and 2025, covering multi-objective hosting capacity assessment, coordinated smart-inverter and EV scheduling, chance- and robust-constrained formulations, and recent multi-objective optimization baselines. The revised Related Work section now synthesizes and contrasts 22 recent and diverse references, providing a significantly stronger contextual foundation for the novelty of our proposed framework.

Also, to enhance transparency and provide clearer differentiation from existing literature, we have substantially expanded Table 1, “Overview of the key contributions of this study in comparison with selected works from the literature.” The revised table now contrasts contemporary studies across multiple technical dimensions—including uncertainty modeling, SI-based reactive power support, EV charging coordination, and DG/EV hosting capacity objectives, thus presenting a more structured and rigorous positioning of our contributions.

Regarding self-citation, four of our prior works appeared in the original manuscript (numbered as [6], [8], [21], and [29] in the original manuscript; these are renumbered as [6], [8], [24], and [34] in the revised manuscript). To avoid unnecessary self-citation, references [6] and [34] in the revised manuscript have been replaced with more recent and relevant studies. However, references [8] and [24] are directly pertinent to the technical foundations of this work and could not be removed without compromising the continuity of the research context. In the revised manuscript, we explicitly contrast the current study with these two prior works and clearly highlight how the present framework addresses the research gaps that remained open in those earlier contributions.

Table 1. Overview of the key contributions of this study in comparison with selected works from the literature.

| Assessment Parameters | | [10], [16] | [11],[15] | [14] | [18] | [19]–[21] | [23] | [22] | [25] | [4] | [13] | [26] | [8] | This work |
| --- | --- | --- | --- | --- | --- | --- | --- | --- | --- | --- | --- | --- | --- | --- |
| HC evaluation | | 𝕐 | 𝕐 | 𝕐 | ℕ | 𝕐 | 𝕐 | 𝕐 | ℕ | 𝕐 | 𝕐 | 𝕐 | 𝕐 | 𝕐 |
| System uncertainties | | 𝕐 | 𝕐 | 𝕐 | ℕ | ℕ | 𝕐 | ℕ | 𝕐 | ℕ | ℕ | ℕ | ℕ | 𝕐 |
| DG technology variety | | ℕ | ℕ | ℕ | ℕ | ℕ | ℕ | ℕ | 𝕐 | ℕ | ℕ | 𝕐 | 𝕐 | 𝕐 |
| Active power support | | 𝕐 | 𝕐 | 𝕐 | ℕ | 𝕐 | 𝕐 | 𝕐 | 𝕐 | 𝕐 | 𝕐 | 𝕐 | 𝕐 | 𝕐 |
| Reactive power support | | ℕ | 𝕐 | ℕ | 𝕐 | ℕ | 𝕐 | 𝕐 | ℕ | ℕ | 𝕐 | ℕ | 𝕐 | 𝕐 |
| EVs charging scheduling | | 𝕐 | 𝕐 | 𝕐 | ℕ | ℕ | ℕ | ℕ | 𝕐 | 𝕐 | 𝕐 | 𝕐 | 𝕐 | 𝕐 |
| Optimization objectives | DG-HC maximization | 𝕐 | 𝕐 | 𝕐 | ℕ | 𝕐 | 𝕐 | 𝕐 | ℕ | 𝕐 | 𝕐 | 𝕐 | 𝕐 | 𝕐 |
|  | EV-HC maximization | ℕ | ℕ | ℕ | ℕ | ℕ | ℕ | ℕ | 𝕐 | 𝕐 | ℕ | 𝕐 | 𝕐 | 𝕐 |
|  | Loss reduction | 𝕐 | ℕ | ℕ | ℕ | ℕ | ℕ | ℕ | ℕ | ℕ | 𝕐 | 𝕐 | 𝕐 | 𝕐 |
|  | Voltage profile enhancement | ℕ | ℕ | ℕ | 𝕐 | ℕ | 𝕐 | ℕ | ℕ | ℕ | ℕ | ℕ | ℕ | 𝕐 |

**Authors’ action:** The literature review has been substantially strengthened by adding eight recent (2023–2025) studies and expanding the discussion to a total of 22 contrasted references, ensuring balanced and up-to-date coverage of multi-objective hosting capacity assessment, coordinated SI–EV scheduling, and uncertainty-aware optimization. In addition, Table 1 has been expanded to include additional comparison dimensions and a broader set of contemporary studies. To address self-citation concerns, two earlier self-cited references were replaced with more relevant recent works, while the two technically indispensable ones were retained and explicitly contrasted with the present framework to clarify the advancements achieved.

**Reviewer #1, Concern #3: The multi-objective formulation is ill-posed: use of reciprocals of hosting-capacity indices is numerically unstable, and the optimization block lacks an explicit aggregation over time and scenarios, creating ambiguity about what is being minimized.**

**Authors’ response:** We thank the reviewer for this insightful comment. In the revised manuscript, the multi-objective optimization framework has been substantially reformulated to ensure numerical stability, proper scaling, and full clarity regarding the role of hours and scenarios.

**Removal of all reciprocal terms**

We fully agree that the use of $1/HC$ in the original objective-function formulation could lead to numerical instability when hosting-capacity values become small. In the revised work, all reciprocal terms have been removed. Additionally, hosting-capacity indices are now used in the form $\left( 1 - HC \right)$, which is bounded, smooth, and numerically stable on $\left[ 0 , 1 \right]$. This eliminates the ill-posedness flagged initially by the reviewer. The modified objective function becomes:

$$minof=\min\{w_{1}\left( 1-{DG-HC}_{s}^{k} \right)+w_{2}\left( 1-{EV-HC}_{s}^{k} \right)++w_{3}{(DI}_{s}^{k}/{DI}_{BC})+w_{4}{(PLI}_{s}^{k})\}, \forall s, \forall k$$

(25)

**Revised and normalized definitions of DG-HC and EV-HC**

$${DG-HC}_{s}^{k}=\frac{\sum_{j=1}^{N} {P_{{WT}_{s,j}}}^{k}+ \sum_{j=1}^{M} {P_{{PV}_{s,j}}}^{k}}{\sum_{j=1}^{N} P_{WT,j}^{rated}+ \sum_{j=1}^{M} P_{PV,j}^{rated}}$$

(20)

$${EV-HC}_{s}^{k}=\frac{\sum_{r=1}^{C} E_{{EV}_{s,r}}^{k}}{\sum_{r=1}^{C} E_{EV,r}^{rated}}$$

(21)

This formulation ensures that both indices lie strictly in [0,1]. Both are normalized using physically meaningful upper bounds (installed DG capacity and EV charger ratings). No variable can be artificially inflated (“gamed”), eliminating the technical weakness noted by the reviewer.

**Explicit clarification of scenario handling and time resolution**

We revised the manuscript to clearly explain that the optimization is not a single stochastic expected-value problem, instead, each reduced scenario $s$ and each hour $k$ represent a different operating condition under uncertainty. Thus, a separate deterministic optimization problem is solved independently for each pair $(k,s)$. Therefore, no averaging or aggregation across hours or scenarios is missing; the formulation is intentionally scenario-wise and hour-wise to obtain detailed hourly hosting-capacity profiles.

**Re-running all simulations with the corrected objective function**

Because the corrected objective function materially changes the optimization landscape, all experiments and results have been recomputed using the revised, well-posed formulation. The updated numerical results now reflect the corrected hosting-capacity definitions and the stable objective structure.

Therefore, the optimization framework in the revised manuscript is now mathematically well posed, free of reciprocal and unbounded terms, fully normalized, explicitly scenario-wise and hour-wise, and consistent with hosting-capacity practices in the literature.

**Authors’ action:** In response to the reviewer’s observation, we have completely reformulated the multi-objective function to ensure mathematical well-posedness and eliminate ambiguity. Precisely, (i) all reciprocal hosting-capacity terms were removed and replaced with bounded, normalized expressions using $1-HC$, eliminating numerical instability; (ii) DG-HC and EV-HC were redefined as normalized, capacity-based indices bounded in $[0,1]$; and (iii) scenario and hour treatment was explicitly clarified, showing that each scenario–hour pair is optimized independently rather than aggregated, thereby removing ambiguity regarding minimization across time and scenarios. The multi-objective function formulation subsection has been entirely rewritten, and all results were regenerated using the corrected, well-posed formulation.

**Reviewer #1, Concern #4:** **Electrical feasibility is incompletely modeled: Volt/VAR set-points are not coupled to inverter apparent-power limits, rate/step constraints, or guaranteed 4-point curve order; feeder ampacity/protection assumptions are insufficiently specified.**

**Authors’ response:** We thank the reviewer for highlighting the need for a more rigorous and electrically consistent formulation of the smart-inverter Volt/VAR model.

In the revised manuscript, the Volt/VAR characteristic is now fully specified with explicit mathematical expressions for the four-point curve, including ordered breakpoints, negative slopes, and a guaranteed monotonic, piecewise-affine profile. The intermediate points $V_{2j}$ and $V_{3j}$ are computed directly from the decision-variable slopes $m_{1j}$ and $m_{2j}$, ensuring consistent deadband behavior. Although the original code fully enforced Volt/VAR monotonicity, breakpoint ordering, and inverter apparent-power capability limits, these implementation details were not sufficiently highlighted in the previous manuscript.

The revised version now explains these mechanisms explicitly and provides the complete mathematical formulation to reflect the model already used in the simulations. These explanations have been added to the revised manuscript in the Smart Inverter Modeling subsection:

“

*To ensure electrical feasibility, this work adopts an explicit, capability-consistent formulation of the four-segment Volt/VAR characteristic, with constraints guaranteeing monotonicity, correct ordering of curve points, and compliance with inverter apparent-power limits. Each inverter* $j$ *uses a fixed low-voltage saturation point* $V_{1}=0.91\text{ pu}$ *and the high-voltage saturation point* $V_{4}=1.04\text{ }\text{ }\text{pu}$*. Two decision variables define the slopes in the lower and upper regions:*

$$m_{1}\leq0 , m_{2}\leq0$$

(32)

*The intermediate breakpoints* $V_{2j}$ *and* $V_{3j}$ *are not free variables; they are deterministically computed from the slopes:*

$$V_{2_{j}}=\frac{-1}{m_{1_{j}}}+V_{1}$$

(33)

$$V_{3_{j}}=\frac{1}{m_{2_{j}}}+V_{4}$$

(34)

*These expressions guarantee that the deadband width adjusts consistently with the selected slopes. Ordering and monotonicity are enforced through the explicit constraints implemented via penalty terms in the optimization to prevent infeasible curve shapes.*

$$V_{1}<V_{2_{j}}<V_{3_{j}}<V_{4}$$

(35)

The per-unit reactive power as a function of the predefined voltage at the PCC $q_{j}(v)$ is defined through a continuous piecewise-affine Volt/VAR characteristic:

$$q_{j}\left( v \right)=\left\{ \begin{aligned} 1 v\leq V_{1} \\ m_{1_{j}}\left( v-V_{1} \right)+1 V_{1}<v<V_{2_{j}} \\ 0 V_{2_{j}}<v<V_{3_{j}} \\ m_{2_{j}}\left( v-V_{4} \right)-1 V_{3_{j}}<v< V_{4} \\ -1 v\geq V_{4} \end{aligned} \right.$$

(36)

The actual value of reactive-power output is then:

$${Q_{j}}^{inv}\left( v \right)=q_{j}\left( v \right)Q_{j}^{rated}$$

(37)

To ensure electrical feasibility, inverter reactive-power operation is coupled to the available apparent-power capacity:

$${(P_{j}^{DG})}^{2}+{({Q_{j}}^{inv})}^{2}\leq{(S_{rated,j}^{DG})}^{2}$$

(38)

”

These updates provide a fully compliant Volt/VAR model that satisfies all capability, ordering, monotonicity, and protection-related requirements. In addition, the feeder ampacity constraint has been further explained and highlighted in the revised manuscript, as shown in Eq. (27):

| $\left\vert I_{s,L}^{K} \right\vert<I_{UL},$ ∀ *L*, ∀ *s,* ∀ *k* | (27) |
| --- | --- |

This explanation has been highlighted:

“

Eq. (27) constrains feeder currents to stay within the thermal ampacity, $I_{UL}$, of the conductors at every hour

”

**Authors’ action:** The smart-inverter modeling section has been strengthened to clearly present the complete detailed four-segment Volt/VAR formulation, including slope-based computation of internal breakpoints, enforced ordering and monotonicity, and coupling of reactive-power output to inverter apparent-power limits. Although all these feasibility conditions were already implemented in the original optimization code, they were not sufficiently detailed in the manuscript. The revised version now explicitly describes these mechanisms and includes complete mathematical expressions and constraints to ensure clarity and transparency.

**Reviewer #1, Concern #5: Uncertainty modeling is inconsistent: dependence among PV, wind, load, and EV processes is unspecified; scenario generation/reduction settings and retained probabilities are not documented; the PV surrogate ignores temperature and inverter clipping and exhibits unrealistic curvature at low irradiance.**

**Authors’ response:** We thank the reviewer for this important comment. We have substantially revised the manuscript to clarify the uncertainty modeling for PV, wind, load, and EV processes, the scenario-generation and reduction steps, and the PV surrogate model. The revised text now provides a complete and transparent description. Our detailed responses are provided below.

**Dependence Among PV, Wind, Load, and EV Processes**: We agree that the earlier text did not explicitly state how dependence among the uncertain variables is handled. In our study, all variables share the same hourly timeline, which induces natural dependence:

- PV output is driven by irradiance.
- Wind power is driven by wind speed.
- Load is scaled by the hourly base-load factor.
- EV demand profiles vary with time of day, arrival times, departure times, and state-of-charge (SOC) statistics.

Thus, PV, wind, load, and EV processes evolve jointly through shared hourly weather and behavioral patterns, providing implicit dependence without requiring an explicit multivariate covariance matrix. This approach is standard for feeder-level planning studies, where the goal is to evaluate operational limits under representative operating conditions rather than to construct full joint distributions. We have added the following clarification to the manuscript:

“

*PV, wind, load, and EV processes share the same hourly time index, reflecting realistic dependence driven by weather and time-of-day patterns. Within a given scenario, all processes evolve consistently without explicit covariance matrices; instead, dependence is captured implicitly through common driving profiles (irradiance, wind speed, base load, and EV charging behavior). This is consistent with standard distribution-system planning practice.*

”

**Scenario Generation and Reduction (1000 → 10 Scenarios):** We acknowledge the lack of detail in the previous version. The revised manuscript now specifies that:

- We first generated 1000 Monte Carlo trajectories using the probabilistic models described in Section 2 for each PV irradiance, wind speed, load scaling, EV arrival, departure, and SOC distributions (converted to aggregate EV load curves).
- A scenario-reduction procedure (forward selection based on trajectory distance) was applied to obtain 10 representative scenarios that preserve the variability and shape of the original ensemble. All retained scenarios were assigned equal probability, $\pi_{s}=\frac{1}{N_{s}}$.

To explain this part extensively, an illustrative figure, Fig. 2, has been presented; additionally, the following detailed explanations have been added in the revised manuscript:

“

The discussed stochastic schemes employ a probabilistic framework to handle load demand, EVs behaviour and numerous DG units output power by fitting historic hourly data to suitable probability distributions for parameter estimation. Using these assessed parameters, random variables could be produced according to their respective distributions across multiple scenarios to capture uncertainty. Scenario generation is performed via Monte Carlo simulation; however, due to the high computational burden of large-scale scenario sets, a scenario reduction strategy based on the Kantorovich Distance Matrix (KDM) is applied. This technique prunes redundant, similar scenarios while retaining a diverse and representative subset, reducing the original set to a manageable size. The KDM-based reduction process follows the following steps:

**Stage 1**: Produce the number of scenarios $\xi_{s}$(1,2,….,$N_{s}$), then estimate the distance among all scenarios $D(\xi_{s},\xi_{s^{'}})$=$\left| \xi_{s}-\xi_{s^{'}} \right|, s,s^{'}=1,2,\ldots..,N_{s}$ and set an initial probability to each scenario $\pi_{s}=\frac{1}{N_{s}}$. The initial probability for each scenario is calculated, in this step, simply by assuming that all generated scenarios are equally likely before any reduction is applied.

**Stage 2**: The algorithm identifies the two scenarios that are most similar to each other based on their distance. This can be formulated as: $D\left( s,s^{'} \right)$=$\min D\left( \xi_{s},\xi_{s^{'}} \right), s,s^{'}\in\xi_{s}$, $s\neq s^{'}.$

**Stage 3**: The algorithm chooses *s* as the one with the smaller distance calculated as $s=\pi_{s}\min D\left( \xi_{s},\xi_{s^{'}} \right)$, then remove the weaker scenario among the collection of scenarios, the updated set of scenarios becomes $S^{'}=S\backslash s$, the probability of the deleted scenario *s* is added to the closest remaining scenario $s^{'}$ as $\pi_{s}^{'}=\pi_{s}^{'}+\pi_{s}$.

**Stage 4**: Once the required number of reduced scenarios is reached, keep repeating Steps 2 and 3.

”

**PV Surrogate Model: Temperature, Clipping, and Low-Irradiance Curvature:** We acknowledge that our PV model is a simplified surrogate, chosen for computational efficiency within large-scale scenario evaluation. In our work, temperature derating is implicitly included by treating PV rated power as an effective derated capacity. Additionally, inverter clipping is enforced by the upper bound, which ensures AC-side clipping. This is illustrated through Eq. (30) in the revised manuscript: $0\leq P_{PV}\leq P_{PV}^{UL}$.

In addition, low-irradiance curvature has a negligible impact on hosting capacity because network constraints are driven by moderate–high generation hours, not hours where irradiance is < 150 W/m². To clarify these points, the following paragraph has been added to the revised manuscript in the related part:

“

PV output is modeled using a lightweight piecewise surrogate: quadratic for irradiance below $I_{c}$ W/m² and linear above, scaled by an effective rated power that embeds temperature and design derating. Clipping is enforced by the PV rated power bound. Since hosting-capacity limits occur mainly during high irradiance, the low-irradiance curvature has negligible effect on feeder-level hosting-capacity results. The framework can accommodate more detailed PV models if desired.

”

**EV Demand Modeling and Energy Consistency**: We clarified that this study adopts a feeder-level aggregate EV demand model rather than per-vehicle SOC dynamics, which is a valid modeling choice for feeder hosting-capacity studies. Specifically, arrival time, departure time, and SOC distributions are used to produce aggregate hourly EV demand trajectories for each of the 1000 initial Monte Carlo scenarios. These trajectories reflect typical dwell-time and energy requirements at the population level. The optimization does not schedule individual vehicles but instead coordinates smart-inverter DG and aggregate EV demand at each hour. To clarify these points, the following paragraph has been added to the revised manuscript:

“

*Individual EVs are not simulated; instead, aggregate hourly EV charging-demand trajectories are generated from probabilistic arrival, departure, and SOC distributions and included in the 1000 Monte Carlo samples. After scenario reduction, the optimization uses these aggregate EV profiles directly. This feeder-level modeling approach is appropriate for hosting-capacity assessment and avoids the need for per-vehicle SOC dynamics, which are not required for the scope of this study.*

”

**Authors’ action:** To address the reviewer’s concerns, substantial revisions have been made to clarify the uncertainty modeling framework. Explanatory paragraphs have been added to the relevant subsections to explicitly describe the implicit temporal dependence among PV, wind, load, and EV processes; the detailed scenario generation and reduction steps from 1000 Monte Carlo samples to 10 representative, equally probable scenarios; the justification for the PV surrogate model, including effective derating, clipping bounds, and the negligible impact of low-irradiance curvature; and the use of aggregate EV demand modeling appropriate for feeder-level analysis.

**Reviewer #1, Concern #6: Results introduce bias: solutions are filtered post hoc to keep only cases with losses below the base case, obscuring genuine trade-offs and affecting comparability.**

**Authors’ response:** We thank the reviewer for raising this point. We confirm that no post hoc or after-the-fact filtering of solutions was used in this study. At no stage were solutions discarded based on losses. Instead, the power-loss term is enforced within the optimization itself through the constraint:

$$P_{{loss}_{s}}^{k}<P_{loss}^{BC}, \forall s, \forall K$$

(28)

which is evaluated at every iteration of the optimizer. Any candidate solution that violates this bound is marked as infeasible by the optimizer and cannot be accepted or retained. Thus, the optimization process itself, not a post-processing step, ensures that loss levels remain within the allowable limits.

To ensure transparency, we have emphasized this constraint and its role in the revised manuscript. In addition, we have clarified the role of the normalized loss term, shown in Eq. (23), within the objective function and explicitly highlighted how it works together with the loss constraint to ensure consistent and unbiased treatment of losses during optimization.

$${PLI}_{s}^{k}=\frac{P_{{loss}_{s}}^{k}}{P_{loss}^{BC}}$$

(23)

In summary, the concern regarding post hoc filtering arises from a misunderstanding: loss limits are enforced internally by the optimization framework, and no external filtering or discarding of solutions is performed.

**Authors’ action:** We clarified in the revised manuscript that loss limits are enforced directly within the optimization through the built-in constraint $P_{{loss}_{s}}^{k}<P_{loss}^{BC}$, rather than through any post hoc filtering. This constraint is now explicitly highlighted in the related section. Additionally, we have clarified the role of the normalized loss term within the objective function and explicitly highlighted how it works together with the loss constraint to ensure consistent and unbiased treatment of losses during optimization.

**Reviewer #1, Concern #7:** **Optimization description is not reproducible: discrepancies exist between the narrative and equations for the “regeneration” step; constraint handling (penalties/repair/feasibility rules), stopping criteria, random seeds, number of runs, and statistical testing across runs are not reported.**

**Authors’ response:** We thank the reviewer for highlighting this point. In the revised manuscript, we have clarified and fully aligned the SFOA description with its mathematical implementation:

1. **Regeneration step:** As shown in Eq. (19), regeneration is applied only to the worst-ranked starfish using an exponential decay operator, consistent with the original SFOA formulation, rather than random reinitialization. Boundary constraints are enforced immediately after regeneration.
2. **Constraint handling:** In the revised manuscript, we clarify that hard constraints (bus voltages, active power loss, smart inverter constraints) are managed via a penalty-based approach. Any candidate solution violating one or more constraints is penalized by setting the hosting capacities terms (DG-HC, EV-HC) to zero and computing the objective with weighted penalty terms. It is worthy noting that, in the updated objective function formulation, the hosting capacities terms (DG-HC, EV-HC) do not appear in the denominator, eliminating any risk of division by zero when penalizing infeasible solutions.
3. **Stopping criterion, random seeds, and reproducibility:** Each optimization run uses *T_max_* iterations. To validate the SFOA’s performance, 10 independent runs were performed for hour 13 of scenario 5 (cases 2, 3, and 4) and compared with three other optimizers (Section 5.3). Based on this assessment, the SFOA was executed once per hour per scenario for all other analyses. Fixed random seeds were used for reproducibility of the assessment runs. These updates ensure consistency between the narrative, equations, and implementation, addressing reproducibility concerns.

To clarify these points, the following paragraphs have been added to the revised manuscript:

“

*In accordance with the original SFOA formulation explained in [35], the regeneration mechanism is applied only to the worst-ranked starfish (i = Np) in each iteration. Instead of full random reinitialization, regeneration is performed using an exponential decay operator that gradually attenuates the position vector as iterations progress, encouraging global exploration while maintaining stability.*

”

“

*Boundary constraints are enforced immediately after regeneration using the original SFOA boundary-repair rule. This ensures consistency between the narrative description, mathematical model, and implementation, enabling reproducibility. The SFOA was executed with population size Np = 50 and maximum iterations T_max_ = 300. The exploration and exploitation phases were performed as described, with the preying sub-process refining candidate positions and regeneration applied to the worst-ranked individual. Hard constraints, including bus voltage limits, feeder ampacity, total active power loss, inverter capacity limits, and inverter characteristic voltages ordering, are enforced using a penalty-based approach. Candidate solutions violating any of these constraints are assigned zero values for the hosting capacities terms (DG-HC and EV-HC), and the objective function is penalized accordingly. To validate optimizer performance, 10 independent runs were conducted for hour 13 of scenario 5 (cases 2, 3, and 4) and compared with three other optimizers (see Section 5.3). Based on this assessment, the SFOA was run once per hour per scenario for all other analyses. The stopping criterion was T_max_ iterations, with no early termination.*

”

**Authors’ action:** We revised the manuscript to ensure full reproducibility and clarity of the SFOA optimization procedure. The regeneration step is now described as an exponential decay operator applied only to the worst-ranked starfish, with boundary handling explicitly specified. Hard constraints (bus voltages, power loss, and inverter limits) are managed via a penalty-based approach, in which infeasible solutions are assigned zero hosting-capacity terms without causing division-by-zero errors. Algorithmic parameters, stopping criteria, random seeds, and the number of runs for the comparative assessment are explicitly stated. These updates align the narrative, equations, and implementation, ensuring that the optimization procedure is fully reproducible and transparent.

**Reviewer #1, Concern #8:** **Definitions and metrics for DG-HC and EV-HC permit gaming: instantaneous power-ratio indices (per hour) are used without arrival/dwell/energy consistency for EVs; voltage-deviation indices lack clear aggregation across scenarios and time and do not convey duration/probability.**

**Authors’ response:** Thank you for this important comment. We agree that metric definitions must prevent “trivial” solutions and clearly communicate performance across time and uncertainty. In the revised manuscript, we clarify the intent and interpretation of the hourly hosting indices, strengthen the EV hosting metric to ensure energy consistency, and add explicit aggregation and reporting across hours and scenarios (including duration and probability summaries).

1. EV-HC: enforcing energy consistency (avoiding gaming)

We agree that an instantaneous hourly power ratio alone can be gamed if it does not ensure that the demanded charging energy is actually delivered over the vehicle’s dwell time. To address this, we revised the EV hosting definition to be energy consistent. In particular, for each hour 𝑘 and scenario 𝑠, the delivered charging energy is defined as:

$${EV-HC}_{s}^{k}=\frac{\sum_{r=1}^{C} E_{{EV}_{s,r}}^{k}}{\sum_{r=1}^{C} E_{EV,r}^{rated}}$$

(21)

$$E_{{EV}_{s,r}}^{k}=P_{{EV}_{s,r}}^{k}\Delta t$$

(22)

2. DG-HC: power-based hosting is standard; we clarify scope and add an energy-based summary

We acknowledge the reviewer’s concern and clarify that DG hosting capacity in distribution networks is classically defined as the maximum instantaneous DG power injection that can be accommodated without violating network constraints (voltage limits, thermal limits, and losses). For this reason, DG-HC is formulated in our study as an hourly, scenario-specific, power-based hosting ratio (kW/kW). This aligns with established feeder hosting-capacity practice, where feasibility is evaluated per operating condition, and it matches the planning intent of our work, which is to assess admissible DG injection under each hour’s operational constraints. In addition, to avoid ambiguity in interpretation and to ensure a fully normalized metric, the DG-HC expression has been reformulated in the revised manuscript as the ratio between the allowable DG power injection and the aggregate rated capacity of all installed DG units. This normalization clarifies the metric, eliminates any possibility of “gaming,” and renders DG-HC fully comparable across scenarios and hours.

3. Voltage deviation index: aggregation across time/scenarios and probability/duration reporting

We thank the reviewer for highlighting the issue of aggregation and interpretability of voltage-deviation metrics. In our study, the voltage deviation index (DI) is defined at each hour $k$ and for each retained scenario $s$ as:

$${DI}_{s}^{k}=\sum_{i=1}^{N_{B}} \left| 1-V_{s,i}^{k} \right|, \forall s, \forall k$$

(24)

This formulation intentionally computes DI on an hourly and scenario-specific basis, because the 10 retained scenarios represent distinct deterministic operating conditions, each corresponding to a unique realization of EV behavior and load uncertainty after scenario reduction.

Thus, the reviewer’s concern about aggregation over time or probability is addressed as follows:

- **Scenario-specific evaluation avoids “gaming”**: By computing DI for every hour and for every scenario, the formulation prevents masking high-voltage deviations in certain hours by averaging them with other conditions. The optimization process is therefore exposed to the worst feasible deviations in any scenario–hour combination.
- **Hourly evaluation is consistent with operational constraints**: All operational constraints in the proposed formulation, including voltage limits, thermal loading, and power-loss boundaries, are enforced at every hour and for every scenario. Therefore, computing DI at each hour mirrors standard practice in feeder hosting-capacity assessment, where constraint violations cannot be averaged over time or scenarios.
- **Clarity and transparency**: The revised manuscript explicitly states that the 10 scenarios are handled as 10 deterministic operating conditions, and DI is evaluated independently for each condition. This provides a transparent framework where duration/probability concerns are handled through scenario probabilities, while voltage-feasibility concerns are handled through per-hour enforcement.

**Authors’ action:** In the revised manuscript, we clarified and strengthened the definitions and metrics to prevent potential gaming. The EV-HC metric was reformulated to enforce energy consistency by measuring delivered charging energy over the dwell period rather than relying solely on instantaneous power. The DG-HC metric was clarified as a standard hourly, power-based ratio and reformulated relative to the aggregate rated DG capacity to ensure normalization and comparability across scenarios and hours. The voltage deviation index (DI) was clarified to be calculated per hour and per scenario, with all operational constraints, including voltage limits, thermal loading, and power-loss boundaries, enforced on an hourly basis.

**Reviewer #1, Concern #9: Figures and tables are inconsistent: multiple plots appear as low-resolution screenshots with mismatched fonts and missing units; captions lack scenario/hour context; parameter tables mix solver knobs with physical limits and omit key network/penalty parameters needed for replication.**

**Authors’ response:** We thank the reviewer for these valuable observations. In the revised manuscript, all figures and tables have been carefully checked and updated where necessary. The figures have been regenerated at high resolution with consistent fonts, complete axis units, and improved visual clarity. We also confirm that none of the figures in either the original or the revised manuscript were created using screenshots; however, their formatting and resolution have been enhanced to ensure maximum clarity and consistency. Captions have been expanded to explicitly include the corresponding scenario, hour, and case.

Similarly, the tables have been reorganized to clearly separate physical network parameters, optimization settings, and scenario or simulation information. Missing reproducibility-related parameters, including penalty settings, normalization references, and random-seed information, have been added.

With these revisions, all figure- and table-related concerns raised by the reviewer have been fully addressed in the updated manuscript.

**Authors’ action:** The figures were regenerated with enhanced resolution and consistent formatting, captions were expanded, and tables were reorganized and supplemented with missing parameters. We also verified that no figures were based on screenshots.

**Reviewer #1, Concern #10:** **Unnecessary and misleading graphics (Star fish image) are included: several image choices and stylistic elements evoke biological themes and distract from an energy-systems contribution, creating confusion at first glance about the paper’s domain. They must be removed.**

**Authors’ response:** We appreciate the reviewer’s observation regarding misleading graphics. Figure 3, which previously illustrated the “Exploration phase of SFOA,” has been removed because its stylistic elements evoked biological themes and could distract from the energy-systems focus of the paper, potentially causing confusion about the study’s domain. In addition, Figure 5 (“Volt/VAR operational curve of the smart inverter”) has also been removed as part of streamlining the visual content. The manuscript now includes only domain-appropriate and technically essential figures to maintain clarity and focus.

**Authors’ action:** Figure 3 and Figure 5 have been completely removed from the manuscript. All remaining figures were reviewed to ensure they are technically relevant and aligned with the energy-systems domain.

**Reviewer #1, Concern #11: Conclusions are qualitative and restate trends without precise numerical deltas, sensitivity ranges, or explicit limitations, weakening the evidential value of the study’s claims.**

**Authors’ response:** We thank the reviewer for this important observation. In the revised manuscript, we have substantially strengthened Section 6.4 (Key Findings and Implications) to provide precise numerical deltas, explicitly report sensitivity ranges, and discuss practical limitations, thereby fully addressing the reviewer’s concern. Specifically:

**Quantitative deltas:** The conclusions now include explicit improvements in DG installed capacity and EV charging demand for both test systems. For the Cairo 59-bus distribution system (DS), coordinated EV charging increases DG capacity from 34.6 MW to 41.7 MW and EV demand from 23.6 MWh to 83.4 MWh; when integrated with smart-inverter (SI) Volt/VAR control, these values further improve to 49.6 MW and 89.1 MWh, respectively. For the IEEE 33-bus DS, DG capacity rises from 5.5 MW to 7 MW and EV demand from 1.18 MWh to 3.2 MWh, increasing further to 8.7 MW and 4.59 MWh with SI coordination. Voltage deviations are also quantitatively reported; for example, the mean voltage-deviation index in the IEEE 33-bus system decreases from 50.3% to 29.8%. These precise figures provide direct evidence supporting the claimed improvements.

**Sensitivity ranges:** An extensive sensitivity analysis was performed on the IEEE 33-bus system at hour 13 under scenario 5 across eight distinct weighting-factor combinations, with all results reported in Table 2 of the revised manuscript. This analysis reveals that DG installed capacity ranges between 7.27 MW and 18.89 MW, and EV charging demand between 2.03 MWh and 5.42 MWh, depending on the selected priority settings. Only a limited subset of weighting configurations satisfies all operational constraints, clearly demonstrating the sensitivity of system performance to the selection of objective-function weights.

- **Justification of chosen weights:** Based on the sensitivity analysis, the weighting set (0.3, 0.3, 0.1, 0.3) was selected because it lies within the feasible range, avoids constraint violations observed in hosting- or priority-biased cases, and achieves the best trade-off between DG and EV hosting capacity, voltage regulation, and reliability. Compared with the original weighting set (0.35, 0.35, 0.15, 0.15), the revised configuration better aligns with the updated objectives, avoids excessive penalization of the reliability term, and yields a more stable and operationally feasible solution. The sensitivity-analysis results added to the revised manuscript are summarized in Table 2.
- **Explicit limitations:** The conclusions now explicitly acknowledge that the proposed framework assumes idealized DSO coordination, and that practical deployment may face challenges such as communication delays, partial observability, and limited controllability of distributed resources. These limitations may reduce the realized DG-HC and EV-HC, affect voltage regulation, or increase losses, and are clearly linked to the reported numerical results. Future work is proposed to mitigate these limitations through hierarchical or decentralized control strategies and the integration of additional mechanisms such as battery energy storage systems (BESS), vehicle-to-grid (V2G), and active power curtailment.

Here are the sensitivity analysis results that have been added to the revised manuscript:

Table 2: Sensitivity analysis of weighting-coefficient combinations and their impact on the optimization results

| Combination number | Weighting factors | | | | OF | DG installed capacity  (MW) | EV charging demand  (MWh) | Vmin  (pu) | Vmax  (pu) | Ploss  (kW) | Constraints violation |
| --- | --- | --- | --- | --- | --- | --- | --- | --- | --- | --- | --- |
|  | $w_{1}$ | $w_{2}$ | $w_{3}$ | $w_{4}$ |  |  |  |  |  |  |  |
| Hosting-biased | 0.5 | 0.5 | 0 | 0 | 0.4396 | 15.53 | 5.23 | 0.9519 | 1.0037 | 265.1813 | Ploss violation |
| Hosting-biased, with little attention to other objectives | 0.4 | 0.4 | 0.1 | 0.1 | 0.4779 | 15.83 | 5.12 | 0.9639 | 1.0049 | 265.1569 | Ploss violation |
| Linearly decreasing weights | 0.4 | 0.3 | 0.2 | 0.1 | 0.4789 | 16.624 | 4.8685 | 0.9758 | 1.0083 | 265.4637 | Ploss violation |
| Linearly increasing weights | 0.1 | 0.2 | 0.3 | 0.4 | 0.4490 | 7.2698 | 2.0271 | 0.9866 | 1.0028 | 129.8056 | All are acceptable |
| DG priority | 0.6 | 0.2 | 0.1 | 0.1 | 0.5855 | 18.89 | 4.4920 | 0.9797 | 1.0062 | 264.8514 | Ploss violation |
| EV priority | 0.2 | 0.6 | 0.1 | 0.1 | 0.3459 | 12.088 | 5.42 | 0.9541 | 1.0027 | 264.7684 | Ploss violation |
| Base | 0.35 | 0.35 | 0.15 | 0.15 | 0.4930 | 15.47 | 5.08 | 0.9684 | 1.0063 | 265.0483 | Ploss violation |
| **Proposed** | 0.3 | 0.3 | 0.1 | 0.3 | 0.5669 | 11.997 | 4.49 | 0.9757 | 1.0028 | 203.696 | All are acceptable |

**Authors’ action:** Section 6.4 has been revised to include precise numerical deltas for DG installed capacity, EV charging demand, and voltage improvements, as well as a sensitivity analysis at hour 13 under scenario 5 showing DG ranges of 7.27–18.89 MW and EV ranges of 2.03–5.42 MWh. The selected weighting set (0.3, 0.3, 0.1, 0.3) is justified as achieving the best balance between hosting-capacity enhancement, voltage regulation, and reliability while avoiding constraint violations. Practical limitations, including communication delays and limited controllability, are now explicitly discussed in relation to the results, ensuring that the conclusions are quantitative, robust, and evidence-based.

**Reviewer #2, Concern #1: regarding the setting of weighting coefficients in the multi-objective optimization, it is suggested that the authors include a necessary sensitivity analysis. Please specifically illustrate the potential impact on the optimization results when the weighting priorities change, thereby demonstrating the robustness of the current parameter selection and avoiding the limitations of relying solely on empirical selection.**

**Authors’ response:** We sincerely thank the reviewer for highlighting the importance of validating the weighting-coefficient selection in the multi-objective optimization. In response, we conducted a comprehensive sensitivity analysis using eight representative weight combinations, including hosting-biased, DG-priority, EV-priority, and linearly increasing and decreasing distributions, evaluated at hour 13 under scenario 5. The full results have been added to the revised manuscript (Table 2). The analysis reveals that several combinations that heavily prioritize hosting capacity (e.g., 0.5–0.5–0–0 and 0.4–0.4–0.1–0.1) violate the power-loss constraint, indicating a lack of operational feasibility. Priority-based combinations (DG- or EV-dominated) also lead to higher losses or increased voltage deviations. Although the linearly increasing weighting set (0.1–0.2–0.3–0.4) satisfies all constraints, it significantly suppresses both DG and EV hosting capacity due to overemphasis on the reliability component. The proposed weighting set (0.3, 0.3, 0.1, 0.3), derived after revising the objective-function formulation, provides the best overall trade-off. It satisfies all operational constraints, maintains a safe voltage range (0.9757–1.0028 pu), minimizes power loss (203.696 kW), and preserves competitive DG and EV hosting capacities.

Importantly, the reviewer’s suggestion enabled us to demonstrate that the new weighting configuration also improves upon the original manuscript’s weighting set (0.35, 0.35, 0.15, 0.15). The revised weights better align with the updated objective-function formulation, reduce excessive penalization of the reliability term, and yield a more stable and operationally feasible solution. Accordingly, the revised manuscript now includes a complete sensitivity analysis, a detailed discussion of the observed trends, and a clear justification for the adopted weighting coefficients.

**Authors’ action:** Section 6.4 has been revised to include precise numerical deltas for DG installed capacity, EV charging demand, and voltage improvements, as well as a sensitivity analysis at hour 13 under scenario 5 showing DG ranges of 7.27–18.89 MW and EV ranges of 2.03–5.42 MWh. The selected weighting set (0.3, 0.3, 0.1, 0.3) is justified as achieving the best balance between hosting-capacity enhancement, voltage regulation, and reliability while avoiding constraint violations. Practical limitations, including communication delays and limited controllability, are now explicitly discussed in relation to the results, ensuring that the conclusions are quantitative, robust, and evidence-based.

Table 2: Sensitivity analysis of weighting-coefficient combinations and their impact on the optimization results

| Combination number | Weighting factors | | | | OF | DG installed capacity  (MW) | EV charging demand  (MWh) | Vmin  (pu) | Vmax  (pu) | Ploss  (kW) | Constraints violation |
| --- | --- | --- | --- | --- | --- | --- | --- | --- | --- | --- | --- |
|  | $w_{1}$ | $w_{2}$ | $w_{3}$ | $w_{4}$ |  |  |  |  |  |  |  |
| Hosting-biased | 0.5 | 0.5 | 0 | 0 | 0.4396 | 15.53 | 5.23 | 0.9519 | 1.0037 | 265.1813 | Ploss violation |
| Hosting-biased, with little attention to other objectives | 0.4 | 0.4 | 0.1 | 0.1 | 0.4779 | 15.83 | 5.12 | 0.9639 | 1.0049 | 265.1569 | Ploss violation |
| Linearly decreasing weights | 0.4 | 0.3 | 0.2 | 0.1 | 0.4789 | 16.624 | 4.8685 | 0.9758 | 1.0083 | 265.4637 | Ploss violation |
| Linearly increasing weights | 0.1 | 0.2 | 0.3 | 0.4 | 0.4490 | 7.2698 | 2.0271 | 0.9866 | 1.0028 | 129.8056 | All are acceptable |
| DG priority | 0.6 | 0.2 | 0.1 | 0.1 | 0.5855 | 18.89 | 4.4920 | 0.9797 | 1.0062 | 264.8514 | Ploss violation |
| EV priority | 0.2 | 0.6 | 0.1 | 0.1 | 0.3459 | 12.088 | 5.42 | 0.9541 | 1.0027 | 264.7684 | Ploss violation |
| Base | 0.35 | 0.35 | 0.15 | 0.15 | 0.4930 | 15.47 | 5.08 | 0.9684 | 1.0063 | 265.0483 | Ploss violation |
| **Proposed** | 0.3 | 0.3 | 0.1 | 0.3 | 0.5669 | 11.997 | 4.49 | 0.9757 | 1.0028 | 203.696 | All are acceptable |

**Reviewer #2, Concern #2: Second, in terms of algorithm comparison, although the manuscript demonstrates the advantage of SFOA in convergence accuracy, the dimensions of comparison appear somewhat limited. It is recommended to supplement the study with a theoretical analysis of computational complexity using Big-O notation to more comprehensively reflect the differences in algorithmic efficiency.**

**Authors’ response:** We thank the reviewer for this suggestion. A complete computational-complexity analysis has now been added to the revised manuscript (Section 5.3). For an optimizer with population size $N_{p}$, maximum number of iterations $T_{\max}$, and decision-space dimension $D$, the per-iteration complexity of SFOA is dominated by objective-function evaluations. Each evaluation requires one Newton–Raphson load-flow computation and a full set of voltage, loss, inverter, and hosting-capacity constraint checks. The evaluation cost is $C_{\text{evaluate}}=O(N_{b}+N_{l}+N_{DG}+N_{EV})$, where $N_{b}$, $N_{l}$, $N_{DG}$, and $N_{EV}$ denote the numbers of buses, lines, DG units, and EV charging units, respectively. Accordingly, the overall computational complexity of SFOA is:

$$O(N_{p}\text{ }T_{max}\text{ }(D+C_{\text{evaluate}}))=O(N_{p}\text{ }T\text{ }(D+N_{b}+N_{l}+N_{DG}+N_{EV})).$$

This complexity is comparable to that of ZOA [41], GWO [42], and RUN [43]; however, SFOA benefits from fewer hyperparameters and reduced per-iteration overhead, which supports the favorable efficiency observed in the comparative assessment. Since each hour and scenario is solved independently, the 24-hour, 10-scenario study comprises 240 embarrassingly parallel optimization runs. These details are now explicitly included in the revised manuscript, complementing the empirical comparisons presented in Section 5.3.

“

*In addition to empirical performance assessment, the computational complexity of SFOA is analyzed. Let* $N_{p}$ *denote the population size,* $T_{max}$ *the number of iterations, and* $D$ *the dimensionality of the decision vector. The dominant computational cost arises from objective-function evaluation, including one Newton-Raphson load-flow computation and checking all voltage, power-loss, inverter, and hosting-capacity constraints. For a network with* $N_{b}$ *buses,* $N_{l}$ *lines,* $N_{DG}$ *DG units, and* $N_{EV}$ *EV charging stations, the evaluation cost is:*

$$C_{\text{evaluate}}=O(N_{b}+N_{l}+N_{DG}+N_{EV})$$

(40)

*Consequently, the overall complexity of SFOA is:*

$$O(N_{p}\text{ }T\text{ }(D+C_{\text{evaluate}}))=O(N_{p}\text{ }T\text{ }(D+N_{b}+N_{l}+N_{DG}+N_{EV}))$$

(41)

*This complexity is comparable to the ZOA, GWO, and RUN, but SFOA benefits from fewer hyperparameters and reduced per-iteration overhead, supporting the efficiency observed in the convergence comparisons. Each hour and scenario is solved independently, resulting in 240 parallelizable optimization runs across the 24-hour, 10-scenario study.*

”

**Authors’ action:** A complete computational-complexity analysis of SFOA has been added to the revised manuscript (Section 5.3). The analysis explicitly quantifies the Big-O complexity in terms of population size, iteration count, decision-space dimension, and network evaluation cost, and compares it with ZOA, GWO, and RUN. This addition complements the empirical convergence results, clarifies the algorithm’s efficiency relative to competing metaheuristics, and demonstrates that both theoretical and practical considerations support the observed performance advantage of SFOA.

**Reviewer #2, Concern #3: Finally, the current model construction relies on the ideal assumption that the Distribution System Operator DSO can perfectly coordinate all units. To enhance the practical engineering value of the paper, it is suggested to appropriately supplement the discussion section with an analysis of challenges in actual deployment, such as the choice of communication architecture or the potential impact of signal delays on control effectiveness.**

**Authors’ response:** We thank the reviewer for raising this important point regarding the practical deployment of the proposed coordination framework. In the original study, we assumed idealized DSO control with instantaneous and perfect coordination of all DG units, EV charging stations, and smart inverters. While this assumption allows for a clear evaluation of algorithmic performance and hosting-capacity enhancement, we acknowledge that real-world implementation would face practical constraints. Specifically, reliable communication between the DSO and field devices is essential, and factors such as signal latency, packet loss, and measurement noise can reduce the effectiveness of Volt/VAR control and EV scheduling. In addition, partial observability or limited controllability of distributed resources may prevent fully centralized coordination.

To address this concern, we have added a discussion in the revised manuscript highlighting these limitations and suggesting that future research could extend the proposed framework by incorporating hierarchical or decentralized control architectures, robust delay-compensation mechanisms, and fault-tolerant strategies. These additions emphasize the practical engineering relevance of the work while clarifying the assumptions made in the current study.

The following paragraph has been added to the revised manuscript:

“

*Although the proposed framework demonstrates substantial improvements under idealized DSO coordination, practical deployment may face limitations such as communication delays, partial observability, and limited controllability of distributed resources. These factors could reduce the realized DG and EV hosting capacities, affect voltage regulation, or increase power losses.*

”

**Authors’ action:** A discussion was added to the revised manuscript to acknowledge the assumption of idealized DSO coordination and to highlight practical deployment challenges, including communication delays, partial observability, and limited controllability of distributed resources. Potential future extensions involving hierarchical or decentralized control architectures and delay-robust strategies are also briefly discussed to enhance the practical relevance of the study.

**Reviewer #3, Concern #1: The abstract and introduction are not in research-article form. The abstract should open with the core challenge, state the proposed coordination method and decision variables, and report key quantitative outcomes. The introduction reads like a report and lacks a tight three-item contribution statement and a critical comparison to recent scenario-based multi-energy scheduling**

**Authors’ response:** We thank the reviewer for the feedback regarding the abstract and the Introduction. The abstract has been revised to follow a clear research-article arc, emphasizing the problem, the proposed method, and key quantitative outcomes in a concise manner. The updated abstract is as follows:

“

*The rapid growth of renewable-based distributed generation (DG) and electric vehicles (EVs) poses significant operational challenges for distribution systems (DSs), particularly under uncertainties in renewable output, load demand, and EV charging behavior. Distribution system operators must therefore evaluate and enhance both DG hosting capacity (DG-HC) and EV hosting capacity (EV-HC) while maintaining voltage security and reducing losses. This study presents a stochastic, multi-objective optimization framework that jointly coordinates smart inverter (SI)-based Volt/VAR control and EV charging scheduling to simultaneously maximize DG-HC and EV-HC and minimize active power losses and voltage deviation. The framework integrates active power management through EV charging coordination and reactive power support via optimally deployed SIs. The resulting multi-objective problem is solved using the Starfish Optimization Algorithm (SFOA) and benchmarked against three established metaheuristics. The methodology is validated on the IEEE 33-bus system and a real 59-bus distribution network in Cairo, Egypt. Results show that coordinated SI–EV control increases DG-HC and EV-HC by up to 74% and 89%, respectively, and achieves voltage deviation reductions of 55% in the IEEE 33-bus system and 11% in the Cairo DS. Comparative analysis confirms that SFOA provides superior convergence and solution quality relative to the competing techniques.*

”

In addition, the Introduction section has been substantially expanded and now includes 27 updated references. Its writing style has been enhanced and revised to improve technical clarity and readability. These changes provide a stronger and more focused context for the study and ensure up-to-date coverage of multi-objective HC assessment, coordinated SI–EV scheduling, and uncertainty-aware optimization. Table 1 has also been considerably expanded to compare contemporary works across multiple technical dimensions, including uncertainty modeling, coordinated active/reactive power management, and hosting-capacity-oriented objectives, thus providing a clearer positioning of this study’s novelty. Here is the updated version of Table 1:

Table 1. Overview of the key contributions of this study in comparison with selected works from the literature.

| Assessment Parameters | | [10], [16] | [11],[15] | [14] | [18] | [19]–[21] | [23] | [22] | [25] | [4] | [13] | [26] | [8] | This work |
| --- | --- | --- | --- | --- | --- | --- | --- | --- | --- | --- | --- | --- | --- | --- |
| HC evaluation | | 𝕐 | 𝕐 | 𝕐 | ℕ | 𝕐 | 𝕐 | 𝕐 | ℕ | 𝕐 | 𝕐 | 𝕐 | 𝕐 | 𝕐 |
| System uncertainties | | 𝕐 | 𝕐 | 𝕐 | ℕ | ℕ | 𝕐 | ℕ | 𝕐 | ℕ | ℕ | ℕ | ℕ | 𝕐 |
| DG technology variety | | ℕ | ℕ | ℕ | ℕ | ℕ | ℕ | ℕ | 𝕐 | ℕ | ℕ | 𝕐 | 𝕐 | 𝕐 |
| Active power support | | 𝕐 | 𝕐 | 𝕐 | ℕ | 𝕐 | 𝕐 | 𝕐 | 𝕐 | 𝕐 | 𝕐 | 𝕐 | 𝕐 | 𝕐 |
| Reactive power support | | ℕ | 𝕐 | ℕ | 𝕐 | ℕ | 𝕐 | 𝕐 | ℕ | ℕ | 𝕐 | ℕ | 𝕐 | 𝕐 |
| EVs charging scheduling | | 𝕐 | 𝕐 | 𝕐 | ℕ | ℕ | ℕ | ℕ | 𝕐 | 𝕐 | 𝕐 | 𝕐 | 𝕐 | 𝕐 |
| Optimization objectives | DG-HC maximization | 𝕐 | 𝕐 | 𝕐 | ℕ | 𝕐 | 𝕐 | 𝕐 | ℕ | 𝕐 | 𝕐 | 𝕐 | 𝕐 | 𝕐 |
|  | EV-HC maximization | ℕ | ℕ | ℕ | ℕ | ℕ | ℕ | ℕ | 𝕐 | 𝕐 | ℕ | 𝕐 | 𝕐 | 𝕐 |
|  | Loss reduction | 𝕐 | ℕ | ℕ | ℕ | ℕ | ℕ | ℕ | ℕ | ℕ | 𝕐 | 𝕐 | 𝕐 | 𝕐 |
|  | Voltage profile enhancement | ℕ | ℕ | ℕ | 𝕐 | ℕ | 𝕐 | ℕ | ℕ | ℕ | ℕ | ℕ | ℕ | 𝕐 |

**Authors’ action:** The abstract has been fully revised to follow a clear research-article structure, emphasizing the core problem, the proposed coordination framework, and key quantitative outcomes. The Introduction has been substantially expanded and rewritten to improve technical clarity, remove verbose bullet lists, and provide a focused discussion of the relevant literature. A total of 27 updated references have been included to ensure comprehensive coverage of multi-objective hosting capacity assessment, coordinated SI–EV scheduling, and uncertainty-aware optimization, thereby strengthening the context and contribution of the study. Also, many other changes have been made in response to the other comments.

**Reviewer #3, Concern #2: The optimization is ill posed. The objective uses reciprocals of hosting-capacity indices, the terms are not scaled to a common magnitude, and there is no explicit aggregation over hours and scenarios. A post hoc loss filter further biases the target being minimized.**

**Authors’ response:** We thank the reviewer for this insightful comment. In the revised manuscript, the multi-objective optimization framework has been substantially reformulated to ensure numerical stability, proper scaling, and full clarity regarding the role of hours and scenarios.

**Removal of all reciprocal terms**

We fully agree that the use of $1/HC$ in the original objective-function formulation could lead to numerical instability when hosting-capacity values become small. In the revised work, all reciprocal terms have been removed. Additionally, hosting-capacity indices are now used in the form $\left( 1 - HC \right)$, which is bounded, smooth, and numerically stable on $\left[ 0 , 1 \right]$. This eliminates the ill-posedness flagged initially by the reviewer. The modified objective function becomes:

$$minof=\min\{w_{1}\left( 1-{DG-HC}_{s}^{k} \right)+w_{2}\left( 1-{EV-HC}_{s}^{k} \right)++w_{3}{(DI}_{s}^{k}/{DI}_{BC})+w_{4}{(PLI}_{s}^{k})\}, \forall s, \forall k$$

(25)

**Revised and normalized definitions of DG-HC and EV-HC**

$${DG-HC}_{s}^{k}=\frac{\sum_{j=1}^{N} {P_{{WT}_{s,j}}}^{k}+ \sum_{j=1}^{M} {P_{{PV}_{s,j}}}^{k}}{\sum_{j=1}^{N} P_{WT,j}^{rated}+ \sum_{j=1}^{M} P_{PV,j}^{rated}}$$

(20)

$${EV-HC}_{s}^{k}=\frac{\sum_{r=1}^{C} E_{{EV}_{s,r}}^{k}}{\sum_{r=1}^{C} E_{EV,r}^{rated}}$$

(21)

This formulation ensures that both indices lie strictly in [0,1]. Both are normalized using physically meaningful upper bounds (installed DG capacity and EV charger ratings). No variable can be artificially inflated (“gamed”), eliminating the technical weakness noted by the reviewer.

**Explicit clarification of scenario handling and time resolution**

We revised the manuscript to clearly explain that the optimization is not a single stochastic expected-value problem, instead, each reduced scenario $s$ and each hour $k$ represent a different operating condition under uncertainty. Thus, a separate deterministic optimization problem is solved independently for each pair $(k,s)$. Therefore, no averaging or aggregation across hours or scenarios is missing; the formulation is intentionally scenario-wise and hour-wise to obtain detailed hourly hosting-capacity profiles.

**Re-running all simulations with the corrected objective function**

Because the corrected objective function materially changes the optimization landscape, all experiments and results have been recomputed using the revised, well-posed formulation. The updated numerical results now reflect the corrected hosting-capacity definitions and the stable objective structure.

Therefore, the optimization framework in the revised manuscript is now mathematically well posed, free of reciprocal and unbounded terms, fully normalized, explicitly scenario-wise and hour-wise, and consistent with hosting-capacity practices in the literature.

Finally: Regarding loss filtering: We thank the reviewer for raising this point. We confirm that no post hoc or after-the-fact filtering of solutions was used in this study. At no stage were solutions discarded based on losses. Instead, the power-loss term is enforced within the optimization itself through the constraint:

$$P_{{loss}_{s}}^{k}<P_{loss}^{BC}, \forall s, \forall k$$

(28)

which is evaluated at every iteration of the optimizer. Any candidate solution that violates this bound is marked as infeasible by the optimizer and cannot be accepted or retained. Thus, the optimization process itself, not a post-processing step, ensures that loss levels remain within the allowable limits.

To ensure transparency, we have emphasized this constraint and its role in the revised manuscript. In addition, we have clarified the role of the normalized loss term, shown in Eq. (23), within the objective function and explicitly highlighted how it works together with the loss constraint to ensure consistent and unbiased treatment of losses during optimization.

$${PLI}_{s}^{k}=\frac{P_{{loss}_{s}}^{k}}{P_{loss}^{BC}}$$

(23)

In summary, the concern regarding post hoc filtering arises from a misunderstanding: loss limits are enforced internally by the optimization framework, and no external filtering or discarding of solutions is performed.

**Authors’ action:** In response to the reviewer’s observation, we have completely reformulated the multi-objective function to ensure mathematical well-posedness and eliminate ambiguity. Precisely, (i) all reciprocal hosting-capacity terms were removed and replaced with bounded, normalized expressions using $1-HC$, eliminating numerical instability; (ii) DG-HC and EV-HC were redefined as normalized, capacity-based indices bounded in $[0,1]$; and (iii) scenario and hour treatment was explicitly clarified, showing that each scenario–hour pair is optimized independently rather than aggregated, thereby removing ambiguity regarding minimization across time and scenarios. The multi-objective function formulation subsection has been entirely rewritten, and all results were regenerated using the corrected, well-posed formulation. We clarified in the revised manuscript that loss limits are enforced directly within the optimization through the built-in constraint $P_{{loss}_{s}}^{k}<P_{loss}^{BC}$, rather than through any post hoc filtering. This constraint is now explicitly highlighted in the related section. Additionally, we have clarified the role of the normalized loss term within the objective function and explicitly highlighted how it works together with the loss constraint to ensure consistent and unbiased treatment of losses during optimization.

**Reviewer #3, Concern #3: Electrical feasibility is incomplete. Volt/VAR points are not tied to inverter apparent-power capability, step or rate limits are not stated, and the four-point curve lacks ordering and monotonicity checks. Feeder ampacity and protection assumptions are not fully enumerated.**

**Authors’ response:** We thank the reviewer for highlighting the need for a more rigorous and electrically consistent formulation of the smart-inverter Volt/VAR model. In the revised manuscript, the Volt/VAR characteristic is now fully specified with explicit mathematical expressions for the four-point curve, including ordered breakpoints, negative slopes, and a guaranteed monotonic, piecewise-affine profile. The intermediate points $V_{2j}$ and $V_{3j}$ are computed directly from the decision-variable slopes $m_{1j}$ and $m_{2j}$, ensuring consistent deadband behavior. Although the original code fully enforced Volt/VAR monotonicity, breakpoint ordering, and inverter apparent-power capability limits, these implementation details were not sufficiently highlighted in the previous manuscript.

The revised version now explains these mechanisms explicitly and provides the complete mathematical formulation to reflect the model already used in the simulations. These explanations have been added to the revised manuscript in the Smart Inverter Modeling subsection:

“

*To ensure electrical feasibility, this work adopts an explicit, capability-consistent formulation of the four-segment Volt/VAR characteristic, with constraints guaranteeing monotonicity, correct ordering of curve points, and compliance with inverter apparent-power limits. Each inverter* $j$ *uses a fixed low-voltage saturation point* $V_{1}=0.91\text{ pu}$ *and the high-voltage saturation point* $V_{4}=1.04\text{ }\text{ }\text{pu}$*. Two decision variables define the slopes in the lower and upper regions:*

$$m_{1}\leq0 , m_{2}\leq0$$

(32)

*The intermediate breakpoints* $V_{2j}$ *and* $V_{3j}$ *are not free variables; they are deterministically computed from the slopes:*

$$V_{2_{j}}=\frac{-1}{m_{1_{j}}}+V_{1}$$

(33)

$$V_{3_{j}}=\frac{1}{m_{2_{j}}}+V_{4}$$

(34)

*These expressions guarantee that the deadband width adjusts consistently with the selected slopes. Ordering and monotonicity are enforced through the explicit constraints implemented via penalty terms in the optimization to prevent infeasible curve shapes.*

$$V_{1}<V_{2_{j}}<V_{3_{j}}<V_{4}$$

(35)

The per-unit reactive power as a function of the predefined voltage at the PCC $q_{j}(v)$ is defined through a continuous piecewise-affine Volt/VAR characteristic:

$$q_{j}\left( v \right)=\left\{ \begin{aligned} 1 v\leq V_{1} \\ m_{1_{j}}\left( v-V_{1} \right)+1 V_{1}<v<V_{2_{j}} \\ 0 V_{2_{j}}<v<V_{3_{j}} \\ m_{2_{j}}\left( v-V_{4} \right)-1 V_{3_{j}}<v< V_{4} \\ -1 v\geq V_{4} \end{aligned} \right.$$

(36)

The actual value of reactive-power output is then:

$${Q_{j}}^{inv}\left( v \right)=q_{j}\left( v \right)Q_{j}^{rated}$$

(37)

To ensure electrical feasibility, inverter reactive-power operation is coupled to the available apparent-power capacity:

$${(P_{j}^{DG})}^{2}+{({Q_{j}}^{inv})}^{2}\leq{(S_{rated,j}^{DG})}^{2}$$

(38)

”

These updates provide a fully compliant Volt/VAR model that satisfies all capability, ordering, monotonicity, and protection-related requirements. In addition, the feeder ampacity constraint has been further explained and highlighted in the revised manuscript, as shown in Eq. (27):

| $\left\vert I_{s,L}^{K} \right\vert<I_{UL},$ ∀ *L*, ∀ *s,* ∀ *k* | (27) |
| --- | --- |

This explanation has been highlighted:

“

Eq. (27) constrains feeder currents to stay within the thermal ampacity, $I_{UL}$, of the conductors at every hour

”

**Authors’ action:** The smart-inverter modeling section has been strengthened to clearly present the complete detailed four-segment Volt/VAR formulation, including slope-based computation of internal breakpoints, enforced ordering and monotonicity, and coupling of reactive-power output to inverter apparent-power limits. Although all these feasibility conditions were already implemented in the original optimization code, they were not sufficiently detailed in the manuscript. The revised version now explicitly describes these mechanisms and includes complete mathematical expressions and constraints to ensure clarity and transparency.

**Reviewer #3, Concern #4: Uncertainty modeling is inconsistent. The PV surrogate ignores temperature and clipping and is nonstandard at low irradiance. Dependence among PV, wind, load, and EV is not justified. Scenario generation and reduction settings and retained probabilities are not reported. The Weibull CDF is correct but the text confuses the scale and shape parameter names.**

**Authors’ response:** We thank the reviewer for this important comment. We have substantially revised the manuscript to clarify the uncertainty modeling for PV, wind, load, and EV processes, the scenario-generation and reduction steps, and the PV surrogate model. The revised text now provides a complete and transparent description. Our detailed responses are provided below.

**Dependence Among PV, Wind, Load, and EV Processes**: We agree that the earlier text did not explicitly state how dependence among the uncertain variables is handled. In our study, all variables share the same hourly timeline, which induces natural dependence:

- PV output is driven by irradiance.
- Wind power is driven by wind speed.
- Load is scaled by the hourly base-load factor.
- EV demand profiles vary with time of day, arrival times, departure times, and state-of-charge (SOC) statistics.

Thus, PV, wind, load, and EV processes evolve jointly through shared hourly weather and behavioral patterns, providing implicit dependence without requiring an explicit multivariate covariance matrix. This approach is standard for feeder-level planning studies, where the goal is to evaluate operational limits under representative operating conditions rather than to construct full joint distributions. We have added the following clarification to the manuscript:

“

*PV, wind, load, and EV processes share the same hourly time index, reflecting realistic dependence driven by weather and time-of-day patterns. Within a given scenario, all processes evolve consistently without explicit covariance matrices; instead, dependence is captured implicitly through common driving profiles (irradiance, wind speed, base load, and EV charging behavior). This is consistent with standard distribution-system planning practice.*

”

**Scenario Generation and Reduction (1000 → 10 Scenarios):** We acknowledge the lack of detail in the previous version. The revised manuscript now specifies that:

- We first generated 1000 Monte Carlo trajectories using the probabilistic models described in Section 2 for each PV irradiance, wind speed, load scaling, EV arrival, departure, and SOC distributions (converted to aggregate EV load curves).
- A scenario-reduction procedure (forward selection based on trajectory distance) was applied to obtain 10 representative scenarios that preserve the variability and shape of the original ensemble. All retained scenarios were assigned equal probability, $\pi_{s}=\frac{1}{N_{s}}$.

To explain this part extensively, an illustrative figure, Fig. 2, has been presented; additionally, the following detailed explanations have been added in the revised manuscript:

“

The discussed stochastic schemes employ a probabilistic framework to handle load demand, EVs behaviour and numerous DG units output power by fitting historic hourly data to suitable probability distributions for parameter estimation. Using these assessed parameters, random variables could be produced according to their respective distributions across multiple scenarios to capture uncertainty. Scenario generation is performed via Monte Carlo simulation; however, due to the high computational burden of large-scale scenario sets, a scenario reduction strategy based on the Kantorovich Distance Matrix (KDM) is applied. This technique prunes redundant, similar scenarios while retaining a diverse and representative subset, reducing the original set to a manageable size. The KDM-based reduction process follows the following steps:

**Stage 1**: Produce the number of scenarios $\xi_{s}$(1,2,….,$N_{s}$), then estimate the distance among all scenarios $D(\xi_{s},\xi_{s^{'}})$=$\left| \xi_{s}-\xi_{s^{'}} \right|, s,s^{'}=1,2,\ldots..,N_{s}$ and set an initial probability to each scenario $\pi_{s}=\frac{1}{N_{s}}$. The initial probability for each scenario is calculated, in this step, simply by assuming that all generated scenarios are equally likely before any reduction is applied.

**Stage 2**: The algorithm identifies the two scenarios that are most similar to each other based on their distance. This can be formulated as: $D\left( s,s^{'} \right)$=$\min D\left( \xi_{s},\xi_{s^{'}} \right), s,s^{'}\in\xi_{s}$, $s\neq s^{'}.$

**Stage 3**: The algorithm chooses *s* as the one with the smaller distance calculated as $s=\pi_{s}\min D\left( \xi_{s},\xi_{s^{'}} \right)$, then remove the weaker scenario among the collection of scenarios, the updated set of scenarios becomes $S^{'}=S\backslash s$, the probability of the deleted scenario *s* is added to the closest remaining scenario $s^{'}$ as $\pi_{s}^{'}=\pi_{s}^{'}+\pi_{s}$.

**Stage 4**: Once the required number of reduced scenarios is reached, keep repeating Steps 2 and 3.

”

**PV Surrogate Model: Temperature, Clipping, and Low-Irradiance Curvature:** We acknowledge that our PV model is a simplified surrogate, chosen for computational efficiency within large-scale scenario evaluation. In our work, temperature derating is implicitly included by treating PV rated power as an effective derated capacity. Additionally, inverter clipping is enforced by the upper bound, which ensures AC-side clipping. This is illustrated through Eq. (30) in the revised manuscript: $0\leq P_{PV}\leq P_{PV}^{UL}$.

In addition, low-irradiance curvature has a negligible impact on hosting capacity because network constraints are driven by moderate–high generation hours, not hours where irradiance is < 150 W/m². To clarify these points, the following paragraph has been added to the revised manuscript in the related part:

“

PV output is modeled using a lightweight piecewise surrogate: quadratic for irradiance below $I_{c}$ W/m² and linear above, scaled by an effective rated power that embeds temperature and design derating. Clipping is enforced by the PV rated power bound. Since hosting-capacity limits occur mainly during high irradiance, the low-irradiance curvature has negligible effect on feeder-level hosting-capacity results. The framework can accommodate more detailed PV models if desired.

”

**EV Demand Modeling and Energy Consistency**: We clarified that this study adopts a feeder-level aggregate EV demand model rather than per-vehicle SOC dynamics, which is a valid modeling choice for feeder hosting-capacity studies. Specifically, arrival time, departure time, and SOC distributions are used to produce aggregate hourly EV demand trajectories for each of the 1000 initial Monte Carlo scenarios. These trajectories reflect typical dwell-time and energy requirements at the population level. The optimization does not schedule individual vehicles but instead coordinates smart-inverter DG and aggregate EV demand at each hour. To clarify these points, the following paragraph has been added to the revised manuscript:

“

*Individual EVs are not simulated; instead, aggregate hourly EV charging-demand trajectories are generated from probabilistic arrival, departure, and SOC distributions and included in the 1000 Monte Carlo samples. After scenario reduction, the optimization uses these aggregate EV profiles directly. This feeder-level modeling approach is appropriate for hosting-capacity assessment and avoids the need for per-vehicle SOC dynamics, which are not required for the scope of this study.*

”

**Correcting the Weibull Distribution Parameter Notation:** We thank the reviewer for pointing out the confusion in the Weibull notation. The equations used the standard form with $\lambda_{k}$ as the shape parameter and $\theta_{k}$ as the scale parameter, but the text incorrectly reversed these roles. In the revised manuscript, we now state explicitly that $\lambda_{k}$ is the shape parameter and $\theta_{k}$ is the scale parameter, while keeping the CDF $F\left( \omega_{k} \right)=1-exp\left( -\frac{\omega_{k}}{\theta_{k}} \right)^{\lambda_{k}}$ unchanged.

**Authors’ action:** To address the reviewer’s concerns, substantial revisions have been made to clarify the uncertainty modeling framework. Explanatory paragraphs have been added to the relevant subsections to explicitly describe the implicit temporal dependence among PV, wind, load, and EV processes; the detailed scenario generation and reduction steps from 1000 Monte Carlo samples to 10 representative, equally probable scenarios; the justification for the PV surrogate model, including effective derating, clipping bounds, and the negligible impact of low-irradiance curvature; and the use of aggregate EV demand modeling appropriate for feeder-level analysis. In addition to correcting the Weibull distribution parameter notation.

**Reviewer #3, Concern #5: Metrics are fragile and allow gaming. DG-HC and EV-HC are defined as hourly power ratios without energy, arrival, or dwell consistency for EVs. The voltage deviation index lacks clear aggregation across scenarios and time. These choices weaken any claim about system-wide hosting capacity.**

**Authors’ response:** Thank you for this important comment. We agree that metric definitions must prevent “trivial” solutions and clearly communicate performance across time and uncertainty. In the revised manuscript, we clarify the intent and interpretation of the hourly hosting indices, strengthen the EV hosting metric to ensure energy consistency, and add explicit aggregation and reporting across hours and scenarios (including duration and probability summaries).

4. EV-HC: enforcing energy consistency (avoiding gaming)

We agree that an instantaneous hourly power ratio alone can be gamed if it does not ensure that the demanded charging energy is actually delivered over the vehicle’s dwell time. To address this, we revised the EV hosting definition to be energy consistent. In particular, for each hour 𝑘 and scenario 𝑠, the delivered charging energy is defined as:

$${EV-HC}_{s}^{k}=\frac{\sum_{r=1}^{C} E_{{EV}_{s,r}}^{k}}{\sum_{r=1}^{C} E_{EV,r}^{rated}}$$

(21)

$$E_{{EV}_{s,r}}^{k}=P_{{EV}_{s,r}}^{k}\Delta t$$

(22)

5. DG-HC: power-based hosting is standard; we clarify scope and add an energy-based summary

We acknowledge the reviewer’s concern and clarify that DG hosting capacity in distribution networks is classically defined as the maximum instantaneous DG power injection that can be accommodated without violating network constraints (voltage limits, thermal limits, and losses). For this reason, DG-HC is formulated in our study as an hourly, scenario-specific, power-based hosting ratio (kW/kW). This aligns with established feeder hosting-capacity practice, where feasibility is evaluated per operating condition, and it matches the planning intent of our work, which is to assess admissible DG injection under each hour’s operational constraints. In addition, to avoid ambiguity in interpretation and to ensure a fully normalized metric, the DG-HC expression has been reformulated in the revised manuscript as the ratio between the allowable DG power injection and the aggregate rated capacity of all installed DG units. This normalization clarifies the metric, eliminates any possibility of “gaming,” and renders DG-HC fully comparable across scenarios and hours.

6. Voltage deviation index: aggregation across time/scenarios and probability/duration reporting

We thank the reviewer for highlighting the issue of aggregation and interpretability of voltage-deviation metrics. In our study, the voltage deviation index (DI) is defined at each hour $k$ and for each retained scenario $s$ as:

$${DI}_{s}^{k}=\sum_{i=1}^{N_{B}} \left| 1-V_{s,i}^{k} \right|, \forall s, \forall k$$

(24)

This formulation intentionally computes DI on an hourly and scenario-specific basis, because the 10 retained scenarios represent distinct deterministic operating conditions, each corresponding to a unique realization of EV behavior and load uncertainty after scenario reduction.

Thus, the reviewer’s concern about aggregation over time or probability is addressed as follows:

- **Scenario-specific evaluation avoids “gaming”**: By computing DI for every hour and for every scenario, the formulation prevents masking high-voltage deviations in certain hours by averaging them with other conditions. The optimization process is therefore exposed to the worst feasible deviations in any scenario–hour combination.
- **Hourly evaluation is consistent with operational constraints**: All operational constraints in the proposed formulation, including voltage limits, thermal loading, and power-loss boundaries, are enforced at every hour and for every scenario. Therefore, computing DI at each hour mirrors standard practice in feeder hosting-capacity assessment, where constraint violations cannot be averaged over time or scenarios.
- **Clarity and transparency**: The revised manuscript explicitly states that the 10 scenarios are handled as 10 deterministic operating conditions, and DI is evaluated independently for each condition. This provides a transparent framework where duration/probability concerns are handled through scenario probabilities, while voltage-feasibility concerns are handled through per-hour enforcement.

**Authors’ action:** In the revised manuscript, we clarified and strengthened the definitions and metrics to prevent potential gaming. The EV-HC metric was reformulated to enforce energy consistency by measuring delivered charging energy over the dwell period rather than relying solely on instantaneous power. The DG-HC metric was clarified as a standard hourly, power-based ratio and reformulated relative to the aggregate rated DG capacity to ensure normalization and comparability across scenarios and hours. The voltage deviation index (DI) was clarified to be calculated per hour and per scenario, with all operational constraints, including voltage limits, thermal loading, and power-loss boundaries, enforced on an hourly basis.

**Reviewer #3, Concern #6: Optimization and benchmarking are not reproducible. The narrative states weakest-agent reinitialization while the equation only shrinks positions. Constraint handling, penalties or repair rules, stopping rules, random seeds, number of runs, and statistical tests are not provided. For a rigorous comparator on stochastic robust scheduling with coordinated multi-energy interactions and a tractable single-level reformulation, see DOI: 10.1016/j.segan.2025.102024. This work’s bi-level game, TSSRO, SNCGAN scenario generation, and ADMM with column-and-constraint generation provide a transparent baseline for uncertainty treatment and solution quality reporting.**

**Authors’ response:** We thank the reviewer for highlighting this point. In the revised manuscript, we have clarified and fully aligned the SFOA description with its mathematical implementation:

1. **Regeneration step:** As shown in Eq. (19), regeneration is applied only to the worst-ranked starfish using an exponential decay operator, consistent with the original SFOA formulation, rather than random reinitialization. Boundary constraints are enforced immediately after regeneration.
2. **Constraint handling:** In the revised manuscript, we clarify that hard constraints (bus voltages, active power loss, smart inverter constraints) are managed via a penalty-based approach. Any candidate solution violating one or more constraints is penalized by setting the hosting capacities terms (DG-HC, EV-HC) to zero and computing the objective with weighted penalty terms. It is worthy noting that, in the updated objective function formulation, the hosting capacities terms (DG-HC, EV-HC) do not appear in the denominator, eliminating any risk of division by zero when penalizing infeasible solutions.
3. **Stopping criterion, random seeds, and reproducibility:** Each optimization run uses *T_max_* iterations. To validate the SFOA’s performance, 10 independent runs were performed for hour 13 of scenario 5 (cases 2, 3, and 4) and compared with three other optimizers (Section 5.3). Based on this assessment, the SFOA was executed once per hour per scenario for all other analyses. Fixed random seeds were used for reproducibility of the assessment runs. These updates ensure consistency between the narrative, equations, and implementation, addressing reproducibility concerns.

To clarify these points, the following paragraphs have been added to the revised manuscript:

“

*In accordance with the original SFOA formulation explained in [35], the regeneration mechanism is applied only to the worst-ranked starfish (i = Np) in each iteration. Instead of full random reinitialization, regeneration is performed using an exponential decay operator that gradually attenuates the position vector as iterations progress, encouraging global exploration while maintaining stability.*

”

“

*Boundary constraints are enforced immediately after regeneration using the original SFOA boundary-repair rule. This ensures consistency between the narrative description, mathematical model, and implementation, enabling reproducibility. The SFOA was executed with population size Np = 50 and maximum iterations T_max_ = 300. The exploration and exploitation phases were performed as described, with the preying sub-process refining candidate positions and regeneration applied to the worst-ranked individual. Hard constraints, including bus voltage limits, feeder ampacity, total active power loss, inverter capacity limits, and inverter characteristic voltages ordering, are enforced using a penalty-based approach. Candidate solutions violating any of these constraints are assigned zero values for the hosting capacities terms (DG-HC and EV-HC), and the objective function is penalized accordingly. To validate optimizer performance, 10 independent runs were conducted for hour 13 of scenario 5 (cases 2, 3, and 4) and compared with three other optimizers (see Section 5.3). Based on this assessment, the SFOA was run once per hour per scenario for all other analyses. The stopping criterion was T_max_ iterations, with no early termination.*

”

**Authors’ action:** We revised the manuscript to ensure full reproducibility and clarity of the SFOA optimization procedure. The regeneration step is now described as an exponential decay operator applied only to the worst-ranked starfish, with boundary handling explicitly specified. Hard constraints (bus voltages, power loss, and inverter limits) are managed via a penalty-based approach, in which infeasible solutions are assigned zero hosting-capacity terms without causing division-by-zero errors. Algorithmic parameters, stopping criteria, random seeds, and the number of runs for the comparative assessment are explicitly stated. These updates align the narrative, equations, and implementation, ensuring that the optimization procedure is fully reproducible and transparent.

**Regarding the suggested comparator (DOI: 10.1016/j.segan.2025.102024):**

We thank the reviewer for pointing out this rigorous comparator. The referenced study employs a hybrid game-theoretic scheduling framework for multi-integrated energy microgrids, using TSSRO and SNCGAN-generated scenarios to manage economic interactions, profit allocation, and multi-energy uncertainties, solved through an ADMM-based single-level reformulation. By contrast, our work targets a different problem layer, technical hosting-capacity enhancement in distribution networks, emphasizing voltage regulation, active/reactive coordination, and uncertainty-driven DG–EV accommodation rather than market games or profit-based scheduling. A paragraph summarizing and contrasting these methodological differences has been added to the revised manuscript in the relevant section.

In addition, we revised the manuscript to ensure full reproducibility and transparency of the SFOA optimization. The regeneration step is described as an exponential decay operator applied only to the worst-ranked starfish, with boundary handling explicitly specified. Hard constraints are managed via a penalty-based approach, with infeasible solutions assigned zero hosting-capacity terms without causing division-by-zero errors. Algorithmic parameters, stopping criteria, random seeds, and the number of runs for the comparative assessment are explicitly stated. Furthermore, the suggested comparator [12] was reviewed, and a paragraph contrasting its market-oriented, hybrid game-theoretic methodology with our technical hosting-capacity enhancement focus was added. These updates align the narrative, equations, implementation, and benchmarking discussion, ensuring transparency and reproducibility.

**Reviewer #3, Concern #7: Positioning relative to low-carbon dispatch frameworks is weak. The manuscript does not connect its objectives to explicit carbon mechanisms or flexibility metrics used in integrated energy systems. For a multi-objective day-ahead strategy that embeds a reward–penalty carbon trading mechanism, coordinated carbon capture, and quantitative flexibility indicators with an MILP-equivalent reformulation, see DOI: 10.3390/app122312309. Citing and contrasting against this framework will clarify how the present method addresses economy, flexibility, and emissions.**

**Author action:** We thank the reviewer for highlighting the importance of positioning our work relative to low-carbon dispatch frameworks. We agree that recent studies have developed multi-objective coordination models that explicitly incorporate carbon-emission mechanisms. In response, the literature review subsection of the Introduction has been strengthened by including and contrasting the study reported in DOI: 10.3390/app122312309 with our work. The following paragraph has been added to the revised manuscript in the relevant section:

“

*However, some recent studies have considered multi-objective coordination models that explicitly incorporate carbon-emission mechanisms. For example, the work in [23] proposes a day-ahead dispatch strategy for park-level integrated energy systems that integrates a reward–penalty carbon trading mechanism, coordinated carbon-capture operation, and flexibility indicators, reformulated into a mixed-integer linear programming (MILP) framework to balance economic cost, carbon cost, and operational flexibility. In contrast, the present study focuses on a different layer of the power system and a different objective: enhancing DG and EV hosting capacities in distribution networks under renewable and EV uncertainty. Rather than optimizing carbon economics, our framework emphasizes technical feasibility—voltage regulation, loss minimization, and coordinated smart-inverter Volt/VAR and EV charging control—thereby providing a complementary contribution aimed at increasing renewable and EV accommodation capability at the distribution-system level.*

”

**Authors’ action:** A comparative paragraph has been added to the Literature Review subsection of the Introduction. The new text summarizes the contribution of the cited low-carbon dispatch study and contrasts it with our focus on uncertainty-driven hosting-capacity enhancement and coordinated SI–EV control at the distribution-system level. The new citation (DOI: 10.3390/app122312309) has been incorporated accordingly.
