# Supplementary material for: Joint optimization of smart inverters and EV charging coordination for enhanced DG-EV hosting capacity under uncertain conditions for resilient distribution systems
Source: PLoS One. 2026 Jul 6;21(7):e0350725. doi: 10.1371/journal.pone.0350725 (PMC13336192; doi:10.1371/journal.pone.0350725)
Supplement: Attachment — Submitted filename: Reviewers comments.docx [file pone.0350725.s004.docx]

**Response to the Reviewer Comments**

**Dear Editor and Reviewers**

*The manuscript has been revised based on the precise comments of the respected reviewers. The authors hope the revised version of the manuscript fulfills all expectations of the reviewers and editor. The responses provided by the authors to your comments are as follows:*

**Reviewer #4**

**Reviewer #4, Concern #1:** **The authors should briefly explain how the SOA performs when the system becomes larger. It would also help to include a small table or note showing the average time taken for each run so readers can understand how practical the method is.**

**Authors’ response:**

The authors thank the reviewer for this valuable and practical suggestion. We agree that providing insight into the computational performance and scalability of the proposed algorithm enhances the applicability and transparency of the study.

In response, additional simulations have been conducted to evaluate the execution time of the proposed SFOA-based optimization framework for different system sizes and case studies. The execution time was recorded for Cases 2–4 under representative operating conditions for both the IEEE 33-bus system and the larger Cairo 59-bus distribution system.

The results, now included in the revised manuscript in a dedicated paragraph (subsection 5.3) and summarized in Table 7, show that execution time increases with system size, as expected. Specifically, the runtime increases from approximately 7–9 minutes for the IEEE 33-bus system to about 28–32 minutes for the Cairo 59-bus system. Despite this increase, the overall computational burden remains practical for offline planning studies. These additions provide a clearer understanding of the practical performance of the proposed method and its applicability to larger-scale systems.

Table 7: Computational time of the proposed SOA for different case studies and system sizes, Scenario 5

| System | Case | Time (s) |
| --- | --- | --- |
| IEEE 33-bus (*h*=13) | Case 2 | 427 |
| IEEE 33-bus (*h*=13) | Case 3 | 432 |
| IEEE 33-bus (*h*=13) | Case 4 | 547 |
| Cairo 59-bus (*h*=10) | Case 2 | 1710 |
| Cairo 59-bus (*h*=10) | Case 3 | 1728 |
| Cairo 59-bus (*h*=10) | Case 4 | 1895 |

**Authors’ action:** A new paragraph and table have been added to report the execution time of the proposed method for different system sizes, demonstrating its computational performance and scalability.

**Reviewer #4, Concern #2:** **It would be good to add a short explanation for reducing 1,000 scenarios to 10 using the Kantorovich Distance Matrix. The authors may also comment on whether using more scenarios like 20 or 50 changes the results much or if the results stay almost the same.**

**Authors’ response:**

The authors sincerely thank the reviewer for this insightful and constructive comment. We agree that providing a clearer explanation of the scenario reduction process and evaluating the impact of the number of representative scenarios enhances the robustness and transparency of the study.

In response, the description of the Kantorovich Distance Matrix (KDM)-based scenario reduction technique has been expanded in the revised manuscript. Additional clarification has been included to better explain how the distance between scenarios is evaluated, how redundant scenarios are iteratively eliminated, and how probability redistribution preserves the statistical characteristics of the original Monte Carlo-generated dataset. The revised text also explicitly highlights that the reduction from 1,000 scenarios to a smaller representative subset is achieved without significant loss of information, while substantially improving computational efficiency.

Second, an additional sensitivity analysis has been conducted by increasing the number of representative scenarios from 10 to 20. The optimization problem (Case 3) was re-evaluated for all scenarios, and the results were compared in terms of hourly DG installed capacity and EV charging demand.

The findings, presented in the revised manuscript (Figs. 21 and 22), demonstrate that the results obtained using 10 and 20 scenarios are highly consistent. The average hourly profiles are nearly identical, and the differences in variability are minimal. Specifically, the aggregated standard deviation over the 24-hour period shows only a marginal increase when using 20 scenarios (DG: 14.6166 to 14.6749; EV demand: 4.1558 to 4.1768), confirming that the additional scenarios provide only limited improvement in representing system uncertainty. In contrast, the computational burden increases significantly, with execution time approximately doubling when using 20 scenarios. This confirms that using 10 representative scenarios provides a sufficiently accurate representation of system uncertainty while maintaining computational efficiency.

(a)

(b)

Fig. 21. Comparative results derived from the stochastic framework across Case 3 of the IEEE 33-bus system under 20 representative scenarios: (a) hourly recorded DG installed capacity, (b) hourly recorded EV stations energy.

(a)

(b)

Fig. 22. Average value of hourly-basis results for 10 and 20 representative scenarios: (a) DG installed capacity, (b) EV stations energy.

**Authors’ action:** The explanation of the KDM-based scenario reduction method has been expanded. Additionally, a sensitivity analysis using 20 representative scenarios has been included, supported by Figs. 21 and 22 and quantitative metrics, demonstrating that increasing the number of scenarios has a negligible impact on results while significantly increasing computational cost; thus, the use of 10 scenarios is justified.

**Reviewer #4, Concern #3:** **The paper should include a short discussion about real world communication issues like delay, data loss, or communication failure between smart inverters and EV chargers. This will help readers understand how strong the system is in real conditions.**

**Authors’ response:** The authors sincerely thank the reviewer for this insightful and practical suggestion. We fully agree that considering real-world communication constraints enhances the applicability and robustness of the proposed framework. In response, a new subsection titled “5.3 Practical Communication Considerations and System Robustness” has been added to Section 5 in the revised manuscript. This subsection discusses the potential impacts of communication delays, data loss, and temporary communication failures on coordination between smart inverters and the EV charging infrastructure. Specifically, the discussion highlights that the proposed stochastic optimization framework operates on an hourly timescale and relies on probabilistic, scenario-based modeling, thereby inherently reducing sensitivity to short-term communication delays. Additionally, the roles of data-handling strategies (e.g., buffering and interpolation) and local autonomous control mechanisms (such as Volt/VAR control) are emphasized as key factors that ensure system reliability under imperfect communication conditions. Finally, the manuscript now acknowledges that integrating detailed communication network modeling and cyber-physical co-simulation constitutes an important avenue for future work.

**Authors’ action:** A new subsection titled “5.3 Practical Communication Considerations and System Robustness” has been added to the revised manuscript. This subsection briefly discusses the impact of communication delays, data loss, and potential communication failures between smart inverters and EV charging infrastructure. It also clarifies the inherent robustness of the proposed stochastic framework, owing to its scenario-based nature, and highlights the role of local control strategies in maintaining reliable operation under imperfect communication conditions.

**Reviewer #4, Concern #4:** **For better understanding of the multi objective results, the authors may mark a best compromise solution on the Pareto graph. A simple method like TOPSIS or fuzzy logic can be used so readers can easily see the best balanced point.**

**Authors’ response:**

The authors sincerely thank the reviewer for this valuable suggestion. We fully agree that identifying the best-compromise solution significantly enhances the clarity and practical interpretation of multi-objective optimization results.

In response, a new subsection titled “5.5. Identification of the Best Compromise Solution Using TOPSIS” has been added to the revised manuscript. In this subsection, the Technique for Order Preference by Similarity to Ideal Solution (TOPSIS) is employed as a multi-criteria decision-making tool to systematically rank all weighting-coefficient combinations.

The analysis considers multiple performance criteria, including DG installed capacity and EV charging demand as benefit criteria, and voltage deviation, power losses, and constraint violations as cost criteria. All criteria are normalized and equally weighted to ensure an unbiased comparison. A penalty term is incorporated to account for infeasible solutions, ensuring they are appropriately penalized without being excluded.

The results demonstrate that the proposed weighting combination achieves the highest closeness coefficient (≈ 0.69), identifying it as the most balanced solution among all alternatives. To further improve visualization and reader understanding, a new figure (Fig. 20) has been added to the revised manuscript, illustrating the TOPSIS-based ranking and clearly highlighting the selected best-compromise solution.

Fig. 20. TOPSIS ranking of weighting-coefficient combinations, highlighting the proposed solution as the best compromise.

**Authors’ action:** A new subsection (Subsection 5.5) and an additional figure (Fig. 20) have been included in the revised manuscript to present a TOPSIS-based analysis, identifying and highlighting the best compromise solution among the considered weighting combinations.

**Reviewer #4, Concern #5:** **The conclusion can include one or two lines about future work. The authors may mention that the method can also be used for other grid services like frequency control or black start support. This will make the paper more complete.**

**Authors’ response:**

The authors thank the reviewer for this valuable suggestion.

The conclusion Section has been amended to include a brief discussion on potential future extensions of the developed stochastic optimization framework toward additional grid services. Specifically, it is now indicated that coordinated control of DG and EV charging systems can be adapted to support services such as frequency control through active power management and system restoration during black-start conditions. The revised text also notes that such applications would require incorporating dynamic modeling and multi-timescale control strategies.

**Authors’ action:** Future work paragraph has been added to the conclusion, highlighting the potential extension of the proposed framework to support grid services such as frequency control and black start operations.

*-------------------------------------------------------------------------------------------------------------------*

*Finally, the authors hope that the revised version of the manuscript meets all expectations of the reviewers and the editor.*
