# Supplementary material for: Joint optimization of smart inverters and EV charging coordination for enhanced DG-EV hosting capacity under uncertain conditions for resilient distribution systems
Source: PLoS One. 2026 Jul 6;21(7):e0350725. doi: 10.1371/journal.pone.0350725 (PMC13336192; doi:10.1371/journal.pone.0350725)
Supplement: Peer review history

# Peer review: Decision Letter 0

**DOI:** 10.1371/journal.pone.0350725.r001

**Authors:** Qinglin Meng

28 Nov 2025

PONE-D-25-59694

Joint Optimization of Smart Inverters and EV Charging Coordination for Enhanced DG-EV Hosting Capacity Under Uncertain Conditions for Resilient Distribution Systems

PLOS ONE

Dear Dr. Ali,

Thank you for submitting your manuscript to PLOS ONE. After careful consideration, we feel that it has merit but does not fully meet PLOS ONE’s publication criteria as it currently stands. Therefore, we invite you to submit a revised version of the manuscript that addresses the points raised during the review process.

Please submit your revised manuscript by Jan 12 2026 11:59PM. If you will need more time than this to complete your revisions, please reply to this message or contact the journal office at plosone@plos.org. When you're ready to submit your revision, log on to https://www.editorialmanager.com/pone/ and select the 'Submissions Needing Revision' folder to locate your manuscript file.

Please include the following items when submitting your revised manuscript:

- A rebuttal letter that responds to each point raised by the academic editor and reviewer(s). You should upload this letter as a separate file labeled 'Response to Reviewers'.
- A marked-up copy of your manuscript that highlights changes made to the original version. You should upload this as a separate file labeled 'Revised Manuscript with Track Changes'.
- An unmarked version of your revised paper without tracked changes. You should upload this as a separate file labeled 'Manuscript'.

If you would like to make changes to your financial disclosure, please include your updated statement in your cover letter. Guidelines for resubmitting your figure files are available below the reviewer comments at the end of this letter.

If applicable, we recommend that you deposit your laboratory protocols in protocols.io to enhance the reproducibility of your results. Protocols.io assigns your protocol its own identifier (DOI) so that it can be cited independently in the future. For instructions see: https://journals.plos.org/plosone/s/submission-guidelines#loc-laboratory-protocols. Additionally, PLOS ONE offers an option for publishing peer-reviewed Lab Protocol articles, which describe protocols hosted on protocols.io. Read more information on sharing protocols at https://plos.org/protocols?utm_medium=editorial-email&utm_source=authorletters&utm_campaign=protocols.

We look forward to receiving your revised manuscript.

Kind regards,

Qinglin Meng, Ph.D.

Academic Editor

PLOS ONE

Journal Requirements:

When submitting your revision, we need you to address these additional requirements.

1. Please ensure that your manuscript meets PLOS ONE's style requirements, including those for file naming. The PLOS ONE style templates can be found at

https://journals.plos.org/plosone/s/file?id=wjVg/PLOSOne_formatting_sample_main_body.pdf and https://journals.plos.org/plosone/s/file?id=ba62/PLOSOne_formatting_sample_title_authors_affiliations.pdf

2. Please note that PLOS One has specific guidelines on code sharing for submissions in which author-generated code underpins the findings in the manuscript. In these cases, we expect all author-generated code to be made available without restrictions upon publication of the work. Please review our guidelines at https://journals.plos.org/plosone/s/materials-and-software-sharing#loc-sharing-code and ensure that your code is shared in a way that follows best practice and facilitates reproducibility and reuse.

3. Thank you for stating the following financial disclosure:

[The authors extend their appreciation to Prince Sattam bin Abdulaziz University for funding this research work through the project number PSAU/2024/01/31839.].

Please state what role the funders took in the study.  If the funders had no role, please state: "The funders had no role in study design, data collection and analysis, decision to publish, or preparation of the manuscript."

If this statement is not correct you must amend it as needed.

Please include this amended Role of Funder statement in your cover letter; we will change the online submission form on your behalf.

4. Thank you for stating the following in the Acknowledgments Section of your manuscript:

[The authors extend their appreciation to Prince Sattam bin Abdulaziz University for funding this research work through the project number PSAU/2024/01/31839.]

We note that you have provided funding information that is currently declared in your Funding Statement. However, funding information should not appear in the Acknowledgments section or other areas of your manuscript. We will only publish funding information present in the Funding Statement section of the online submission form.

Please remove any funding-related text from the manuscript and let us know how you would like to update your Funding Statement. Currently, your Funding Statement reads as follows:

[The authors extend their appreciation to Prince Sattam bin Abdulaziz University for funding this research work through the project number PSAU/2024/01/31839.]

Please include your amended statements within your cover letter; we will change the online submission form on your behalf.]

5. We note that Figures 3 and 7 in your submission may contain copyrighted images. All PLOS content is published under the Creative Commons Attribution License (CC BY 4.0), which means that the manuscript, images, and Supporting Information files will be freely available online, and any third party is permitted to access, download, copy, distribute, and use these materials in any way, even commercially, with proper attribution. For more information, see our copyright guidelines: http://journals.plos.org/plosone/s/licenses-and-copyright.

We require you to either (1) present written permission from the copyright holder to publish these figures specifically under the CC BY 4.0 license, or (2) remove the figures from your submission:

1. You may seek permission from the original copyright holder of Figures 3 and 7 to publish the content specifically under the CC BY 4.0 license.

We recommend that you contact the original copyright holder with the Content Permission Form (http://journals.plos.org/plosone/s/file?id=7c09/content-permission-form.pdf) and the following text:

“I request permission for the open-access journal PLOS ONE to publish XXX under the Creative Commons Attribution License (CCAL) CC BY 4.0 (http://creativecommons.org/licenses/by/4.0/). Please be aware that this license allows unrestricted use and distribution, even commercially, by third parties. Please reply and provide explicit written permission to publish XXX under a CC BY license and complete the attached form.”

Please upload the completed Content Permission Form or other proof of granted permissions as an "Other" file with your submission.

In the figure caption of the copyrighted figure, please include the following text: “Reprinted from [ref] under a CC BY license, with permission from [name of publisher], original copyright [original copyright year].”

2. If you are unable to obtain permission from the original copyright holder to publish these figures under the CC BY 4.0 license or if the copyright holder’s requirements are incompatible with the CC BY 4.0 license, please either i) remove the figure or ii) supply a replacement figure that complies with the CC BY 4.0 license. Please check copyright information on all replacement figures and update the figure caption with source information. If applicable, please specify in the figure caption text when a figure is similar but not identical to the original image and is therefore for illustrative purposes only.

If the reviewer comments include a recommendation to cite specific previously published works, please review and evaluate these publications to determine whether they are relevant and should be cited. There is no requirement to cite these works unless the editor has indicated otherwise.

Additional Editor Comments:

Please rewrite the abstract/introduction, remove confusing “biology-style” images, spell out exactly how the objective aggregates hours and scenarios, add the missing inverter/network constraints, clarify uncertainty and correlations, and provide code/data plus comparisons to strong baselines with run-to-run statistics. Follow the reviewers comments.

[Note: HTML markup is below. Please do not edit.]

Reviewers' comments:

Reviewer's Responses to Questions

**Comments to the Author**

1. Is the manuscript technically sound, and do the data support the conclusions?

The manuscript must describe a technically sound piece of scientific research with data that supports the conclusions. Experiments must have been conducted rigorously, with appropriate controls, replication, and sample sizes. The conclusions must be drawn appropriately based on the data presented.

Reviewer #1: No

Reviewer #2: Yes

Reviewer #3: No

**********

2. Has the statistical analysis been performed appropriately and rigorously?

Reviewer #1: No

Reviewer #2: Yes

Reviewer #3: No

**********

3. Have the authors made all data underlying the findings in their manuscript fully available?

The PLOS Data policy requires authors to make all data underlying the findings described in their manuscript fully available without restriction, with rare exception (please refer to the Data Availability Statement in the manuscript PDF file). The data should be provided as part of the manuscript or its supporting information, or deposited to a public repository. For example, in addition to summary statistics, the data points behind means, medians and variance measures should be available. If there are restrictions on publicly sharing data—e.g. participant privacy or use of data from a third party—those must be specified.

Reviewer #1: No

Reviewer #2: Yes

Reviewer #3: No

**********

4. Is the manuscript presented in an intelligible fashion and written in standard English?

PLOS ONE does not copyedit accepted manuscripts, so the language in submitted articles must be clear, correct, and unambiguous. Any typographical or grammatical errors should be corrected at revision, so please note any specific errors here.

Reviewer #1: No

Reviewer #2: Yes

Reviewer #3: No

**********

5. Review Comments to the Author

Please use the space provided to explain your answers to the questions above. You may also include additional comments for the author, including concerns about dual publication, research ethics, or publication ethics. (Please upload your review as an attachment if it exceeds 20,000 characters)

Reviewer #1: This paper studies scenario-based coordination of EV charging and smart-inverter Volt/VAR control to increase distribution hosting capacity, solved with a starfish-inspired metaheuristic. The topic is relevant, but the manuscript contains structural, modeling, and rigor issues that block acceptance at it’s current form.

1. The abstract does not follow a research-article arc (problem → proposed method → quantitative outcomes) and is verbose; the Introduction is short, reads like a report, and relies on long bullet lists that dilute technical focus.

2. Prior work coverage is shallow and uneven: recent (2023–2025) state-of-the-art on multi-objective hosting capacity, coordinated SI+EV scheduling, chance-/robust-constrained formulations, and standard Pareto baselines is underrepresented; self-citation and regional clustering are noticeable.

3. The multi-objective formulation is ill-posed: use of reciprocals of hosting-capacity indices is numerically unstable, and the optimization block lacks an explicit aggregation over time and scenarios, creating ambiguity about what is being minimized.

4. Electrical feasibility is incompletely modeled: Volt/VAR set-points are not coupled to inverter apparent-power limits, rate/step constraints, or guaranteed 4-point curve order; feeder ampacity/protection assumptions are insufficiently specified.

5. Uncertainty modeling is inconsistent: dependence among PV, wind, load, and EV processes is unspecified; scenario generation/reduction settings and retained probabilities are not documented; the PV surrogate ignores temperature and inverter clipping and exhibits unrealistic curvature at low irradiance.

6. Results introduce bias: solutions are filtered post hoc to keep only cases with losses below the base case, obscuring genuine trade-offs and affecting comparability.

7. Optimization description is not reproducible: discrepancies exist between the narrative and equations for the “regeneration” step; constraint handling (penalties/repair/feasibility rules), stopping criteria, random seeds, number of runs, and statistical testing across runs are not reported.

8. Definitions and metrics for DG-HC and EV-HC permit gaming: instantaneous power-ratio indices (per hour) are used without arrival/dwell/energy consistency for EVs; voltage-deviation indices lack clear aggregation across scenarios and time and do not convey duration/probability.

9. Figures and tables are inconsistent: multiple plots appear as low-resolution screenshots with mismatched fonts and missing units; captions lack scenario/hour context; parameter tables mix solver knobs with physical limits and omit key network/penalty parameters needed for replication.

10. Unnecessary and misleading graphics (Star fish image) are included: several image choices and stylistic elements evoke biological themes and distract from an energy-systems contribution, creating confusion at first glance about the paper’s domain. They must be removed.

11. Conclusions are qualitative and restate trends without precise numerical deltas, sensitivity ranges, or explicit limitations, weakening the evidential value of the study’s claims.

Reviewer #2: This paper proposes a stochastic optimization framework combining smart inverter Volt/VAR control and coordinated electric vehicle charging, utilizing Monte Carlo simulation to handle uncertainties and applying the Starfish Optimization Algorithm SFOA for solution. Although the work demonstrates sufficient simulation validation, there is still room for further improvement regarding the scientific basis of parameter selection, the theoretical depth of algorithmic comparison, and the discussion on engineering implementation.

First, regarding the setting of weighting coefficients in the multi-objective optimization, it is suggested that the authors include a necessary sensitivity analysis. Please specifically illustrate the potential impact on the optimization results when the weighting priorities change, thereby demonstrating the robustness of the current parameter selection and avoiding the limitations of relying solely on empirical selection.

Second, in terms of algorithm comparison, although the manuscript demonstrates the advantage of SFOA in convergence accuracy, the dimensions of comparison appear somewhat limited. It is recommended to supplement the study with a theoretical analysis of computational complexity using Big-O notation to more comprehensively reflect the differences in algorithmic efficiency.

Finally, the current model construction relies on the ideal assumption that the Distribution System Operator DSO can perfectly coordinate all units. To enhance the practical engineering value of the paper, it is suggested to appropriately supplement the discussion section with an analysis of challenges in actual deployment, such as the choice of communication architecture or the potential impact of signal delays on control effectiveness.

Reviewer #3: This manuscript coordinates EV charging and smart-inverter Volt/VAR to raise hosting capacity using a starfish metaheuristic. The topic is relevant, but the formulation, uncertainty treatment, benchmarking, and presentation do not yet support reliable conclusions.

1. The abstract and introduction are not in research-article form. The abstract should open with the core challenge, state the proposed coordination method and decision variables, and report key quantitative outcomes. The introduction reads like a report and lacks a tight three-item contribution statement and a critical comparison to recent scenario-based multi-energy scheduling.

2. The optimization is ill posed. The objective uses reciprocals of hosting-capacity indices, the terms are not scaled to a common magnitude, and there is no explicit aggregation over hours and scenarios. A post hoc loss filter further biases the target being minimized.

3. Electrical feasibility is incomplete. Volt/VAR points are not tied to inverter apparent-power capability, step or rate limits are not stated, and the four-point curve lacks ordering and monotonicity checks. Feeder ampacity and protection assumptions are not fully enumerated.

4. Uncertainty modeling is inconsistent. The PV surrogate ignores temperature and clipping and is nonstandard at low irradiance. Dependence among PV, wind, load, and EV is not justified. Scenario generation and reduction settings and retained probabilities are not reported. The Weibull CDF is correct but the text confuses the scale and shape parameter names.

5. Metrics are fragile and allow gaming. DG-HC and EV-HC are defined as hourly power ratios without energy, arrival, or dwell consistency for EVs. The voltage deviation index lacks a clear aggregation across scenarios and time. These choices weaken any claim about system-wide hosting capacity.

6. Optimization and benchmarking are not reproducible. The narrative states weakest-agent reinitialization while the equation only shrinks positions. Constraint handling, penalties or repair rules, stopping rules, random seeds, number of runs, and statistical tests are not provided. For a rigorous comparator on stochastic robust scheduling with coordinated multi-energy interactions and a tractable single-level reformulation, see DOI: 10.1016/j.segan.2025.102024. This work’s bi-level game, TSSRO, SNCGAN scenario generation, and ADMM with column-and-constraint generation provide a transparent baseline for uncertainty treatment and solution quality reporting.

7. Positioning relative to low-carbon dispatch frameworks is weak. The manuscript does not connect its objectives to explicit carbon mechanisms or flexibility metrics used in integrated energy systems. For a multi-objective day-ahead strategy that embeds a reward–penalty carbon trading mechanism, coordinated carbon capture, and quantitative flexibility indicators with an MILP-equivalent reformulation, see DOI: 10.3390/app122312309. Citing and contrasting against this framework will clarify how the present method addresses economy, flexibility, and emissions.

8. Figures, tables, and conclusions reduce clarity. Several plots appear to be screenshots with inconsistent fonts and missing units. Captions lack scenario and hour context. Parameter tables mix solver knobs with physical limits and omit key network and penalty parameters. Unnecessary graphics create a biology-like visual impression and mislead readers. Conclusions restate trends without aggregated deltas over all scenarios and hours, without sensitivity ranges, and without uncertainty bands.

**********

6. PLOS authors have the option to publish the peer review history of their article (what does this mean?). If published, this will include your full peer review and any attached files.

If you choose “no”, your identity will remain anonymous but your review may still be made public.

**Do you want your identity to be public for this peer review?**  For information about this choice, including consent withdrawal, please see our Privacy Policy.

Reviewer #1: No

Reviewer #2: No

Reviewer #3: No

**********

[NOTE: If reviewer comments were submitted as an attachment file, they will be attached to this email and accessible via the submission site. Please log into your account, locate the manuscript record, and check for the action link "View Attachments". If this link does not appear, there are no attachment files.]

To ensure your figures meet our technical requirements, please review our figure guidelines: https://journals.plos.org/plosone/s/figures

You may also use PLOS’s free figure tool, NAAS, to help you prepare publication quality figures: https://journals.plos.org/plosone/s/figures#loc-tools-for-figure-preparation.

NAAS will assess whether your figures meet our technical requirements by comparing each figure against our figure specifications.

# Author comment: Author response to Decision Letter 1

**DOI:** 10.1371/journal.pone.0350725.r002

10 Feb 2026

Response to the Reviewer Comments

Dear Editor and Reviewers

The manuscript has been revised based on the precise comments of the respected editor and reviewer. The authors hope the revised version of the manuscript fulfills all expectations of the reviewer and editor. The responses provided by the authors to your comments are as follows:

Editor comment

We note that Figures 3 and 7 in your submission may contain copyrighted images.

Authors’ response: We thank the Editor for pointing out the potential copyright issues in Figures 3 and 7. To fully comply with PLOS ONE’s copyright and licensing requirements, we have removed both Figure 3 and Figure 7 from the revised manuscript. As these figures were not essential to the technical content or results, their removal does not affect the methodology, analyses, or conclusions of the study.

Authors’ action: Figures 3 and 7 have been removed from the main manuscript as well as from the supplementary files. In addition, all remaining figures in the revised manuscript are original, high-resolution, and fully compliant with PLOS ONE’s licensing policy.

Reviewer #1

Reviewer #1, Concern #1: The abstract does not follow a research-article arc (problem → proposed method → quantitative outcomes) and is verbose; the Introduction is short, reads like a report, and relies on long bullet lists that dilute technical focus.

Authors’ response: We thank the reviewer for the feedback regarding the abstract and the Introduction. The abstract has been revised to follow a clear research-article arc, emphasizing the problem, the proposed method, and key quantitative outcomes in a concise manner. The updated abstract is as follows:

The rapid growth of renewable-based distributed generation (DG) and electric vehicles (EVs) poses significant operational challenges for distribution systems (DSs), particularly under uncertainties in renewable output, load demand, and EV charging behavior. Distribution system operators must therefore evaluate and enhance both DG hosting capacity (DG-HC) and EV hosting capacity (EV-HC) while maintaining voltage security and reducing losses. This study presents a stochastic, multi-objective optimization framework that jointly coordinates smart inverter (SI)-based Volt/VAR control and EV charging scheduling to simultaneously maximize DG-HC and EV-HC and minimize active power losses and voltage deviation. The framework integrates active power management through EV charging coordination and reactive power support via optimally deployed SIs. The resulting multi-objective problem is solved using the Starfish Optimization Algorithm (SFOA) and benchmarked against three established metaheuristics. The methodology is validated on the IEEE 33-bus system and a real 59-bus distribution network in Cairo, Egypt. Results show that coordinated SI–EV control increases DG-HC and EV-HC by up to 74% and 89%, respectively, and achieves voltage deviation reductions of 55% in the IEEE 33-bus system and 11% in the Cairo DS. Comparative analysis confirms that SFOA provides superior convergence and solution quality relative to the competing techniques.

In addition, the Introduction section has been substantially expanded and now includes 27 updated references. Its writing style has been enhanced and revised to improve technical clarity and readability. These changes provide a stronger and more focused context for the study and ensure up-to-date coverage of multi-objective HC assessment, coordinated SI–EV scheduling, and uncertainty-aware optimization.

Authors’ action: The abstract has been fully revised to follow a clear research-article structure, emphasizing the core problem, the proposed coordination framework, and key quantitative outcomes. The Introduction has been substantially expanded and rewritten to improve technical clarity, remove verbose bullet lists, and provide a focused discussion of the relevant literature. A total of 27 updated references have been included to ensure comprehensive coverage of multi-objective hosting capacity assessment, coordinated SI–EV scheduling, and uncertainty-aware optimization, thereby strengthening the context and contribution of the study.

Reviewer #1, Concern #2: Prior work coverage is shallow and uneven: recent (2023–2025) state-of-the-art on multi-objective hosting capacity, coordinated SI+EV scheduling, chance-/robust-constrained formulations, and standard Pareto baselines is underrepresented; self-citation and regional clustering are noticeable.

Authors’ response: We sincerely appreciate the reviewer’s observation regarding the depth and balance of the prior work coverage. In response, we have undertaken a substantial revision of the literature review to ensure a comprehensive, up-to-date, and methodologically diverse discussion of recent advancements. Specifically, we expanded the literature subsection by incorporating eight additional peer-reviewed studies published between 2023 and 2025, covering multi-objective hosting capacity assessment, coordinated smart-inverter and EV scheduling, chance- and robust-constrained formulations, and recent multi-objective optimization baselines. The revised Related Work section now synthesizes and contrasts 22 recent and diverse references, providing a significantly stronger contextual foundation for the novelty of our proposed framework.

Also, to enhance transparency and provide clearer differentiation from existing literature, we have substantially expanded Table 1, “Overview of the key contributions of this study in comparison with selected works from the literature.” The revised table now contrasts contemporary studies across multiple technical dimensions—including uncertainty modeling, SI-based reactive power support, EV charging coordination, and DG/EV hosting capacity objectives, thus presenting a more structured and rigorous positioning of our contributions.

Regarding self-citation, four of our prior works appeared in the original manuscript (numbered as [6], [8], [21], and [29] in the original manuscript; these are renumbered as [6], [8], [24], and [34] in the revised manuscript). To avoid unnecessary self-citation, references [6] and [34] in the revised manuscript have been replaced with more recent and relevant studies. However, references [8] and [24] are directly pertinent to the technical foundations of this work and could not be removed without compromising the continuity of the research context. In the revised manuscript, we explicitly contrast the current study with these two prior works and clearly highlight how the present framework addresses the research gaps that remained open in those earlier contributions.

Table 1. Overview of the key contributions of this study in comparison with selected works from the literature.

Assessment Parameters [10], [16]

[11],[15]

[14]

[18]

[19]–[21]

[23]

[22]

[25]

[4]

[13]

[26]

[8]

This work

HC evaluation Y Y Y N Y Y Y N Y Y Y Y Y

System uncertainties Y Y Y N N Y N Y N N N N Y

DG technology variety N N N N N N N Y N N Y Y Y

Active power support Y Y Y N Y Y Y Y Y Y Y Y Y

Reactive power support N Y N Y N Y Y N N Y N Y Y

EVs charging scheduling Y Y Y N N N N Y Y Y Y Y Y

Optimization objectives DG-HC maximization Y Y Y N Y Y Y N Y Y Y Y Y

EV-HC maximization N N N N N N N Y Y N Y Y Y

Loss reduction Y N N N N N N N N Y Y Y Y

Voltage profile enhancement N N N Y N Y N N N N N N Y

Authors’ action: The literature review has been substantially strengthened by adding eight recent (2023–2025) studies and expanding the discussion to a total of 22 contrasted references, ensuring balanced and up-to-date coverage of multi-objective hosting capacity assessment, coordinated SI–EV scheduling, and uncertainty-aware optimization. In addition, Table 1 has been expanded to include additional comparison dimensions and a broader set of contemporary studies. To address self-citation concerns, two earlier self-cited references were replaced with more relevant recent works, while the two technically indispensable ones were retained and explicitly contrasted with the present framework to clarify the advancements achieved.

Reviewer #1, Concern #3: The multi-objective formulation is ill-posed: use of reciprocals of hosting-capacity indices is numerically unstable, and the optimization block lacks an explicit aggregation over time and scenarios, creating ambiguity about what is being minimized.

Authors’ response: We thank the reviewer for this insightful comment. In the revised manuscript, the multi-objective optimization framework has been substantially reformulated to ensure numerical stability, proper scaling, and full clarity regarding the role of hours and scenarios.

Removal of all reciprocal terms

We fully agree that the use of 1/HC in the original objective-function formulation could lead to numerical instability when hosting-capacity values become small. In the revised work, all reciprocal terms have been removed. Additionally, hosting-capacity indices are now used in the form (1ⓜ-HC), which is bounded, smooth, and numerically stable on [0ⓜ,1]. This eliminates the ill-posedness flagged initially by the reviewer. The modified objective function becomes:

minof=min{ w_1 (1-〖DG-HC〗_s^k )+w_2 (1-〖EV-HC〗_s^k )++w_3 〖(DI〗_s^k/〖DI〗_BC)+w_4 〖(PLI〗_s^k)}, ∀ s,∀ k

(25)

Revised and normalized definitions of DG-HC and EV-HC

〖DG-HC〗_s^k=(∑_(j=1)^N▒〖P_(〖WT〗_(s,j) )〗^k + ∑_(j=1)^M▒〖P_(〖PV〗_(s,j) )〗^k )/(∑_(j=1)^N▒P_(WT,j)^rated + ∑_(j=1)^M▒P_(PV,j)^rated ) (20)

〖EV-HC〗_s^k=(∑_(r=1)^C▒E_(〖EV〗_(s,r))^k )/(∑_(r=1)^C▒E_(EV,r)^rated ) (21)

This formulation ensures that both indices lie strictly in [0,1]. Both are normalized using physically meaningful upper bounds (installed DG capacity and EV charger ratings). No variable can be artificially inflated (“gamed”), eliminating the technical weakness noted by the reviewer.

Explicit clarification of scenario handling and time resolution

We revised the manuscript to clearly explain that the optimization is not a single stochastic expected-value problem, instead, each reduced scenario s and each hour k represent a different operating condition under uncertainty. Thus, a separate deterministic optimization problem is solved independently for each pair (k,s). Therefore, no averaging or aggregation across hours or scenarios is missing; the formulation is intentionally scenario-wise and hour-wise to obtain detailed hourly hosting-capacity profiles.

Re-running all simulations with the corrected objective function

Because the corrected objective function materially changes the optimization landscape, all experiments and results have been recomputed using the revised, well-posed formulation. The updated numerical results now reflect the corrected hosting-capacity definitions and the stable objective structure.

Therefore, the optimization framework in the revised manuscript is now mathematically well posed, free of reciprocal and unbounded terms, fully normalized, explicitly scenario-wise and hour-wise, and consistent with hosting-capacity practices in the literature.

Authors’ action: In response to the reviewer’s observation, we have completely reformulated the multi-objective function to ensure mathematical well-posedness and eliminate ambiguity. Precisely, (i) all reciprocal hosting-capacity terms were removed and replaced with bounded, normalized expressions using 1-HC, eliminating numerical instability; (ii) DG-HC and EV-HC were redefined as normalized, capacity-based indices bounded in [0,1]; and (iii) scenario and hour treatment was explicitly clarified, showing that each scenario–hour pair is optimized independently rather than aggregated, thereby removing ambiguity regarding minimization across time and scenarios. The multi-objective function formulation subsection has been entirely rewritten, and all results were regenerated using the corrected, well-posed formulation.

Reviewer #1, Concern #4: Electrical feasibility is incompletely modeled: Volt/VAR set-points are not coupled to inverter apparent-power limits, rate/step constraints, or guaranteed 4-point curve order; feeder ampacity/protection assumptions are insufficiently specified.

Authors’ response: We thank the reviewer for highlighting the need for a more rigorous and electrically consistent formulation of the smart-inverter Volt/VAR model.

In the revised manuscript, the Volt/VAR characteristic is now fully specified with explicit mathematical expressions for the four-point curve, including ordered breakpoints, negative slopes, and a guaranteed monotonic, piecewise-affine profile. The intermediate points V_2j and V_3j are computed directly from the decision-variable slopes m_1j and m_2j, ensuring consistent deadband behavior. Although the original code fully enforced Volt/VAR monotonicity, breakpoint ordering, and inverter apparent-power capability limits, these implementation details were not sufficiently highlighted in the previous manuscript.

The revised version now explains these mechanisms explicitly and provides the complete mathematical formulation to reflect the model already used in the simulations. These explanations have been added to the revised manuscript in the Smart Inverter Modeling subsection:

To ensure electrical feasibility, this work adopts an explicit, capability-consistent formulation of the four-segment Volt/VAR characteristic, with constraints guaranteeing monotonicity, correct ordering of curve points, and compliance with inverter apparent-power limits. Each inverter j uses a fixed low-voltage saturation point V_1=0.91" pu" and the high-voltage saturation point V_4=1.04"  pu" . Two decision variables define the slopes in the lower and upper regions:

m_1≤0 ,m_2≤0

(32)

The intermediate breakpoints V_2j and V_3j are not free variables; they are deterministically computed from the slopes:

V_(2_j )=(-1)/m_(1_j ) +V_1 (33)

V_(3_j )=1/m_(2_j ) +V_4 (34)

These expressions guarantee that the deadband width adjusts consistently with the selected slopes. Ordering and monotonicity are enforced through the explicit constraints implemented via penalty terms in the optimization to prevent infeasible curve shapes.

V_1<V_(2_j )<V_(3_j )<V_4 (35)

The per-unit reactive power as a function of the predefined voltage at the PCC q_j (v) is defined through a continuous piecewise-affine Volt/VAR characteristic:

q_j (v)={█(1 v≤V_1@m_(1_j ) (v-V_1 )+1 V_1<v<V_(2_j ) @0 〖 V〗_(2_j )<v<V_(3_j )@m_(2_j ) (v-V_4 )-1 V_(3_j )<v< V_4 @-1 v≥V_4 )┤

(36)

The actual value of reactive-power output is then:

〖Q_j〗^inv (v)=q_j (v) Q_j^rated (37)

To ensure electrical feasibility, inverter reactive-power operation is coupled to the available apparent-power capacity:

〖(P_j^DG)〗^2+〖(〖Q_j〗^inv)〗^2≤〖(S_(rated,j)^DG)〗^2 (38)

These updates provide a fully compliant Volt/VAR model that satisfies all capability, ordering, monotonicity, and protection-related requirements. In addition, the feeder ampacity constraint has been further explained and highlighted in the revised manuscript, as shown in Eq. (27):

|I_(s,L)^K |<I_UL, ∀ L, ∀ s, ∀ k (27)

This explanation has been highlighted:

Eq. (27) constrains feeder currents to stay within the thermal ampacity, I_UL, of the conductors at every hour

Authors’ action: The smart-inverter modeling section has been strengthened to clearly present the complete detailed four-segment Volt/VAR formulation, including slope-based computation of internal breakpoints, enforced ordering and monotonicity, and coupling of reactive-power output to inverter apparent-power limits. Although all these feasibility conditions were already implemented in the original optimization code, they were not sufficiently detailed in the manuscript. The revised version now explicitly describes these mechanisms and includes complete mathematical expressions and constraints to ensure clarity and transparency.

Reviewer #1, Concern #5: Uncertainty modeling is inconsistent: dependence among PV, wind, load, and EV processes is unspecified; scenario generation/reduction settings and retained probabilities are not documented; the PV surrogate ignores temperature and inverter clipping and exh

Attachment Submitted filename: reviewers comments report V4-Shady.docx

# Peer review: Decision Letter 1

**DOI:** 10.1371/journal.pone.0350725.r003

**Authors:** Zhengmao Li

14 Apr 2026

<div>PONE-D-25-59694R1

Joint Optimization of Smart Inverters and EV Charging Coordination for Enhanced DG-EV Hosting Capacity Under Uncertain Conditions for Resilient Distribution Systems

PLOS One

Dear Dr. Ali,

Thank you for submitting your manuscript to PLOS ONE. After careful consideration, we feel that it has merit but does not fully meet PLOS ONE’s publication criteria as it currently stands. Therefore, we invite you to submit a revised version of the manuscript that addresses the points raised during the review process.

==============================

**ACADEMIC EDITOR: Please revise accordingly**

==============================

Please submit your revised manuscript by May 29 2026 11:59PM. If you will need more time than this to complete your revisions, please reply to this message or contact the journal office at plosone@plos.org. When you're ready to submit your revision, log on to https://www.editorialmanager.com/pone/ and select the 'Submissions Needing Revision' folder to locate your manuscript file.

Please include the following items when submitting your revised manuscript:

- A letter that responds to each point raised by the academic editor and reviewer(s). You should upload this letter as a separate file labeled 'Response to Reviewers'.
- A marked-up copy of your manuscript that highlights changes made to the original version. You should upload this as a separate file labeled 'Revised Manuscript with Track Changes'.
- An unmarked version of your revised paper without tracked changes. You should upload this as a separate file labeled 'Manuscript'.

If you would like to make changes to your financial disclosure, please include your updated statement in your cover letter. Guidelines for resubmitting your figure files are available below the reviewer comments at the end of this letter.

If applicable, we recommend that you deposit your laboratory protocols in protocols.io to enhance the reproducibility of your results. Protocols.io assigns your protocol its own identifier (DOI) so that it can be cited independently in the future. For instructions see: https://journals.plos.org/plosone/s/submission-guidelines#loc-laboratory-protocols. Additionally, PLOS ONE offers an option for publishing peer-reviewed Lab Protocol articles, which describe protocols hosted on protocols.io. Read more information on sharing protocols at https://plos.org/protocols?utm_medium=editorial-email&utm_source=authorletters&utm_campaign=protocols.

As the corresponding author, your ORCID iD is verified in the submission system and will appear in the published article. PLOS supports the use of ORCID, and we encourage all coauthors to register for an ORCID iD and use it as well. Please encourage your coauthors to verify their ORCID iD within the submission system before final acceptance, as unverified ORCID iDs will not appear in the published article. *Only*  the individual author can complete the verification step; PLOS staff *cannot*  verify ORCID iDs on behalf of authors.

We look forward to receiving your revised manuscript.

Kind regards,

Zhengmao Li

Academic Editor

PLOS One

Journal Requirements:

1. If the reviewer comments include a recommendation to cite specific previously published works, please review and evaluate these publications to determine whether they are relevant and should be cited. There is no requirement to cite these works unless the editor has indicated otherwise.

2. Please review your reference list to ensure that it is complete and correct. If you have cited papers that have been retracted, please include the rationale for doing so in the manuscript text, or remove these references and replace them with relevant current references. Any changes to the reference list should be mentioned in the rebuttal letter that accompanies your revised manuscript. If you need to cite a retracted article, indicate the article’s retracted status in the References list and also include a citation and full reference for the retraction notice.

[Note: HTML markup is below. Please do not edit.]

Reviewers' comments:

Reviewer's Responses to Questions

**Comments to the Author**

1. If the authors have adequately addressed your comments raised in a previous round of review and you feel that this manuscript is now acceptable for publication, you may indicate that here to bypass the “Comments to the Author” section, enter your conflict of interest statement in the “Confidential to Editor” section, and submit your "Accept" recommendation.

Reviewer #1: All comments have been addressed

Reviewer #3: All comments have been addressed

Reviewer #4: All comments have been addressed

**********

2. Is the manuscript technically sound, and do the data support the conclusions?

The manuscript must describe a technically sound piece of scientific research with data that supports the conclusions. Experiments must have been conducted rigorously, with appropriate controls, replication, and sample sizes. The conclusions must be drawn appropriately based on the data presented.

Reviewer #1: Yes

Reviewer #3: Yes

Reviewer #4: Yes

**********

3. Has the statistical analysis been performed appropriately and rigorously?

Reviewer #1: Yes

Reviewer #3: Yes

Reviewer #4: Yes

**********

4. Have the authors made all data underlying the findings in their manuscript fully available?

The PLOS Data policy requires authors to make all data underlying the findings described in their manuscript fully available without restriction, with rare exception (please refer to the Data Availability Statement in the manuscript PDF file). The data should be provided as part of the manuscript or its supporting information, or deposited to a public repository. For example, in addition to summary statistics, the data points behind means, medians and variance measures should be available. If there are restrictions on publicly sharing data—e.g. participant privacy or use of data from a third party—those must be specified.

Reviewer #1: Yes

Reviewer #3: Yes

Reviewer #4: Yes

**********

5. Is the manuscript presented in an intelligible fashion and written in standard English?

PLOS ONE does not copyedit accepted manuscripts, so the language in submitted articles must be clear, correct, and unambiguous. Any typographical or grammatical errors should be corrected at revision, so please note any specific errors here.

Reviewer #1: Yes

Reviewer #3: Yes

Reviewer #4: Yes

**********

6. Review Comments to the Author

Please use the space provided to explain your answers to the questions above. You may also include additional comments for the author, including concerns about dual publication, research ethics, or publication ethics. (Please upload your review as an attachment if it exceeds 20,000 characters)

Reviewer #1: Thank you for submitting the revised version of the manuscript. I have carefully reviewed the revised paper as well as the point-by-point responses provided by the authors. In my assessment, the authors have addressed all of the comments and concerns raised in the previous round of review in a satisfactory and thoughtful manner.

Reviewer #3: The manuscript has been revised based on the review comments and the responses are convincing. Therefore, the manuscript maybe considered for publication in it's present form.

Reviewer #4: The paper has improved a lot and is technically strong. The authors have clearly addressed the earlier comments well. The method is well explained and the overall idea is good. The work on improving hosting capacity in distribution systems using optimization techniques is useful and relevant for modern power systems. Only a few small improvements are suggested to make the paper clearer and more useful in practice.

1. The authors should briefly explain how the SOA performs when the system becomes larger. It would also help to include a small table or note showing the average time taken for each run so readers can understand how practical the method is.

2. It would be good to add a short explanation for reducing 1,000 scenarios to 10 using the Kantorovich Distance Matrix. The authors may also comment on whether using more scenarios like 20 or 50 changes the results much or if the results stay almost the same.

3. The paper should include a short discussion about real world communication issues like delay, data loss, or communication failure between smart inverters and EV chargers. This will help readers understand how strong the system is in real conditions.

4. For better understanding of the multi objective results, the authors may mark a best compromise solution on the Pareto graph. A simple method like TOPSIS or fuzzy logic can be used so readers can easily see the best balanced point.

5. The conclusion can include one or two lines about future work. The authors may mention that the method can also be used for other grid services like frequency control or black start support. This will make the paper more complete.

Overall, the paper is well written and ready for publication after these small improvements.

**********

7. PLOS authors have the option to publish the peer review history of their article (what does this mean?). If published, this will include your full peer review and any attached files.

If you choose “no”, your identity will remain anonymous but your review may still be made public.

**Do you want your identity to be public for this peer review?**  For information about this choice, including consent withdrawal, please see our Privacy Policy.

Reviewer #1: No

Reviewer #3: No

Reviewer #4: No

**********

[NOTE: If reviewer comments were submitted as an attachment file, they will be attached to this email and accessible via the submission site. Please log into your account, locate the manuscript record, and check for the action link "View Attachments". If this link does not appear, there are no attachment files.]

To ensure your figures meet our technical requirements, please review our figure guidelines: https://journals.plos.org/plosone/s/figures

You may also use PLOS’s free figure tool, NAAS, to help you prepare publication quality figures: https://journals.plos.org/plosone/s/figures#loc-tools-for-figure-preparation.

NAAS will assess whether your figures meet our technical requirements by comparing each figure against our figure specifications.

# Author comment: Author response to Decision Letter 2

**DOI:** 10.1371/journal.pone.0350725.r004

21 Apr 2026

Response to the Reviewer Comments

Dear Editor and Reviewers

The manuscript has been revised based on the precise comments of the respected reviewers. The authors hope the revised version of the manuscript fulfills all expectations of the reviewers and editor. The responses provided by the authors to your comments are as follows:

Reviewer #4

Reviewer #4, Concern #1: The authors should briefly explain how the SOA performs when the system becomes larger. It would also help to include a small table or note showing the average time taken for each run so readers can understand how practical the method is.

Authors’ response:

The authors thank the reviewer for this valuable and practical suggestion. We agree that providing insight into the computational performance and scalability of the proposed algorithm enhances the applicability and transparency of the study.

In response, additional simulations have been conducted to evaluate the execution time of the proposed SFOA-based optimization framework for different system sizes and case studies. The execution time was recorded for Cases 2–4 under representative operating conditions for both the IEEE 33-bus system and the larger Cairo 59-bus distribution system.

The results, now included in the revised manuscript in a dedicated paragraph (subsection 5.3) and summarized in Table 7, show that execution time increases with system size, as expected. Specifically, the runtime increases from approximately 7–9 minutes for the IEEE 33-bus system to about 28–32 minutes for the Cairo 59-bus system. Despite this increase, the overall computational burden remains practical for offline planning studies. These additions provide a clearer understanding of the practical performance of the proposed method and its applicability to larger-scale systems.

Table 7: Computational time of the proposed SOA for different case studies and system sizes, Scenario 5

System Case Time (s)

IEEE 33-bus (h=13) Case 2 427

IEEE 33-bus (h=13) Case 3 432

IEEE 33-bus (h=13) Case 4 547

Cairo 59-bus (h=10) Case 2 1710

Cairo 59-bus (h=10) Case 3 1728

Cairo 59-bus (h=10) Case 4 1895

Authors’ action: A new paragraph and table have been added to report the execution time of the proposed method for different system sizes, demonstrating its computational performance and scalability.

Reviewer #4, Concern #2: It would be good to add a short explanation for reducing 1,000 scenarios to 10 using the Kantorovich Distance Matrix. The authors may also comment on whether using more scenarios like 20 or 50 changes the results much or if the results stay almost the same.

Authors’ response:

The authors sincerely thank the reviewer for this insightful and constructive comment. We agree that providing a clearer explanation of the scenario reduction process and evaluating the impact of the number of representative scenarios enhances the robustness and transparency of the study.

In response, the description of the Kantorovich Distance Matrix (KDM)-based scenario reduction technique has been expanded in the revised manuscript. Additional clarification has been included to better explain how the distance between scenarios is evaluated, how redundant scenarios are iteratively eliminated, and how probability redistribution preserves the statistical characteristics of the original Monte Carlo-generated dataset. The revised text also explicitly highlights that the reduction from 1,000 scenarios to a smaller representative subset is achieved without significant loss of information, while substantially improving computational efficiency.

Second, an additional sensitivity analysis has been conducted by increasing the number of representative scenarios from 10 to 20. The optimization problem (Case 3) was re-evaluated for all scenarios, and the results were compared in terms of hourly DG installed capacity and EV charging demand.

The findings, presented in the revised manuscript (Figs. 21 and 22), demonstrate that the results obtained using 10 and 20 scenarios are highly consistent. The average hourly profiles are nearly identical, and the differences in variability are minimal. Specifically, the aggregated standard deviation over the 24-hour period shows only a marginal increase when using 20 scenarios (DG: 14.6166 to 14.6749; EV demand: 4.1558 to 4.1768), confirming that the additional scenarios provide only limited improvement in representing system uncertainty. In contrast, the computational burden increases significantly, with execution time approximately doubling when using 20 scenarios. This confirms that using 10 representative scenarios provides a sufficiently accurate representation of system uncertainty while maintaining computational efficiency.

(a)

(b)

Fig. 21. Comparative results derived from the stochastic framework across Case 3 of the IEEE 33-bus system under 20 representative scenarios: (a) hourly recorded DG installed capacity, (b) hourly recorded EV stations energy.

(a)

(b)

Fig. 22. Average value of hourly-basis results for 10 and 20 representative scenarios: (a) DG installed capacity, (b) EV stations energy.

Authors’ action: The explanation of the KDM-based scenario reduction method has been expanded. Additionally, a sensitivity analysis using 20 representative scenarios has been included, supported by Figs. 21 and 22 and quantitative metrics, demonstrating that increasing the number of scenarios has a negligible impact on results while significantly increasing computational cost; thus, the use of 10 scenarios is justified.

Reviewer #4, Concern #3: The paper should include a short discussion about real world communication issues like delay, data loss, or communication failure between smart inverters and EV chargers. This will help readers understand how strong the system is in real conditions.

Authors’ response: The authors sincerely thank the reviewer for this insightful and practical suggestion. We fully agree that considering real-world communication constraints enhances the applicability and robustness of the proposed framework. In response, a new subsection titled “5.3 Practical Communication Considerations and System Robustness” has been added to Section 5 in the revised manuscript. This subsection discusses the potential impacts of communication delays, data loss, and temporary communication failures on coordination between smart inverters and the EV charging infrastructure. Specifically, the discussion highlights that the proposed stochastic optimization framework operates on an hourly timescale and relies on probabilistic, scenario-based modeling, thereby inherently reducing sensitivity to short-term communication delays. Additionally, the roles of data-handling strategies (e.g., buffering and interpolation) and local autonomous control mechanisms (such as Volt/VAR control) are emphasized as key factors that ensure system reliability under imperfect communication conditions. Finally, the manuscript now acknowledges that integrating detailed communication network modeling and cyber-physical co-simulation constitutes an important avenue for future work.

Authors’ action: A new subsection titled “5.3 Practical Communication Considerations and System Robustness” has been added to the revised manuscript. This subsection briefly discusses the impact of communication delays, data loss, and potential communication failures between smart inverters and EV charging infrastructure. It also clarifies the inherent robustness of the proposed stochastic framework, owing to its scenario-based nature, and highlights the role of local control strategies in maintaining reliable operation under imperfect communication conditions.

Reviewer #4, Concern #4: For better understanding of the multi objective results, the authors may mark a best compromise solution on the Pareto graph. A simple method like TOPSIS or fuzzy logic can be used so readers can easily see the best balanced point.

Authors’ response:

The authors sincerely thank the reviewer for this valuable suggestion. We fully agree that identifying the best-compromise solution significantly enhances the clarity and practical interpretation of multi-objective optimization results.

In response, a new subsection titled “5.5. Identification of the Best Compromise Solution Using TOPSIS” has been added to the revised manuscript. In this subsection, the Technique for Order Preference by Similarity to Ideal Solution (TOPSIS) is employed as a multi-criteria decision-making tool to systematically rank all weighting-coefficient combinations.

The analysis considers multiple performance criteria, including DG installed capacity and EV charging demand as benefit criteria, and voltage deviation, power losses, and constraint violations as cost criteria. All criteria are normalized and equally weighted to ensure an unbiased comparison. A penalty term is incorporated to account for infeasible solutions, ensuring they are appropriately penalized without being excluded.

The results demonstrate that the proposed weighting combination achieves the highest closeness coefficient (≈ 0.69), identifying it as the most balanced solution among all alternatives. To further improve visualization and reader understanding, a new figure (Fig. 20) has been added to the revised manuscript, illustrating the TOPSIS-based ranking and clearly highlighting the selected best-compromise solution.

Fig. 20. TOPSIS ranking of weighting-coefficient combinations, highlighting the proposed solution as the best compromise.

Authors’ action: A new subsection (Subsection 5.5) and an additional figure (Fig. 20) have been included in the revised manuscript to present a TOPSIS-based analysis, identifying and highlighting the best compromise solution among the considered weighting combinations.

Reviewer #4, Concern #5: The conclusion can include one or two lines about future work. The authors may mention that the method can also be used for other grid services like frequency control or black start support. This will make the paper more complete.

Authors’ response:

The authors thank the reviewer for this valuable suggestion.

The conclusion Section has been amended to include a brief discussion on potential future extensions of the developed stochastic optimization framework toward additional grid services. Specifically, it is now indicated that coordinated control of DG and EV charging systems can be adapted to support services such as frequency control through active power management and system restoration during black-start conditions. The revised text also notes that such applications would require incorporating dynamic modeling and multi-timescale control strategies.

Authors’ action: Future work paragraph has been added to the conclusion, highlighting the potential extension of the proposed framework to support grid services such as frequency control and black start operations.

-------------------------------------------------------------------------------------------------------------------

Finally, the authors hope that the revised version of the manuscript meets all expectations of the reviewers and the editor.

Attachment Submitted filename: Reviewers comments.docx

# Peer review: Decision Letter 2

**DOI:** 10.1371/journal.pone.0350725.r005

**Authors:** Zhengmao Li

18 May 2026

Joint Optimization of Smart Inverters and EV Charging Coordination for Enhanced DG-EV Hosting Capacity Under Uncertain Conditions for Resilient Distribution Systems

PONE-D-25-59694R2

Dear Dr. Ali,

We’re pleased to inform you that your manuscript has been judged scientifically suitable for publication and will be formally accepted for publication once it meets all outstanding technical requirements.

Within one week, you’ll receive an e-mail detailing the required amendments. When these have been addressed, you’ll receive a formal acceptance letter and your manuscript will be scheduled for publication.

An invoice will be generated when your article is formally accepted. Please note, if your institution has a publishing partnership with PLOS and your article meets the relevant criteria, all or part of your publication costs will be covered. Please make sure your user information is up-to-date by logging into Editorial Manager at Editorial Manager® and clicking the ‘Update My Information' link at the top of the page. For questions related to billing, please contact billing support.

If your institution or institutions have a press office, please notify them about your upcoming paper to help maximize its impact. If they’ll be preparing press materials, please inform our press team as soon as possible -- no later than 48 hours after receiving the formal acceptance. Your manuscript will remain under strict press embargo until 2 pm Eastern Time on the date of publication. For more information, please contact onepress@plos.org.

Kind regards,

Zhengmao Li

Academic Editor

PLOS One

Additional Editor Comments (optional):

Reviewers' comments:

Reviewer's Responses to Questions

**Comments to the Author**

1. If the authors have adequately addressed your comments raised in a previous round of review and you feel that this manuscript is now acceptable for publication, you may indicate that here to bypass the “Comments to the Author” section, enter your conflict of interest statement in the “Confidential to Editor” section, and submit your "Accept" recommendation.

Reviewer #4: All comments have been addressed

**********

2. Is the manuscript technically sound, and do the data support the conclusions?

The manuscript must describe a technically sound piece of scientific research with data that supports the conclusions. Experiments must have been conducted rigorously, with appropriate controls, replication, and sample sizes. The conclusions must be drawn appropriately based on the data presented.

Reviewer #4: Yes

**********

3. Has the statistical analysis been performed appropriately and rigorously?

Reviewer #4: Yes

**********

4. Have the authors made all data underlying the findings in their manuscript fully available?

The PLOS Data policy requires authors to make all data underlying the findings described in their manuscript fully available without restriction, with rare exception (please refer to the Data Availability Statement in the manuscript PDF file). The data should be provided as part of the manuscript or its supporting information, or deposited to a public repository. For example, in addition to summary statistics, the data points behind means, medians and variance measures should be available. If there are restrictions on publicly sharing data—e.g. participant privacy or use of data from a third party—those must be specified.

Reviewer #4: Yes

**********

5. Is the manuscript presented in an intelligible fashion and written in standard English?

PLOS ONE does not copyedit accepted manuscripts, so the language in submitted articles must be clear, correct, and unambiguous. Any typographical or grammatical errors should be corrected at revision, so please note any specific errors here.

Reviewer #4: Yes

**********

6. Review Comments to the Author

Please use the space provided to explain your answers to the questions above. You may also include additional comments for the author, including concerns about dual publication, research ethics, or publication ethics. (Please upload your review as an attachment if it exceeds 20,000 characters)

Reviewer #4: The authors have significantly improved the paper in this revision. The addition of Subsection 5.3 and Table 7 helps clearly explain the computational performance of the Starfish Optimization Algorithm across different test systems. The clarification of the Kantorovich Distance Matrix and the sensitivity analysis improves the technical strength of the scenario reduction approach. The inclusion of communication robustness considerations and the TOPSIS based decision making method also improves the practical relevance of the work. Overall, the paper is now clearer, more complete, and technically stronger. It is suitable for publication.

**********

7. PLOS authors have the option to publish the peer review history of their article (what does this mean?). If published, this will include your full peer review and any attached files.

If you choose “no”, your identity will remain anonymous but your review may still be made public.

**Do you want your identity to be public for this peer review?**  For information about this choice, including consent withdrawal, please see our Privacy Policy.

Reviewer #4: No

**********

# Editor report: Acceptance letter

**DOI:** 10.1371/journal.pone.0350725.r006

**Authors:** Zhengmao Li

PONE-D-25-59694R2

PLOS One

Dear Dr. Ali,

I'm pleased to inform you that your manuscript has been deemed suitable for publication in PLOS One. Congratulations! Your manuscript is now being handed over to our production team.

At this stage, our production department will prepare your paper for publication. This includes ensuring the following:

* All references, tables, and figures are properly cited

* All relevant supporting information is included in the manuscript submission,

* There are no issues that prevent the paper from being properly typeset

You will receive further instructions from the production team, including instructions on how to review your proof when it is ready. Please keep in mind that we are working through a large volume of accepted articles, so please give us a few days to review your paper and let you know the next and final steps.

Lastly, if your institution or institutions have a press office, please let them know about your upcoming paper now to help maximize its impact. If they'll be preparing press materials, please inform our press team within the next 48 hours. Your manuscript will remain under strict press embargo until 2 pm Eastern Time on the date of publication. For more information, please contact onepress@plos.org.

You will receive an invoice from PLOS for your publication fee after your manuscript has reached the completed accept phase. If you receive an email requesting payment before acceptance or for any other service, this may be a phishing scheme. Learn how to identify phishing emails and protect your accounts at https://explore.plos.org/phishing.

If we can help with anything else, please email us at customercare@plos.org.

Thank you for submitting your work to PLOS ONE and supporting open access.

Kind regards,

PLOS ONE Editorial Office Staff

on behalf of

Dr Zhengmao Li

Academic Editor

PLOS One
